# Zinc Affinity of Benzamide-Based Histone Deacetylase Inhibitors: A DFT Study

**DOI:** 10.3390/molecules31101650

**Published:** 2026-05-14

**Authors:** Nikolay Toshev, Kristiyan Velichkov, Yordanka Uzunova, Diana Cheshmedzhieva, Todor Dudev

**Affiliations:** 1 Department of Bioorganic Chemistry, Faculty of Pharmacy, Medical University of Plovdiv, 15A Vassil Aprilov Blvd., 4002 Plovdiv, Bulgaria; yordanka.uzunova@mu-plovdiv.bg; 2 Medical Faculty, Medical University of Plovdiv, 15A Vassil Aprilov Blvd., 4002 Plovdiv, Bulgaria; kristiyan.velichkov4@gmail.com; 3 Research Institute, Medical University of Plovdiv, 15A Vassil Aprilov Blvd, 4002 Plovdiv, Bulgaria; 4 Department of Pharmaceutical and Applied Organic Chemistry, Faculty of Chemistry and Pharmacy, Sofia University “St. Kliment Ohridski”, 1 James Bourchier Blvd., 1164 Sofia, Bulgaria; dvalentinova@gmail.com (D.C.); ohtttd@chem.uni-sofia.bg (T.D.)

**Keywords:** zinc affinity, DFT study, benzamide-based HDAC inhibitors, metal coordination, zinc-binding group, Chidamide, Mocetinostat, Zabadinostat, Entinostat, Tacedinaline

## Abstract

Histone deacetylase inhibitors (HDACi) are an emerging class of epigenetic anticancer drugs that exert their activity through coordination to the catalytic Zn^2+^ ion within the active site of histone deacetylases (HDACs). Due to the limited isoform-selectivity of hydroxamic acid-based inhibitors, benzamide-based HDACi (BBHDACi) have been developed as subtype-selective alternatives. Clinically relevant representatives include Chidamide, Entinostat, Mocetinostat, Zabadinostat, and Tacedinaline. Although these compounds share a conserved o-aminoanilide zinc-binding group (ZBG), they differ in linker and cap region structure, raising questions regarding their intrinsic Zn^2+^ affinity and coordination behavior. Herein, density functional theory (DFT) calculations were performed at the B3LYP/6-311++g(d,p) level of theory combined with the PCM solvation in methanol (ε = 33) and water (ε = 78). Geometry optimization confirmed that the trans (E) isomer of Chidamide is thermodynamically preferred. Coordination studies showed that the remaining BBHDACi adopt stable geometries, with the o-aminoanilide group preferentially forming tetracoordinated complexes that are more stable than hexacoordinated ones in polar media. Interestingly, calculated substitution free energies differed by less than ± 2 kcal.mol^−1^, indicating nearly identical intrinsic Zn^2+^ affinities across the series. These results suggest that the ZBG contributes similarly to metal coordination across all BBHDACi, whereas the overall binding strength is mainly governed by interactions of the linker and cap regions rather than by the conserved zinc-binding group itself.

## 1. Introduction

### 1.1. Zinc-Dependent Histone Deacetylases in Cancer: Structural Features and Inhibitor Recognition

Cancer is a disease characterized by uncontrolled cell proliferation, genomic instability, and dysregulated gene expression, often leading to disease progression and poor prognosis [1]. Epigenetic mechanisms regulate gene expression without changing the DNA sequence through reversible chemical modifications to the chromatin structure, thereby preserving cellular identity and genomic integrity [2]. Among epigenetic mechanisms, post-translational modifications (PTMs) of histone proteins are of particular interest, as they provide a link between the protein structure and transcriptional regulation. Histones, the fundamental building blocks of chromatin structure, undergo different reversible PTMs [3]. Their N-terminal tails are prone to acetylation, methylation, phosphorylation, and ubiquitination [4]. These PTMs influence chromatin accessibility and modulate transcriptional gene activation and repression. Dysregulation of histone-modifying enzymes affects chromatin homeostasis and leads to aberrant gene expression, which is associated with disease development, including cancer [5]. Over the past two decades, histone modifications have been intensively studied in cancer research, as their dysregulation contributes to cancer progression [6].

Histone deacetylases (HDACs) regulate histone acetylation, catalyzing the removal of acetyl groups from lysine residues in both histone and non-histone proteins, thereby acting as key epigenetic regulators [7]. This deacetylation makes chromatin more compact, thus leading to transcriptional repression [8]. Moreover, numerous studies have linked abnormal HDAC expression with transcriptional repression of tumor suppressor genes and altered cell-cycle regulation, differentiation, and apoptosis pathways in many cancers [7,9,10]. In cancer, the balance between histone acetyl transferases (HATs) and HDACs activity is often disrupted. Overexpression or hyperactivation of HDACs leads to hypoacetylation and inappropriate gene silencing responsible for growth control and apoptosis [11]. The inhibition of metalloenzymes has emerged as a therapeutic approach in modern medicine over the past two decades, with numerous drugs exploiting this principle, as these enzymes play a crucial role in various biological processes and are directly involved in disease development and progression [12]. The human HDAC family consists of 18 enzymes, grouped into four classes [13]. Classes I, II, and IV are classified as zinc-dependent HDACs, as their catalytic activity requires a Zn^2+^ ion, whereas class III enzymes are NAD+ dependent. The Class I HDACs include HDAC1, HDAC2, HDAC3, and HDAC8. Class II HDACs are subdivided into class IIa (HDAC4, HDAC5, HDAC7, and HDAC9) and class IIb (HDAC6 and HDAC10). Class IV includes only one member, HDAC11, which shares structural features with both class I and class II enzymes. Beyond their classification, the catalytic structure of Zn^2+^-dependent HDACs provides the structural basis for inhibitor recognition and selectivity. These enzymes have a conserved active site with a narrow hydrophobic tunnel that leads to the Zn^2+^ ion [14,15]. The conserved HDAC structure of the catalytic pocket is important for the binding mode of structurally diverse histone deacetylase inhibitors (HDACi). Because classes I, II, and IV are Zn^2+^-dependent metalloenzymes, coordination to the catalytic zinc ion represents a crucial factor governing inhibitor binding, potency, and isoform-selectivity. These insights have positioned Zn^2+^-dependent HDACs as promising therapeutic targets, leading to the development of new epigenetic drugs designed to bind and inhibit their activity [16,17].

### 1.2. Chemical Classification of HDACi

HDACi represent a structurally diverse group of small molecules that target the HDAC enzymatic activity [18]. According to their chemical structure, they are classified into the following major chemical classes: hydroxamic acids [19], benzamides [20], short-chain fatty acids [21], cyclic tetrapeptides [22], and others [23,24,25]. Hydroxamic acid-based histone deacetylase inhibitors represent the first generation of approved drugs and remain the most extensively studied class of HDACi. Representative hydroxamic acid derivatives, including Vorinostat, Belinostat, and Panobinostat, all of which exhibit strong zinc-binding properties and broad-spectrum HDAC activity through bidentate coordination to the catalytic Zn^2+^ ion, thereby blocking enzyme activity [18]. Recent studies by Pflum and co-workers have investigated the structural requirements of HDAC inhibitors, showing that modifications of the linker, cap group, and the metal-binding warhead of hydroxamic acid SAHA analogues can modulate isoform selectivity and pharmacological properties, thereby providing valuable insights into rational inhibitor design [26,27,28,29]. Although hydroxamic-acid-based inhibitors share a similar scaffold, their limited isoform selectivity is associated with off-target effects and toxicities. Moreover, hydroxamate-based inhibitors have poor metabolic stability, thus decreasing their therapeutic index [30]. The majority of current HDACi act as pan-HDAC inhibitors, targeting and inhibiting multiple HDAC isoforms; thus, the lack of isoform-specificity contributes to adverse drug effects [31]. These limitations have refocused scientific efforts on the development of second-generation HDACi with improved isoform selectivity and pharmacokinetic profiles.

### 1.3. Benzamide-Based HDAC Inhibitors

**Scheme 1:**
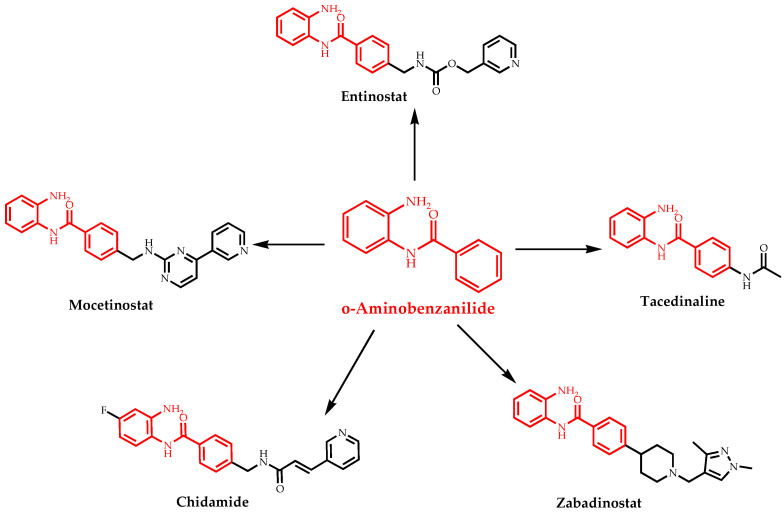
Structures of the studied BBHDACi.

They represent a second-generation class of epigenetic drugs containing a ZBG linked to an aromatic cap region through an amide-based linker, enabling selective coordination to the catalytic Zn^2+^ ion [18]. Compared with hydroxamic acid derivatives, benzamides exhibit greater selectivity toward class I HDAC isoforms, likely due to their distinct coordination of the catalytic Zn^2+^ ion and binding mode within the catalytic pocket [32,33]. The improved isoform selectivity of benzamide-based histone deacetylase inhibitors (BBHDACi) contributes to a potentially increased therapeutic index, thus positioning them as promising candidates for epigenetic therapy. In this context, BBHDACi have attracted significant scientific attention over the last ten years. Clinically relevant representatives include Chidamide, Entinostat, Mocetinostat, Zabadinostat, and Tacedinaline. Information regarding these compounds is summarized in Table 1, and their structures are shown in Scheme 1.

### 1.4. Pharmacophore Model of Benzamide-Based HDAC Inhibitors

**Figure 1:**
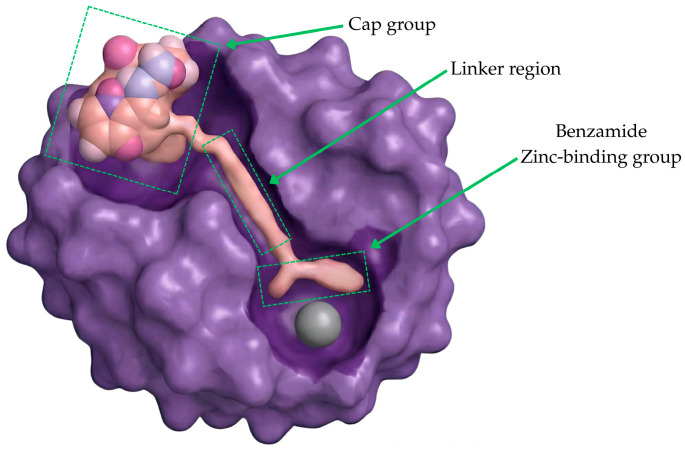
Pharmacophore model of BBHDACi (created in NotebookLM and refined in Canva, Accessed on 3 March 2026).

The pharmacophore model of BBHDACi is composed of three structural elements: (1) a zinc-binding group (ZBG), (2) an aromatic or amide-based linker region, and (3) a cap group that interacts with the surface. The elements of the pharmacophoric model are shown in Figure 1.

The benzamide ZBG contains a carbonyl-containing amide moiety, in which the carbonyl oxygen, together with the adjacent amide nitrogen, is appropriately oriented within the HDAC active site to coordinate the catalytic Zn^2+^ ion. The central linker, typically composed of aromatic or amide-based fragments, provides a relatively rigid and planar connection that preserves π-conjugation and positions the pharmacophoric elements along the catalytic tunnel. The cap group, commonly composed of aromatic and heteroaromatic moieties, governs the isoform selectivity by interacting with residues at the rim of the enzyme pocket through hydrophobic interactions and π–π stacking [14,42]. The central role of metal-binding pharmacophores (MBPs) in the development of metalloenzyme inhibitors similar to benzamides has been extensively studied by Cohen and co-workers [12,43,44,45,46]. They have demonstrated that the nature of the zinc-binding group governs metal coordination, inhibitor binding affinity, and pharmacological properties [43]. These studies further demonstrate that the zinc-binding warhead can be rationally modified to improve the drug-like profile of metalloenzyme inhibitors by adjusting key physicochemical properties such as acidity, lipophilicity, and selectivity [43,45]. Furthermore, bioinorganic model systems and computational techniques, such as density functional theory, have shown their usefulness for gaining deeper understanding of metal–ligand interactions [46].

### 1.5. Structural Role of the o-Aminobenzanilide Scaffold in Benzamide-Based HDAC Inhibitors

The five BBHDACi investigated in this study share a common o-aminobenzanilide scaffold, acting as a ZBG and mediating coordination to the catalytic Zn^2+^ ion within the HDAC enzyme site [32]. This scaffold represents the main pharmacophoric core of the studied compounds and was selected as the reference framework for comparative analysis. Within the studied reference scaffold, the ZBG consists of an amide moiety bearing carbonyl oxygen, where the amide carbonyl oxygen and the adjacent amide nitrogen participate in coordination with the catalytic Zn^2+^ ion. This interaction stabilizes the BBHDACi within the enzyme active site and governs the overall binding mode.

To enable comparative analysis of zinc coordination properties, a simplified model of zinc-binding group was employed in the study. The compound o-amiobenzanilide (o-ABA) was chosen as a reference structure, representing the minimal structural fragment of the o-aminoanilide (o-AA) zinc-binding group. Note that this model maintains the key coordination elements responsible for Zn^2+^ binding- the amide carbonyl oxygen and the amino group in the o-position in the benzene ring. This model eliminates additional complexity that derives from the linker and cap regions. Thus, the o-ABA scaffold is a suitable minimal model of the ZBG, enabling systematic comparison of Zn^2+^ coordination features of the BBHDACi studied in this work.

Notably, the ZBG remains conserved across all selected inhibitors, while significant differences are observed in the linker and its length, as well as in the heterocyclic scaffold in the cap regions. These modifications are key factors that determine and govern isoform selectivity and binding affinity across all HDAC family members. For example, Entinostat and Mocetinostat have more extended aromatic systems with additional heteroatoms, thus resulting in increased π-conjugation and electronic delocalization compared with Chidamide. Moreover, such structural extension may also improve π–π stacking interactions and hydrogen bonding within the HDAC enzyme active site, thus modifying enzyme–inhibitor stabilization within the enzyme pocket.

However, despite the structural similarity of the ZBG across the series of clinically relevant BBHDACi, a systematic thermodynamic evaluation of how these variations in the linker and cap regions influence electronic behavior and intrinsic Zn^2+^ affinity remains unclear. Therefore, the aim of the current study is to comparatively evaluate five selected BBHDACi using DFT calculations to determine how structural modifications beyond the conserved ZBG group affect their electronic properties and metal-binding behavior. In particular, we address whether differences in overall binding strength arose predominantly from the linker and cap regions or from intrinsic changes in Zn^2+^-ZBG interactions.

## 2. Results and Discussion

### 2.1. Z/E Isomerism of Chidamide

**Figure 2:**
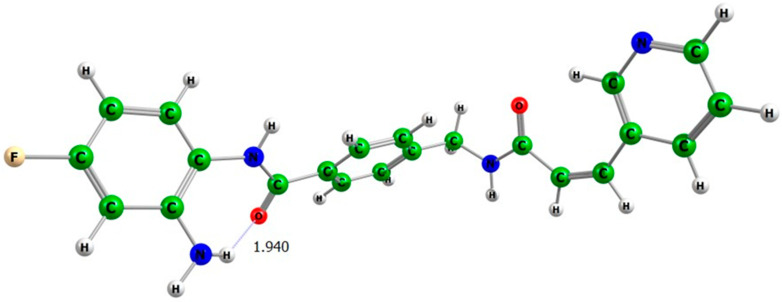
B3LYP/6-311++g(d,p) optimized structure of 1Z-Chidamide and 1E in water.

**Figure 3:**
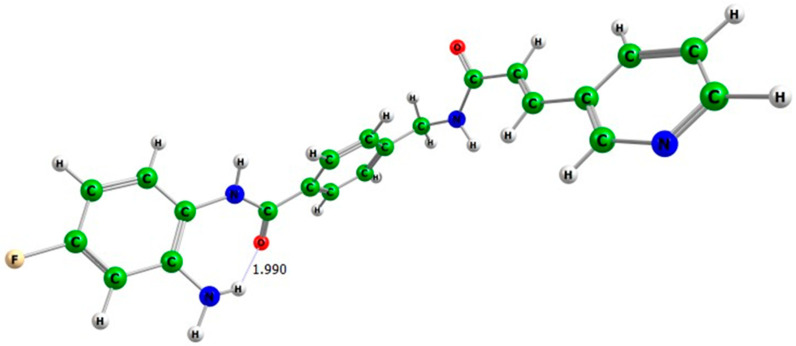
B3LYP/6-311++g(d,p) optimized structure of 1E-Chidamide in water.

Chidamide exists as two stereoisomers: cis (Z) and trans (E). The optimized geometries of both isomers are shown in Figure 2 and Figure 3, respectively.

The calculated relative Gibbs free energies are summarized in Table 2. In the gas phase, the 1E isomer is more stable than the 1Z form by 3.34 kcal.mol^−1^, while in methanol and water, the stabilization increases to 4.48 and 3.86 kcal.mol^−1^, respectively. The slightly larger stabilization in methanol suggests that solvent polarity contributes to additional conformational stabilization.

These results reveal a structural preference for the trans (1E) isomer across all studied environments. The lower Gibbs free energy suggests a more favorable intramolecular arrangement, resulting in lower steric repulsion and improved electronic delocalization within the benzamide framework of Chidamide. These DFT results agree with the previously reported study, which identified the trans isomer as the more stable form [47]. Therefore, the 1E isomer was chosen as the representative stereoisomer for subsequent zinc coordination and metal-binding studies.

### 2.2. Structural and Solvation Analysis of BBHDACi

The molecular geometries of the o-ABA, Entinostat, Mocetinostat, Tacedinaline, and Zabadinostat were optimized using the same DFT methodology as Chidamide, ensuring a consistent computational methodology across the studied inhibitors. The optimized geometries of the studied molecules are shown in the Supplementary Materials (Figures S1–S5).

To the best of our knowledge, this represents the first DFT study of the optimized geometries of these clinically relevant inhibitors.

All optimized structures exhibit stable conformations characterized by a planar benzamide core and a well-defined spatial arrangement of the amide carbonyl oxygen and amide nitrogen, suitable for zinc coordination. Geometry optimization revealed that all studied BBHDACi retain a planar benzamide core, which ensures preservation of π-conjugation and appropriate positioning of donor atoms for zinc binding. Despite differences in linker structure and cap group position, the conserved geometry of the reference o-aminoanilide scaffold represents a structural unit suitable for coordination with the catalytic Zn^2+^ ion. Intramolecular hydrogen bond is observed in Mocetinostat (≈1.99 Å), contributing to conformational rigidity and stabilization of the zinc-binding region. Mocetinostat features a planar amide bond connected to conjugated aromatic moieties, forming an extended π-delocalized system enriched with heteroatoms. Entinostat also has an extended aromatic system that enhances π-conjugation and solvent accessibility. Its heteroatom-rich and electronically delocalized structure promotes strong interactions in polar media. Upon geometry optimization, Tacedinaline adopts a stable conformation characterized by a planar benzamide core and donor atoms properly oriented for metal coordination. In contrast, Zabadinostat has a well-defined benzamide core; however, compared with Entinostat and Mocetinostat, it has reduced conjugation due to higher conformational flexibility in its cap region.

The calculated relative solvation Gibbs energies are shown in Table 3. The calculated relative solvation Gibbs free energies illustrate differences in solvent stabilization within the series. As expected, all molecules exhibit significant stabilization in polar media. The reference o-ABA shows moderate solvation stabilization (∆G33= −8.74 kcal.mol^−1^ and ∆G78 = −9.87 kcal.mol^−1^), compared with the other BBHDACi, which have significantly more negative values.

Among the studied molecules, Entinostat showed the largest stabilization (∆G^33^ = −15.48 kcal.mol^−1^ and ∆G^78^ = −16.14 kcal.mol^−1^), followed by Mocetinostat (∆G^33^ = −13.48 kcal.mol^−1^ and ∆G^78^ = −13.95 kcal.mol^−1^) and Tacedinaline (∆G^33^ = −13.51 kcal.mol^−1^ and ∆G^78^ = −13.96 kcal.mol^−1^), with comparable values, while Zabadinostat (∆G^33^ = −12.41 kcal.mol^−1^ and ∆G^78^ = −13.36 kcal.mol^−1^) showed slightly lower stabilization within the inhibitor series. Only minor differences between methanol and water were observed, thus suggesting a similar solvent effect in both media. These results show that larger molecules, expanded aromatic systems, and the presence of additional heteroatoms improve solvent stabilization in polar environments.

### 2.3. Binding Mode of BBHDACi

#### 2.3.1. Tetracoordinated Complexes

The calculated Gibbs free energy changes for the formation of tetracoordinated Zn^2+^ complexes with o-ABA, Chidamide, Entinostat, Mocetinostat, Tacedinaline and Zabadinostat are summarized in Table 4.

All reactions show negative ∆G values, indicating that the coordination of BBHDACi to the Zn^2+^ ion is thermodynamically favorable for all studied ligands in both the gas phase and in condensed media.

In the gas phase (∆G^1^), the Zn^2+^ binding strength follows the trend: Tacedinaline < Zabadinostat < Mocetinostat < Entinostat < Chidamide < o-ABA, with o-ABA having the highest complexation energy (−19.85 kcal.mol^−1^) and thus having the lowest metal affinity in the gas phase.

In methanol ∆G^33^, all studied molecules had comparable Zn^2+^ affinities, with ∆G values ranging from −34.43 to −35.91 kcal.mol^−1^. Chidamide exhibits the most favorable binding (−35.91 kcal.mol^−1^). So, as a result, in methanol, whose dielectric constant can serve as an approximate model in the protein cavity environment [48], the Zn^2+^ binding strength follows the increasing order of affinity: Mocetinostat < o-ABA < Entinostat < Zabadinostat < Tacedinaline < Chidamide.

Not surprisingly, a similar trend toward favorable coordination is observed in water, where ∆G^78^ values range within a narrow interval from −36.47 to −38.19 kcal.mol^−1^. Moreover, calculations in water reveal that the binding energy difference between BBHDACi decreases, thus indicating almost equal metal affinities in water.

Solvation effects provide substantial stabilization of the complexes, as reflected by the more negative ∆G^33^ and ∆G^78^ values, thus demonstrating that polar media lower the ligand-binding energies.

Overall, these results shed more light on the binding mode of BBHDACi, confirming that they possess a strong and comparable ability to coordinate to the Zn^2+^ ion at physiological conditions, which is consistent with their role as zinc-dependent inhibitors. Moreover, the modest variations observed in polar media underscore the role and importance of solvation effects in analyzing metal-binding trends, providing a basis for comparative metal-affinity analysis within the BBHDACi.

**Figure 4:**
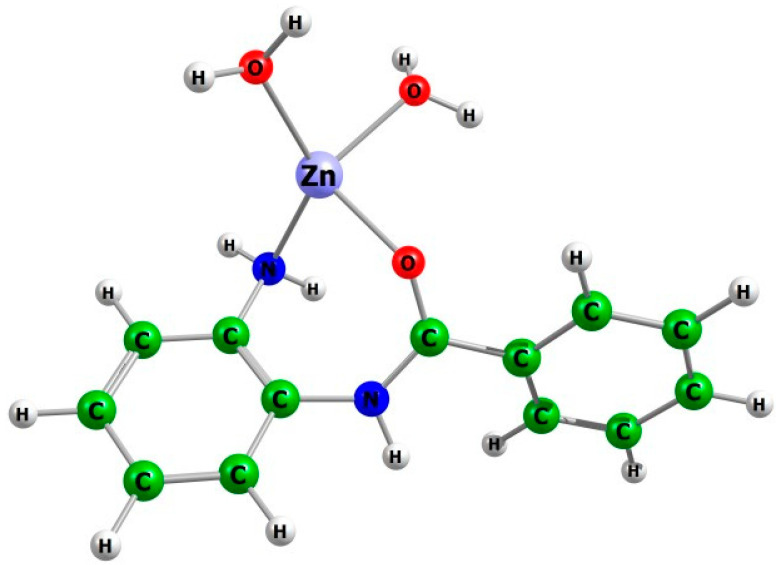
B3LYP/6-311++g(d,p) optimized structure of the tetracoordinated [o-ABA-Zn(H_2_O)_2_]^2+^ complex in water.

The optimized geometry of the representative [o-ABA-Zn(H_2_O)_2_]^2+^ complex in water is presented in Figure 4.

To support the calculated thermodynamic trend, we examined the Zn-inhibitor bond lengths in the optimized tetracoordinated species, [BBHDACi-Zn(H_2_O)_2_]^2+^. This analysis provided more insight into the relative coordination strength of the studied BBHDACi towards the Zn^2+^ ion. Results from bond length analysis are presented in Table 5.

In these complexes, the Zn-O^1^ bond corresponds to coordination with the carbonyl oxygen atom, while the Zn-N^1^ bond comes from the amino group of the o-aminoanilide moiety. The coordinated water molecules are denoted W^1^ and W^2^, with W^1^ closer to the amino group and W^2^ being closer to the carbonyl group.

Across the series, the Zn-O^1^ bond distances range from 2.00 to 2.07 Å. The reference o-ABA exhibits a Zn-O^1^ bond length of 2.04 Å, whereas Entinostat showed a slightly shorter distance (2.02 Å), thus suggesting a stronger coordination of the carbonyl oxygen to the Zn^2+^ ion. Mocetinostat and Tacedinaline display Zn-O^1^ distances in the range of 2.00 to 2.03 Å, indicating a comparable binding strength. In contrast, Chidamide showed a slightly longer Zn-O^1^ bond (2.07 Å), indicating weaker coordination in comparison.

The Zn-N^1^ bond lengths fall within 2.08–2.11 Å across all studied complexes, confirming stable bidentate coordination through the reference scaffold. Moreover, the close similarity in these distances gives us insight into the fact that the donor nitrogen atom has consistent coordination geometry, despite structural differences among the studied molecules.

To enable comparison with experimental data, the calculated Zn-N and Zn-O bond lengths were compared with those from the crystallographic HDAC benzamides complex (PDB code: 4LY1, [49]), which represents a structurally similar system that contains the o-aminoanilide moiety. It should be noted that the calculated Zn-N bond length of approximately 2.10 Å is in good agreement with experimental crystallographic HDAC benzamide complexes (PDB code: 4LY1), where Zn-N bond length ranges from 1.91 to 2.19 Å (mean ≈ 2.10 Å), across chains A-C, thus validating the choice of functional and basis set for describing metal–ligand coordination. However, the calculated Zn-O distance (2.02–2.04 Å) across the series of benzamide-based HDACs are shorter than the experimental results reported for 4LY1, ranging from 2.55–2.62 Å (mean ≈ 2.59 Å) across chains A-C.

This difference can be explained by the use of a simplified binding model in the present study, in which the metal ion is coordinated to the benzamide ligand and two water molecules, without inclusion of the protein environment. Generally, in the HDAC catalytic site, the carbonyl oxygen is involved in an extended hydrogen-bonding system, with nearby residues, as well as interactions within the channel [50,51]. The absence of these stabilizing interactions in the simplified model of enzyme–inhibitor interactions allows the ligand to adopt different geometry, resulting in shorter Zn-O bond distances.

The Zn-W^1^ and Zn-W^2^ bond lengths corresponding to coordinated water molecules range from 2.05 to 2.11 Å. In complexes where Zn-inhibitor interactions are stronger, the distance between the metal ion and water is longer. Notably, Mocetinostat shows a Zn-W^1^ bond length of 2.11 Å, which suggests partial displacement because of stronger coordination.

Overall, the bond-length analysis supports the thermodynamic stabilization trends observed in the substitution reactions. Thus, shorter Zn-O^1^ bond distances correlate with increased stability, while slightly longer bond distances suggest reduced electronic delocalization or conformational flexibility. The calculated Zn-N^1^ and Zn-O^1^ bond lengths (2.00–2.10 Å) fall within the typical range reported for Zn^2+^ ion coordination in metalloenzymes [52], confirming the reliability of the optimized geometries and proposed models.

#### 2.3.2. Hexacoordinated Complexes

Consistent with the calculated results for the tetracoordinated complexes, all BBHDACi have negative ∆G values, confirming thermodynamically favorable coordination across the studied molecules. The calculated Gibbs free energy changes for the formation of hexacoordinated Zn^2+^ complexes with o-ABA, Chidamide, Entinostat, Mocetinostat, Tacedinaline, and Zabadinostat are summarized in Table 6.

**Figure 5:**
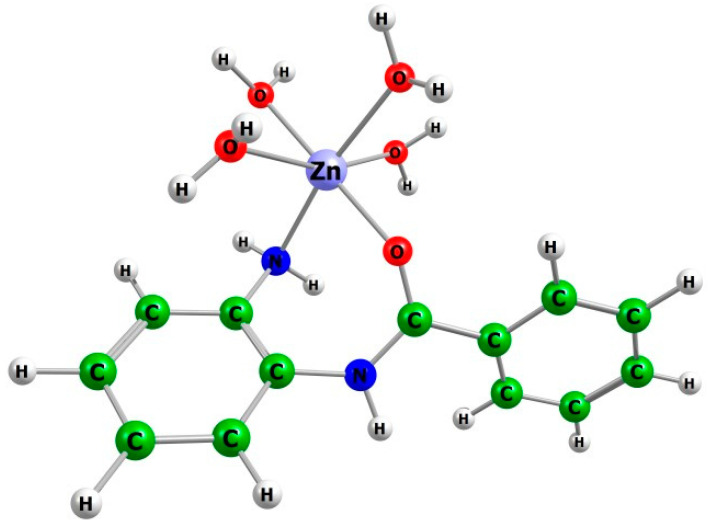
B3LYP/6-311++g(d,p) optimized structure of the hexacoordinated [OAB-Zn(H_2_O)_4_]^2+^ complex in water.

In the gas phase (∆G^1^), formation of hexacoordinated species is strongly exergonic for all inhibitors, with ∆G values ranging from −34.98 to −52.2 kcal.mol^−1^. Among the investigated inhibitors, Entinostat shows the most favorable Zn^2+^ binding affinity in this coordination mode, followed by Zabadinostat, Chidamide, Tacedinaline, Mocetinostat, and o-ABA. In contrast, inclusion of solvation significantly reduces binding free energy in polar media. In methanol (∆G^33^), ∆G values are −8.78 kcal.mol^−1^ for Chidamide, −12.65 kcal.mol^−1^ for Entinostat, −15.60 kcal.mol^−1^ for o-ABA, −15.72 kcal.mol^−1^ for Mocetinostat, −16.84 kcal.mol^−1^ for Tacedinaline, and −20.23 kcal.mol^−1^ for Zabadinostat, demonstrating a trend for stabilization from Chidamide to Zabadinostat. In water (∆G^78^), the calculated free energies are −8.69 kcal.mol^−1^ for Chidamide, −12.12 kcal.mol^−1^ for Entinostat, −15.39 kcal.mol^−1^ for o-ABA, −16.57 kcal.mol^−1^ for Mocetinostat, −17.33 kcal.mol^−1^ for Tacedinaline, and −20.39 kcal.mol^−1^ for Zabadinostat. Zabadinostat shows the most favorable stabilization in water media. The optimized geometry of the representative [o-ABA-Zn(H_2_O)_4_]^2+^ complex in water is presented in Figure 5.

#### 2.3.3. Influence of Coordination Number on Zn^2+^ Binding Thermodynamics

Zinc (II) complexes have flexible coordination geometries. In condensed media, Zn^2+^ forms hexacoordinated species such as [Zn(H_2_O)_6_]^2+^. However, previous quantum chemical calculations [52] have shown that Zn^2+^ preferentially adopts a tetrahedral geometry when bound with ligands in environments mimicking a protein binding site. Notably, the stabilization of the tetrahedral Zn ^2+^ is affected by the nature of the donor atoms and solvent exposure.

A comparison of the two coordination modes reveals the thermodynamic preference based on the coordination. In the gas phase, hexacoordinated Zn^2+^ complexes are more exergonic than tetracoordinated ones. In this case, ∆G values for hexacoordinated complexes range from −34.98 to −52.2 kcal.mol^−1^, compared with −19.85 to −26.84 kcal.mol^−1^ for the tetracoordinated species. This suggests that in the gas phase, the higher the coordination number, the stronger the complex stability is. Oppositely, in condensed media, the situation changes drastically. Our calculations reveal that in methanol (∆G^33^), tetracoordinated complexes have lower binding free energies (−34.43 to −35.91 kcal.mol^−1^) compared with hexacoordinated species (−8.78 to −20.23 kcal.mol^−1^). Similar trends were observed in water (∆G^78^), where tetracoordinated complexes have ∆G values between −36.47 and −38.19 kcal.mol^−1^, whereas hexacoordinated complexes fall in the range of −8.69 to −20.39 kcal.mol^−1^. Thus, under physiologically relevant conditions, the tetracoordinated binding mode is thermodynamically preferred, revealing zinc coordination behavior within the HDAC active site.

#### 2.3.4. Relative Zinc-Binding Affinity of BBHDACi

The calculated substitution free energies are presented in Table 7 and reveal a contrast between the metal-binding preferences and thermodynamic outcomes in the gas phase and in condensed media. In this context, the o-ABA scaffold serves as a minimal representation of the conserved o-aminoanilide zinc-binding group present in BBHDACi.

In the gas phase (ΔG^1^), all studied inhibitors have negative substitution free energies relative to the o-ABA reference scaffold, with ∆G values of −6.99 kcal.mol^−1^ for Tacedinaline, −5.45 kcal.mol^−1^ for Zabadinostat, −4.67 kcal.mol^−1^ for Mocetinostat, −2.29 kcal.mol^−1^ for Entinostat, and −1.45 kcal.mol^−1^ for Chidamide. These negative values indicate that the substitution of the reference [o-ABA-Zn(H_2_O)_2_]^2+^ complex by the studied benzamide-based inhibitors is thermodynamically favorable in the gas phase.

However, upon inclusion of solvation effects, these results differ. In methanol (ΔG^33^), the calculated ∆G values are 0.89 kcal.mol^−1^ for Chidamide, −0.71 kcal.mol^−1^ for Tacedinaline, −0.25 kcal.mol^−1^ for Zabadinostat, −0.17 kcal.mol^−1^ for Entinostat, and 0.59 kcal.mol^−1^ for Mocetinostat.

In water (ΔG^78^), the substitution free energies are −0.44 kcal.mol^−1^ for Tacedinaline, −0.13 kcal.mol^−1^ for Chidamide, 0.02 kcal.mol^−1^ for Entinostat, 0.98 kcal.mol^−1^ for Mocetinostat, and 1.28 kcal.mol^−1^ for Zabadinostat.

Thus, in condensed media, the free energy differences vary within a narrow range of approximately −0.71 to 1.28 kcal.mol^−1^ (within ±2 kcal.mol^−1^) and are within the expected uncertainty of the computational approach and the chosen computational protocol. Consequently, under solvated conditions, which are close to the physiologically relevant conditions, none of the studied inhibitors exhibits clearly dominant zinc-binding affinity compared with the reference scaffold. Moreover, similar ΔG values in methanol and water suggest that, while a big difference in intrinsic affinity is observed in the gas phase, these differences are smaller in condensed media. Taken together, these results shed more light on the binding mechanism of BBHDACi to the Zn^2+^ ion in the enzyme active site. First, the BBHDACi possesses a strong ability to coordinate to the Zn^2+^ ion; direct metal binding cannot explain the increased affinity and selectivity that are observed experimentally. Second, calculated results suggest that metal binding does not determine their total binding affinity. These properties seem to be governed by the linker and cap region and their interaction within the HDAC active site. Moreover, noncovalent interactions, such as hydrogen bonding, hydrophobic contacts, and π–π stacking are crucial for the stabilization of the enzyme–inhibitor complex. Note that the simplified substitution model used in this study does not account for these interactions; it focuses on estimating the influence of the linker and cap regions on the ZBG and metal coordination.

The present study has several limitations. First, a simplified Zn^2+^ coordination model was employed, in which the Zn^2+^ ion is not coordinated by amino acid residues from the first coordination sphere, and therefore the influence of the protein environment is not considered. Second, the analysis focuses on the thermodynamic aspects of metal–ligand binding and the contribution of the zinc-binding group within the pharmacophore model, without addressing detailed binding modes within the enzyme through molecular docking approaches. Finally, comparison with crystallographic data provides validation of key structural parameters; however, the simplified model may lead to deviations in Zn-O bond distances, highlighting the need for future studies incorporating active-site models.

## 3. Materials and Methods

### 3.1. Computational Methodology

All quantum-chemical calculations were performed using the Gaussian 16 software package [53]. Molecular structures and optimized geometries were visualized using ChemCraft version 1.8.3 [54]. Initially, all studied inhibitors were fully optimized in the gas phase at the B3LYP/6-311++g(d,p) level of theory. The absence of imaginary frequencies, after frequency calculations, revealed that all optimized geometries correspond to real minima on the potential energy surface. The Cartesian coordinates of all optimized structures are provided in the Supplementary Information. Thus, the optimized geometries serve as a reliable starting point for studying coordination preferences and metal-binding interactions.

The combination of the B3LYP functional and Pople’s basis set of high quality was chosen based on previous studies of hydroxamic acids [55,56] and hydroxamic acid derivatives [57] and our own validation in previous studies [57]. The applied computational method accurately reproduced the geometries of metal ions and enzyme complexes, demonstrating strong agreement between theoretical predictions and experimental results [57]. Results are presented in Table 8 (adapted from our previous study) [57].

The Polarizable Continuum Model (PCM) computations in methanol (ε = 33) and water (ε = 78) were performed on all studied inhibitors to account for solvation effects. It should be noted that metalloprotein binding sites are located within cavities, where the dielectric properties mimic low-polarity solvents [48].

In this study, o-ABA, Chidamide, Entinostat, Tacedinaline, Mocetinostat and Zabadinostat were explicitly modeled. The o-ABA molecule served as a reference model for the conserved o-aminoanilide zinc binding group present in the BBHDACi. This simplified model keeps the crucial coordination fragment responsible for Zn^2+^ binding in HDACs. Consequently, the zinc-water complex [o-ABA-Zn(H_2_O)_2_]^2+^ served as the initial model for substitution reactions. In complexes with organic and protein ligands, Zn^2+^ tries to reduce its coordination number from six to four. Metal–inhibitor complexes were modeled in two coordination modes: as tetracoordinated complexes, [BBHDACi-Zn(H_2_O)_2_]^2+^, and as hexacoordinated complexes, [BBHDACi-Zn(H_2_O)_4_]^2+^.

In each model, the inhibitor corresponded to one of the studied BBHDACi: o-ABA, Chidamide, Entinostat, Tacedinaline, Mocetinostat, and Zabadinostat. The optimized geometries of all studied inhibitors were obtained at the B3LYP/6-311++g(d,p) level of theory in the gas phase, methanol, and water. Thus, the optimized geometries were subsequently used for modeling Zn^2+^ coordination and evaluating zinc-ligand interactions.

### 3.2. Modeling of Metal–Inhibitor Complexes and Substitution Reactions

The initial complex formation between each benzamide-based inhibitor and the hydrated Zn^2+^ ion was modeled to mimic the coordination environment within the HDAC active site, and the corresponding Gibbs free energies were calculated. In these calculations, the inhibitor was studied in two possible coordination modes (Equation (1)) and (Equation (2)) to determine the preferred binding geometry. In this model, BBHDACi replaces coordinated water molecules from the [Zn(H_2_O)_6_]^2+^ complex. The general reactions describing the formation of the metal–inhibitor complexes are presented below:

Tetracoordinated model:

BBHDACi + [Zn(H_2_O)_6_]^2+^ → [BBHDACi-Zn(H_2_O)_2_]^2+^ + 4H_2_O (1)

Hexacoordinated model:

BBHDACi + [Zn(H_2_O)_6_]^2+^ → [BBHDACi-Zn(H_2_O)_4_]^2+^ + 2H_2_O (2)

The thermodynamic data obtained from these metal–inhibitor complex formation reactions were subsequently used as the basis for substitution modeling and comparative thermodynamic evaluation.

To evaluate the metal-binding affinities of the studied BBHDACi, a series of substitution reactions were modeled. The goal was to assess the competitive binding of the selected BBHDACi relative to the reference o-ABA scaffold. Note that the aim of this study is to evaluate reliable trends rather than to reproduce their absolute values.

In the modeled substitution reactions, the reference complex [o-ABA-Zn(H_2_O)_2_]^2+^ was replaced by a competing BBHDACi, enabling comparative evaluation of their relative Zn^2+^ binding affinity in different media.

Two coordination geometries of Zn^2+^ were examined, corresponding to tetracoordinated and hexacoordinated models. The relative Gibbs free energies of the substitution reactions were calculated in the gas phase, methanol, and water.

Tetracoordinated model:

BBHDACi + [o-ABA-Zn(H_2_O)_2_]^2+^ → [BBHDACi-Zn(H_2_O)_2_]^2+^ + o-ABA (3)

Hexacoordinated model:

BBHDACi + [o-ABA-Zn(H_2_O)_4_]^2+^ → [BBHDACi-Zn(H_2_O)_4_]^2+^ + o-ABA (4)

The approach used allows the following: (1) comparison of the inhibitors’ thermodynamic preference for zinc coordination among the studied inhibitors in consistent coordination environments, (2) systematic evaluation of the relative metal-binding affinity of the BBHDACi series.

Free energies in solutions were calculated by adding solvation contributions to the gas-phase Gibbs free energies. The solvation energy (∆G^ε^_solv_) was calculated as the difference between gas-phase energies and the corresponding PCM energies for each inhibitor studied, according to the relation:

∆G^ε^_solv_ ≈ G_el_^2^ − G_el_^1^ (5)

To assess the relative metal-binding affinity of the BBHDACi, the [o-ABA-Zn(H_2_O)_2_]^2+^ was taken as a reference. The gas-phase free energy change associated with a BBHDACi substitution was calculated using the following equation:

∆G^1^ = G^1^([o-ABA]) + G^1^([BBHDACi-Zn(H_2_O)**_2_**]^2+^) − G^1^([BBHDACi]) − G^1^([o-ABA-Zn(H_2_O)_2_]^2+^) (6)

For the condensed phase, the overall free energy change was obtained as:

∆G^x^ = ∆G^1^ + ∆G^ε^_solv_ (products) − ∆G^ε^_solv_ (reagents), (7)

and

∆G^1^ = ∆H^1^ − T∆S^1^ (8)

where H and S are enthalpy and entropy, respectively.

A positive ∆G^x^ indicates lower affinity of the competing inhibitor for the Zn^2+^ ion relative to o-ABA, whereas a negative value of ∆G^x^ corresponds to thermodynamically favorable substitution, implying that the competitive inhibitor exhibits a stronger zinc coordination compared with the o-ABA scaffold.

## 4. Conclusions

The present DFT investigation provides a thermodynamic and structural framework for understanding the factors governing Zn^2+^ coordination in clinically relevant BBHDACi.

First, geometry optimization confirmed that the o-aminoanilide scaffold ensures appropriate donor atom orientation for stable bidentate Zn^2+^ coordination across the series. The trans (E) isomer of Chidamide was identified as thermodynamically preferred in polar media (∆G = −3.34 to −4.48 kcal.mol^−1^), supporting its relevance as the bioactive isomer. Geometry optimization of all other molecules revealed specific structural features.

Second, all studied BBHDACi exhibited favorable Zn^2+^ binding. In polar media, comparative analysis of tetracoordinated and hexacoordinated complexes demonstrated that tetracoordinated complexes were significantly more stable. Bond length analysis (Zn-O^1^ ≈ 2.00–2.07 Å, and Zn-N^1^ ≈ 2.08–2.11 Å) additionally supported bidentate chelation across the inhibitor series.

Notably, the relative substitution free energies in condensed media differed by less than ±2 kcal.mol^−1^, indicating nearly identical intrinsic Zn^2+^ affinities among the studied molecules. These findings suggest that direct metal coordination alone does not fully determine differences in inhibitor potency and selectivity. Structural features such as linker length, cap region composition, and heteroatom distribution exert minimal influence on direct metal coordination and are likely to govern overall binding efficiency through additional non-covalent interactions within the HDAC catalytic pocket.

Finally, this work provides molecular insight into the zinc binding behavior of BBHDACi and establishes a quantitative thermodynamic basis for the rational design of next-generation isoform-selective BBHDACi.

## Figures and Tables

**Table 1:** Overview of the clinically relevant BBHDACi included in this study.

Compound	Development Code (Trade Name)	HDAC Isoform Selectivity	Therapeutic IMPLICATIONS	Clinical Status (Ref.)
Entinostat	MS-275	Class I	Breast cancer and hematological malignancies	Phase III clinical trials [34,35].
Mocetinostat	MGCD0103	Class I and Class IV	Hematological and solid tumors	Phase I and Phase II clinical trials [36].
Tacedinaline	Cl-994	Class I	Various malignancies	Early-phase clinical studies [37].
Zabadinostat	CXD101	Class I	Peripheral T-cell lymphoma (PTCL)	Clinical trials [38,39].
Chidamide	Epidaza	Class I and Class IIb	Peripheral T-cell lymphoma (PTCL)	Approved in China and Japan [40,41].

**Table 2:** Relative Gibbs free energies (∆G, kcal.mol^−1^) of the cis (Z) and trans (E) isomers of Chidamide optimized at the B3LYP/6-311++g(d,p) level of theory in the gas phase (∆G^1^), methanol (∆G^33^), and water (∆G^78^).

Isomer	ΔG^1^	ΔG^33^	ΔG^78^
1Z	0.0	0.0	0.0
1E	−3.34	−4.48	−3.86

**Table 3:** Relative solvation Gibbs free energies (∆G, kcal.mol^−1^) of o-aminobenzanilide and BBHDACi at the B3LYP/6-311++g(d,p) level of theory in the gas phase (∆G^1^), methanol (∆G^33^), and water (∆G^78^), referenced to the gas-phase free energy (∆G^1^).

BBHDACi	ΔG^1^	ΔG^33^	ΔG^78^
o-ABA	0.0	−8.74	−9.87
Entinostat	0.0	−15.48	−16.14
Mocetinostat	0.0	−13.48	−13.95
Tacedinaline	0.0	−13.51	−13.96
Zabadinostat	0.0	−12.41	−13.36

**Table 4:** Calculated Gibbs free energy changes (∆G, kcal.mol^−1^) for the formation of o-ABA, Chidamide, Entinostat, Mocetinostat, Zabadinostat and Tacedinaline tetracoordinated complexes with Zn^2+^, obtained using B3LYP/6-311++g(d,p) level of theory in the gas phase (∆G^1^), methanol (∆G^33^), and water (∆G^78^).

Reaction Scheme	ΔG^1^	ΔG^33^	ΔG^78^
Entinostat + [Zn(H_2_O)_6_]^2+^ → [Entinostat-Zn(H_2_O)_2_]^2+^ + 4H_2_O	−22.14	−35.19	−37.73
Mocetinostat + [Zn(H_2_O)_6_]^2+^ → [Mocetinostat-Zn(H_2_O)_2_]^2+^ + 4H_2_O	−24.52	−34.43	−36.77
Zabadinostat + [Zn(H_2_O)_6_]^2+^ → [Zabadinostat-Zn(H_2_O)_2_]^2+^ + 4H_2_O	−25.30	−35.27	−36.47
Tacedinaline + [Zn(H_2_O)_6_]^2+^ → [Tacedinaline-Zn(H_2_O)_2_]^2+^ + 4H_2_O	−26.84	−35.73	−38.19
Chidamide + [Zn(H_2_O)_6_]^2+^ → [Chidamide-Zn(H_2_O)_2_]^2+^ + 4H_2_O	−21.30	−35.91	−37.61
o-ABA + [Zn(H_2_O)_6_]^2+^ → [o-ABA-Zn(H_2_O)_2_]^2+^ + 4H_2_O	−19.85	−35.02	−37.75

**Table 5:** Calculated Zn^2+^-BBHDACi bond distances in (Å) of tetracoordinated [BBHDACi-Zn(H_2_O)_2_]^2+^ complexes optimized at the B3LYP/6-311++g(d,p) level of theory in methanol (∆G^33^), and water (∆G^78^).

	[Entinostat-Zn(H_2_O)_2_]^2+^		[Mocetinostat-Zn(H_2_O)_2_]^2+^		[Tacedinaline-Zn(H_2_O)_2_]^2+^		[Zabadinostat-Zn(H_2_O)_2_]^2+^		[Chidamide-Zn(H_2_O)_2_]^2+^		[OAB-Zn(H_2_O)_2_]^2+^	
Bond/Media	W	Me	W	Me	W	Me	W	Me	W	Me	W	Me
Zn-O^1^	2.02	2.01	2.00	2.00	2.00	2.03	2.02	2.01	2.07	2.06	2.04	2.01
Zn-N^1^	2.10	2.10	2.11	2.11	2.09	2.08	2.09	2.09	2.10	2.10	2.08	2.10
Zn-W^1^	2.08	2.07	2.11	2.07	2.07	2.08	2.08	2.08	2.10	2.06	2.08	2.08
Zn-W^2^	2.11	2.07	2.05	2.07	2.08	2.08	2.08	2.07	2.06	2.09	2.05	2.07

**Table 6:** Calculated Gibbs free energy changes (ΔG, kcal.mol^−1^) for the formation of hexacoordinated Zn^2+^ complexes of o-ABA, Chidamide, Entinostat, Mocetinostat, Tacedinaline, and Zabadinostat, obtained using the B3LYP/6-311++g(d,p) level of theory in the gas phase (∆G^1^), methanol (∆G^33^), and water (∆G^78^).

Reaction Scheme	ΔG^1^	ΔG^33^	ΔG^78^
Entinostat + [Zn(H_2_O)_6_]^2+^ → [Entinostat-Zn(H_2_O)_4_]^2+^ + 2H_2_O	−52.2	−12.65	−12.12
Mocetinostat + [Zn(H_2_O)_6_]^2+^ → [Mocetinostat-Zn(H_2_O)_4_]^2+^ + 2H_2_O	−37.8	−15.72	−16.57
Zabadinostat + [Zn(H_2_O)_6_]^2+^ → [Zabadinostat-Zn(H_2_O)_4_]^2+^ + 2H_2_O	−44.79	−20.23	−20.39
Tacedinaline + [Zn(H_2_O)_6_]^2+^ → [Tacedinaline-Zn(H_2_O)_4_]^2+^ + 2H_2_O	−39.11	−16.84	−17.33
Chidamide + [Zn(H_2_O)_6_]^2+^ → [Chidamide-Zn(H_2_O)_4_]^2+^ + 2H_2_O	−39.55	−8.78	−8.69
o-ABA + [Zn(H_2_O)_6_]^2+^ → [o-ABA-Zn(H_2_O)_4_]^2+^ + 2H_2_O	−34.98	−15.60	−15.39

**Table 7:** Calculated Relative Gibbs Free Energies (ΔG, kcal.mol^−1^) for substitution reactions of BBHDACi with the reference [o-ABA-Zn(H_2_O)_2_]^2+^ complex in the gas phase (∆G^1^), methanol (∆G^33^), and water (∆G^78^).

**Reaction Scheme**	**ΔG^1^**	**ΔG^33^**	**ΔG^78^**
Entinostat + [o-ABA-Zn(H_2_O)_2_]^2+^ → [Entinostat-Zn(H_2_O)_2_]^2+^ + o-ABA	−2.29	−0.17	0.02
Mocetinostat + [o-ABA-Zn(H_2_O)_2_]^2+^ → [Mocetinostat-Zn(H_2_O)_2_]^2+^ + o-ABA	−4.67	0.59	0.98
Zabadinostat + [o-ABA-Zn(H_2_O)_2_]^2+^ → [Zabadinostat-Zn(H_2_O)_2_]^2+^ + o-ABA	−5.45	−0.25	1.28
Tacedinaline + [o-ABA-Zn(H_2_O)_2_]^2+^ → [Tacedinaline-Zn(H_2_O)_2_]^2+^ + o-ABA	−6.99	−0.71	−0.44
Chidamide + [o-ABA-Zn(H_2_O)_2_]^2+^ → [Chidamide-Zn(H_2_O)_2_]^2+^ + o-ABA	−1.45	−0.89	−0.13

**Table 8:** Comparison of computed and experimental mean bond distances in Zn−ligand complexes (in Å), adapted from [57].

Complex	Bond Type	Calculated	Experimental	Ref.
[Zn(H_2_O)_6_]^2+^	Zn-O	2.13	2.08 ± 0.03	[57,58]
[Zn(H_2_O)_n_(imidazole)_1_]^2+^	Zn-N	1.97	2.00 ± 0.02	[57,59]

## Data Availability

The data presented in this study are available on request from the corresponding authors.

## Supplementary Materials

The following supporting information can be downloaded at: https://www.mdpi.com/article/10.3390/molecules31101650/s1, (S1) the optimized geometries of the studied molecules (Figures S1–S5), corresponding to the final computed structures used in the study, (S2) Cartesian coordinates of all optimized structures.

## Abbreviations

The following abbreviations are used in this manuscript:

HDACs	Histone Deacetylase(s)
HATs	Histone Acetyl Transferases
HDACi	Histone Deacetylase Inhibitor
PTM (s)	Post-Translational Modification(s)
DFT	Density Functional Theory
B3LYP	Becke, 3-parameter, Lee-Yang-Parr hybrid functional
PCM	Polarizable Continuum Model
ΔG^1^	Change in Gibbs Free Energy (gas phase)
ΔG^33^	Change in Gibbs Free Energy in Methanol (ε = 33)
ΔG^78^	Change in Gibbs Free Energy in Water (ε = 78)
BBHDACi	Benzamide-Based Histone Deacetylase Inhibitor
PTCL	Peripheral T-cell Lymphoma
ZBG	Zinc-binding group
o-ABA	ortho-aminobenzanilide
o-AA	ortho-aminoanilide
MBPs	Metal-binding pharmacophores
