# Supplementary material for: Zinc Affinity of Benzamide-Based Histone Deacetylase Inhibitors: A DFT Study
Source: Molecules. 2026 May 14;31(10):1650. doi: 10.3390/molecules31101650 (PMC13210232; doi:10.3390/molecules31101650)
Supplement: Supplementary file 1 [file molecules-31-01650-s001.zip › molecules-4262598-supplementary.pdf]

Supplementary Information

Zinc Affinity of Benzamide-Based Histone Deacetylase Inhibitors: A DFT Study

Nikolay Toshev[1,*], Kristiyan Velichkov[2], Yordanka Uzunova[1,3], Diana Cheshmedzhieva[4] and Todor Dudev[4]

1 Department of Bioorganic Chemistry, Faculty of Pharmacy, Medical University of Plovdiv, 15A Vassil Aprilov Blvd., 4002 Plovdiv, Bulgaria
2 Medical Faculty, Medical University of Plovdiv, 15A Vassil Aprilov Blvd., 4002 Plovdiv, Bulgaria
3 Research Institute, Medical University of Plovdiv, 15A Vassil Aprilov Blvd, 4002 Plovdiv, Bulgaria
4 Department of Pharmaceutical and Applied Organic Chemistry, Faculty of Chemistry and Pharmacy, Sofia University "St. Kliment Ohridski", 1 James Bourchier Blvd., 1164 Sofia, Bulgaria
* Correspondence: nikolay.toshev@mu-plovdiv.bg;

Table of Contents

S1. Optimized geometries of the studied benzamide-based HDACi (Figure S1- Figure S5)

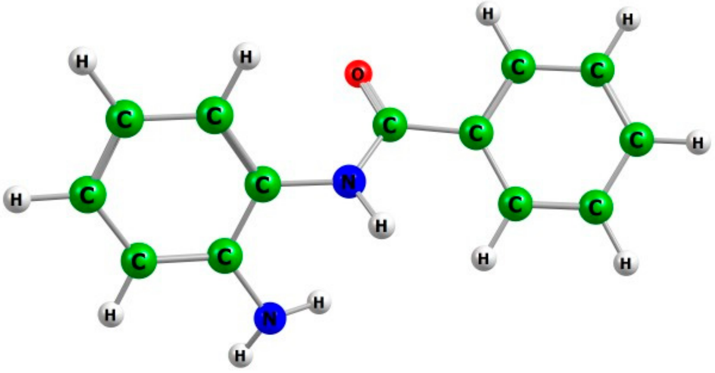

Figure S1. B3LYP/6-311++g(d,p) optimized structure of o-ABA in water.

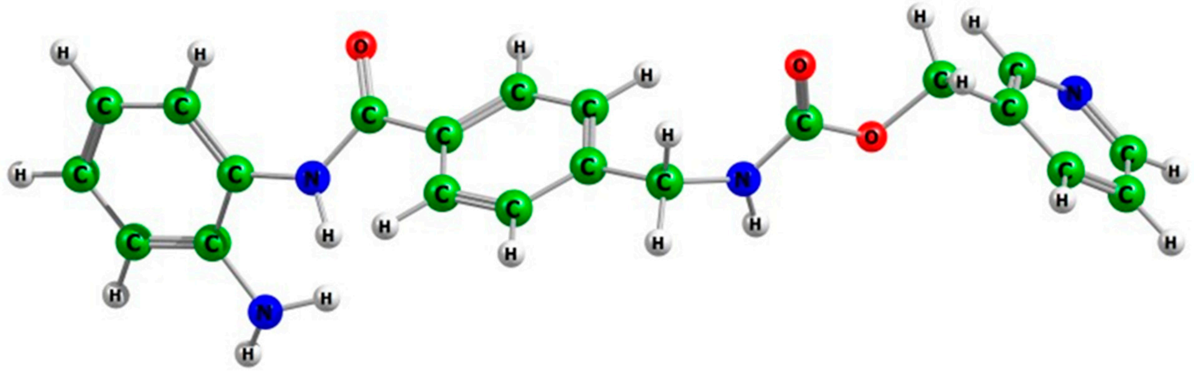

Figure S2. B3LYP/6-311++g(d,p) optimized structure of Entinostat in water.

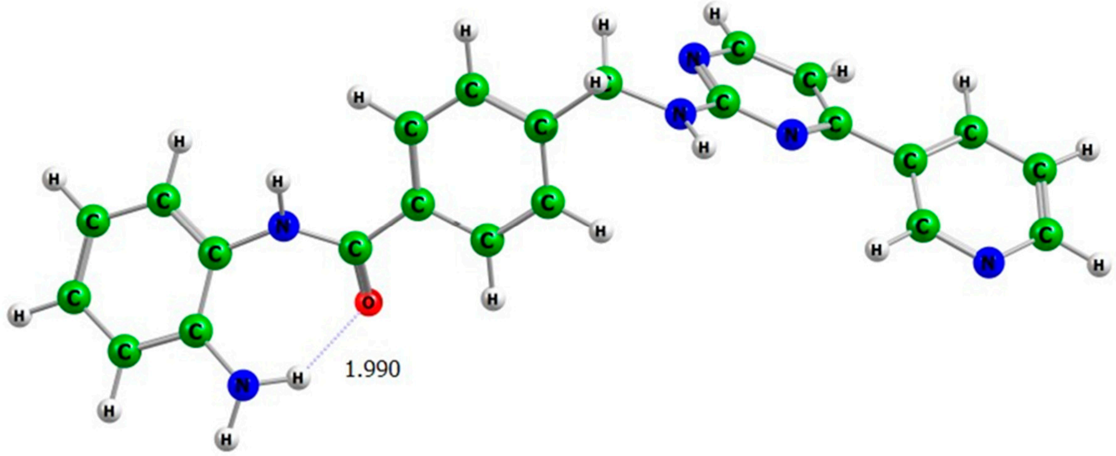

Figure S3. B3LYP/6-311++g(d,p) optimized structure of Mocetinostat in water

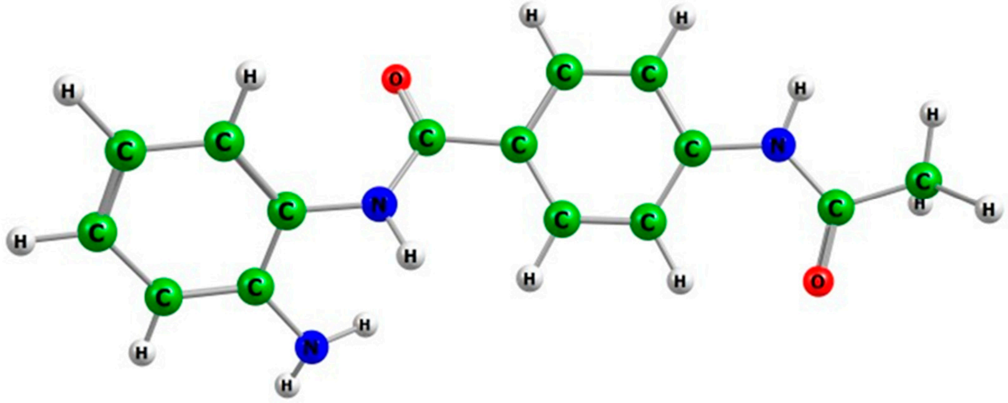

Figure S4. B3LYP/6-311++g(d,p) optimized structure of Tacedinaline in water.

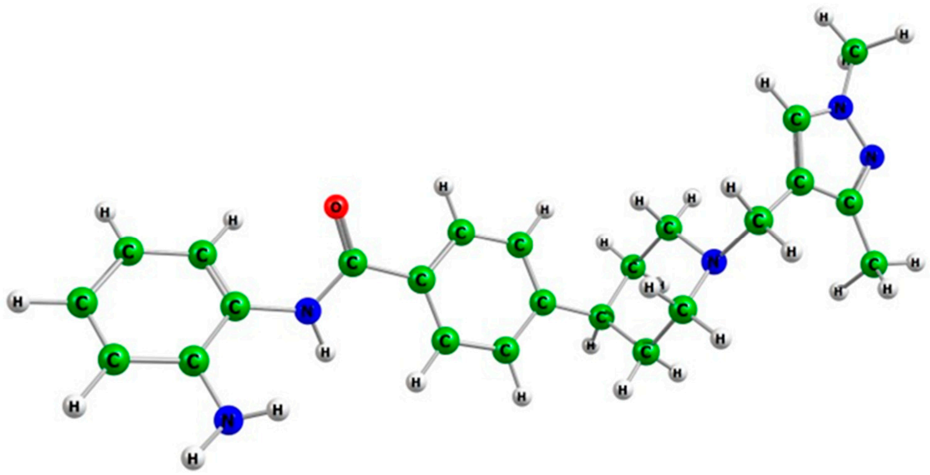

Figure S5. B3LYP/6-311++g(d,p) optimized structure of Zabadinostat in water.

S2. Cartesian coordinates of all studied inhibitors and their metal complexes.

Cartesian coordinates of optimized geometries.

Entinostat in gas phase

C	8.58876300	-1.28985500	-0.15618300
C	7.97131700	-0.75536900	-1.28365100
C	6.62809200	-0.38745300	-1.24626200
C	5.89138500	-0.55550300	-0.07047400
C	6.49955400	-1.14076000	1.06145600
C	7.84844600	-1.48791800	1.00738500
N	5.72210400	-1.29353600	2.24217100
N	4.55843400	-0.12487500	0.08990900
C	3.64170900	0.20375400	-0.88028200
O	3.84228700	0.05623300	-2.07624900
C	2.33473800	0.74575000	-0.36655800
C	1.21631900	0.62662100	-1.20176500
C	-0.02111300	1.11043200	-0.80115300
C	-0.17483600	1.74452100	0.43802100
C	0.94333600	1.87847200	1.26200500
C	2.18644200	1.38599400	0.86738800
C	-1.51648700	2.30865100	0.86401400
N	-2.60329000	1.34724200	0.75722700
C	-3.72861400	1.58306800	0.02957600
O	-3.95833700	2.57732100	-0.62696200
O	-4.57995600	0.52509500	0.13993400
C	-5.81067400	0.63657200	-0.61863100
C	-6.66234800	-0.55479800	-0.28841500
C	-6.70166700	-1.66533500	-1.13559900

N	-7.42641900	-2.76216900	-0.90175400
C	-8.15488300	-2.78971600	0.21737400
C	-8.19386800	-1.73913100	1.13353000
C	-7.43531500	-0.60403300	0.87309500
H	9.63552300	-1.56933600	-0.18276100
H	8.53360500	-0.61608000	-2.19950200
H	6.14292600	0.03183200	-2.11470400
H	8.31489200	-1.93079500	1.88197400
H	4.94381400	-1.93496900	2.12293500
H	6.27707400	-1.59819400	3.03176800
H	4.27485200	-0.03481800	1.05591300
H	1.34299800	0.15455900	-2.16817600
H	-0.87845600	1.00178000	-1.45632000
H	0.84830300	2.38021000	2.21969600
H	3.03706000	1.53825000	1.52291700
H	-1.79410000	3.15241000	0.22817100
H	-1.44635600	2.68524300	1.89001200
H	-2.54406400	0.46859500	1.24882000
H	-5.56902800	0.67132200	-1.68285400
H	-6.30396800	1.57322600	-0.35240800
H	-6.11850500	-1.66504900	-2.05362000
H	-8.73651100	-3.69120100	0.38823200
H	-8.80783900	-1.81218600	2.02342900
H	-7.44173000	0.23560700	1.56058200

Cartesian coordinates of optimized geometries.

Entinostat in water

C	8.60312700	-1.20837300	-0.25830100
C	8.02202000	-0.40908900	-1.24387900
C	6.69366800	-0.01220100	-1.11151500
C	5.93794500	-0.41985000	-0.01113900
C	6.51137700	-1.24862600	0.97699200
C	7.85559100	-1.62188400	0.83972500
N	5.76203700	-1.61876500	2.10041700
N	4.60231400	0.01843000	0.18594800
C	3.62190900	0.10458100	-0.76587500
O	3.78032900	-0.27071300	-1.92586100
C	2.31674000	0.67844700	-0.29787100
C	1.14847600	0.28449900	-0.96194300
C	-0.08896000	0.78645700	-0.57731200
C	-0.19104800	1.70956500	0.46933200
C	0.97637600	2.11140000	1.12360000
C	2.21780300	1.60399200	0.74768600
C	-1.52495600	2.31446300	0.87041000
N	-2.64242500	1.38826700	0.79189800
C	-3.69578100	1.56140900	-0.04128300
O	-3.82795200	2.46552300	-0.85095600
O	-4.60803400	0.57131000	0.14143300
C	-5.79035700	0.63104500	-0.70161100
C	-6.66993300	-0.52769400	-0.32872900
C	-6.61723300	-1.72356700	-1.04917100
N	-7.35904200	-2.80044000	-0.76664300
C	-8.19905200	-2.71538800	0.27274400

C	-8.33452200	-1.57032400	1.05530000
C	-7.55744300	-0.45895000	0.74708100
H	9.63820500	-1.51842800	-0.34643800
H	8.59840500	-0.08750100	-2.10302300
H	6.23456300	0.62212300	-1.85777700
H	8.30528600	-2.25386400	1.59844800
H	4.79683500	-1.85858600	1.90953100
H	6.19807900	-2.33285500	2.66862400
H	4.34250000	0.22924500	1.13995100
H	1.22092000	-0.42319500	-1.77851500
H	-0.98317000	0.45576200	-1.09271400
H	0.91919300	2.83082300	1.93361700
H	3.10351800	1.95800500	1.26271800
H	-1.76338400	3.15140700	0.21048100
H	-1.45264900	2.71486700	1.88509000
H	-2.67643100	0.60447100	1.42720800
H	-5.48276900	0.57633900	-1.74676200
H	-6.29449900	1.58370100	-0.53340100
H	-5.94551300	-1.81184600	-1.89923800
H	-8.79229200	-3.59930000	0.48671300
H	-9.03541100	-1.55345200	1.88084000
H	-7.64080300	0.45142000	1.33093200

Cartesian coordinates of optimized geometries.

Entinostat in methanol

C	8.60913300	-1.20205500	-0.25317800
C	8.02660100	-0.40726200	-1.24131800
C	6.69616000	-0.01590400	-1.11337600
C	5.93973100	-0.42475000	-0.01388600
C	6.51445800	-1.25020500	0.97624700
C	7.86036800	-1.61757100	0.84338000
N	5.76226200	-1.62244000	2.09814600
N	4.60287300	0.00891400	0.18229200
C	3.62619900	0.11040900	-0.77231500
O	3.78842500	-0.24759900	-1.93684300
C	2.31993000	0.67910000	-0.30045900
C	1.15408300	0.29892200	-0.97679900
C	-0.08400600	0.79757200	-0.59030200
C	-0.18928000	1.70375000	0.47082800
C	0.97565400	2.09187200	1.13750400
C	2.21783300	1.58766700	0.75949500
C	-1.52398900	2.30520300	0.87386100
N	-2.63983000	1.37742400	0.78926200
C	-3.70023600	1.56202000	-0.03291600
O	-3.83972200	2.47774900	-0.82773300
O	-4.60994700	0.56808500	0.14256100
C	-5.79759500	0.63769000	-0.69211500
C	-6.67579100	-0.52428600	-0.32621000
C	-6.62734500	-1.71316100	-1.05848100
N	-7.36820200	-2.79235000	-0.78282900
C	-8.20296000	-2.71705000	0.26140600
C	-8.33403600	-1.57961400	1.05578200

C	-7.55792400	-0.46566200	0.75458800
H	9.64582500	-1.50748000	-0.33830100
H	8.60350700	-0.08481000	-2.09979700
H	6.23619300	0.61450300	-1.86225600
H	8.31071800	-2.24675400	1.60407400
H	4.80282100	-1.87980300	1.90047000
H	6.20460800	-2.32783700	2.67240000
H	4.33946500	0.20591100	1.13822600
H	1.22945100	-0.39483200	-1.80498100
H	-0.97642800	0.47826300	-1.11592800
H	0.91607300	2.79841900	1.95864600
H	3.10177100	1.93160100	1.28434800
H	-1.76358500	3.14459700	0.21748700
H	-1.45278900	2.70119900	1.89045400
H	-2.66869200	0.58484800	1.41380200
H	-5.49661200	0.59420300	-1.73975800
H	-6.29979400	1.58895100	-0.51072000
H	-5.95992000	-1.79354600	-1.91272100
H	-8.79554900	-3.60279800	0.46957200
H	-9.03087700	-1.57047800	1.88488900
H	-7.63789700	0.43903100	1.34770400

Cartesian coordinates of optimized geometries.

[Entinostat -Zn(H_2O)$_2$]$^{2+}$ in gas phase

C	-4.44702500	3.95501300	-0.24151100
C	-3.41861900	3.06905700	0.05658400
C	-3.69360200	1.76058400	0.46909100
C	-5.03248500	1.36399200	0.59624100
C	-6.06455100	2.24747800	0.28353400
C	-5.77420400	3.54366000	-0.13165900
H	-4.21143800	4.96365500	-0.55728800
H	-2.38566500	3.38671300	-0.03210500
H	-7.09807800	1.93523700	0.39854600
H	-6.58201500	4.23081300	-0.35095900
N	-5.36652200	-0.00132900	1.01209900
H	-6.34185500	-0.04128200	1.30672800
N	-2.58535300	0.93004900	0.84033900
C	-2.17885100	-0.23832800	0.28028200
O	-2.93467800	-0.86504200	-0.53800300
H	-1.86135600	1.42280000	1.34857300
Zn	-4.82383500	-1.14232600	-0.60500300
H	-4.81190200	-0.27100500	1.82744800
C	-0.86071300	-0.75958900	0.60901900
C	-0.29847900	-1.73950300	-0.23146700
C	-0.10413800	-0.28983500	1.70443200
C	0.98630100	-2.20050100	-0.00408700
H	-0.85684400	-2.09281900	-1.08833200
C	1.17217400	-0.76885100	1.92992100
H	-0.51340200	0.42253200	2.41385700
C	1.74497700	-1.71711500	1.06919300
H	1.42348400	-2.91970000	-0.68707000
H	1.74947000	-0.39852100	2.76806200

C	3.14766500	-2.24165300	1.29949200
H	3.51798900	-2.67352400	0.36366200
H	3.10019800	-3.05347000	2.03264500
C	4.41058400	-0.18404200	1.00541100
H	4.73967300	-1.52103400	2.50981000
N	4.05953800	-1.23931700	1.81897600
O	3.79144600	0.11465300	0.00356100
O	5.47828900	0.45864400	1.49866000
C	6.01818700	1.56473700	0.69974000
H	6.48896100	2.20646500	1.44260400
H	5.18462700	2.08857000	0.23386400
C	7.00865700	1.08152300	-0.32127900
C	8.34929300	0.86474500	0.00711500
C	6.61438000	0.83004000	-1.64005500
C	9.21924900	0.40835300	-0.97574500
H	8.70694900	1.05710700	1.01364200
H	5.58080900	0.99045000	-1.93353200
C	8.71587700	0.18454000	-2.25719900
H	10.26685900	0.23504200	-0.76168800
H	9.36795700	-0.16967500	-3.04996300
N	7.43857100	0.39047800	-2.59268000
O	-5.80353500	-0.57758200	-2.29690100
H	-5.87647000	0.33995500	-2.59613700
H	-5.95618400	-1.15092700	-3.06158800
O	-5.10263600	-3.15941500	-0.76172700
H	-4.35251500	-3.77102500	-0.79590200
H	-5.90700800	-3.68164000	-0.63295500

Cartesian coordinates of optimized geometries.

$[Entinostat - Zn(H_2O)_2]^{2+}$ in water

C	4.99523000	3.90102900	0.85678200
C	3.86681000	3.16948000	0.51136100
C	3.98496400	1.92646200	-0.11994900
C	5.25902600	1.43540400	-0.43244700
C	6.38983600	2.16800100	-0.07085400
C	6.26249900	3.39506200	0.56853000
H	4.88488700	4.85645800	1.35425300
H	2.87731900	3.54936400	0.73686700
H	7.37255900	1.77320200	-0.30278200
H	7.15064800	3.95419300	0.83571600
N	5.42481000	0.14085000	-1.05172000
H	6.36348900	0.04927900	-1.43503600
N	2.77719300	1.26568300	-0.49699800
C	2.37298400	0.00456500	-0.23725400
O	3.08772300	-0.83642700	0.35981200
H	2.05574300	1.89091200	-0.83071000
Zn	5.03595700	-1.39756400	0.32416500
H	4.78057100	0.01169400	-1.83170300
C	1.00077300	-0.36650900	-0.66799100
C	0.39615700	-1.47751700	-0.06835100
C	0.28999000	0.35318400	-1.64157300
C	-0.90205800	-1.83946000	-0.41188700

H	0.94075200	-2.03998400	0.67871300
C	-1.00321400	-0.01813500	-1.98132200
H	0.74213500	1.18390100	-2.17058400
C	-1.62088900	-1.11204300	-1.36382800
H	-1.36437100	-2.69117000	0.07426600
H	-1.53314900	0.54873600	-2.73818500
C	-3.03730000	-1.51506800	-1.73973000
H	-3.35806200	-2.35056300	-1.11679400
H	-3.05995700	-1.85320100	-2.77797800
C	-4.52877000	-0.06599300	-0.42586100
H	-4.26314200	0.10492900	-2.43316700
N	-4.01241400	-0.43788400	-1.62111500
O	-4.30052100	-0.61473900	0.63950200
O	-5.34359700	1.01194000	-0.57529200
C	-6.04903700	1.47881700	0.60715000
H	-6.13117100	2.55531600	0.46396200
H	-5.44109200	1.27391600	1.48661400
C	-7.41051400	0.84463600	0.72207700
C	-8.49608000	1.31005800	-0.02382900
C	-7.63068200	-0.23839700	1.57794200
C	-9.72905200	0.68072800	0.10794200
H	-8.37817800	2.15587300	-0.69285600
H	-6.81181100	-0.62454700	2.17786200
C	-9.83652600	-0.39458800	0.98776800
H	-10.59330800	1.01601600	-0.45218600
H	-10.78575300	-0.90637900	1.11485300
N	-8.81164000	-0.85221200	1.71804700
O	6.27286900	-1.60952500	2.02052500
H	6.35909600	-0.85703700	2.62092900
H	6.05465900	-2.38086500	2.56116600
O	4.85160000	-3.46846100	0.37335500
H	3.96722200	-3.83098200	0.22584700
H	5.45710600	-3.97778500	-0.18279700

Cartesian coordinates of optimized geometries.

[Entinostat - $Zn(H_2O)_2]^{2+}$ in methanol

C	4.90184000	3.93979100	0.80617400
C	3.80038300	3.17845100	0.43893000
C	3.96305900	1.92240700	-0.15595700
C	5.25653100	1.44894400	-0.41012400
C	6.35992800	2.21156700	-0.02633800
C	6.18763800	3.45116100	0.57730300
H	4.75568900	4.90551300	1.27351200
H	2.79680500	3.54614000	0.61810100
H	7.35776600	1.83183300	-0.21624800
H	7.05524400	4.03387800	0.86088200
N	5.47198200	0.14818900	-1.00313200
H	6.43237900	0.06618800	-1.33049700
N	2.77742900	1.23324300	-0.55655900
C	2.37209100	-0.01945500	-0.26218800
O	3.08303700	-0.83966100	0.36948500

H	2.05927500	1.84218000	-0.92577600
Zn	5.01561500	-1.39668900	0.34238400
H	4.87820100	0.01034600	-1.82135400
C	1.00440900	-0.40899900	-0.68880800
C	0.40354900	-1.50435800	-0.05680600
C	0.28995800	0.28054000	-1.68147200
C	-0.89397100	-1.87943000	-0.38734200
H	0.94966000	-2.04228100	0.70692300
C	-1.00254000	-0.10443500	-2.00838800
H	0.73764500	1.09840900	-2.23380800
C	-1.61627400	-1.18160000	-1.35837400
H	-1.35400600	-2.71642900	0.12571400
H	-1.53640500	0.43964400	-2.77894300
C	-3.03254300	-1.59978300	-1.71777100
H	-3.35429300	-2.40471800	-1.05583900
H	-3.05299200	-1.98820000	-2.73841600
C	-4.50490900	-0.06887900	-0.47883500
H	-4.28690300	-0.04291100	-2.49974000
N	-4.00563000	-0.51682200	-1.65539500
O	-4.24621900	-0.53774400	0.61701600
O	-5.33800300	0.98480100	-0.68663300
C	-6.01870500	1.53052500	0.47658600
H	-6.12737400	2.59031000	0.25060100
H	-5.38092600	1.40810900	1.35028400
C	-7.36314600	0.88358900	0.68410200
C	-8.48043300	1.27253000	-0.05870000
C	-7.53567200	-0.13375900	1.62722000
C	-9.69663600	0.63546000	0.16179200
H	-8.39952800	2.06605200	-0.79406000
H	-6.69104400	-0.45992700	2.22690200
C	-9.75590900	-0.37025700	1.12457400
H	-10.58443000	0.91257800	-0.39334000
H	-10.69094700	-0.88570800	1.32242500
N	-8.69990000	-0.75371300	1.85309100
O	6.14709800	-1.53366200	2.06544400
H	6.21910100	-0.77371600	2.65825300
H	5.96732400	-2.31042000	2.61234600
O	4.82494500	-3.45906200	0.41467600
H	3.94127900	-3.82894500	0.28250700
H	5.43410400	-3.99496000	-0.11136000

Cartesian coordinates of optimized geometries.

[Entinostat - $Zn(H_2O)_4]^{2+}$ in gas phase

C	6.31518200	0.44080200	-1.09205700
C	5.24699400	1.32661000	-1.00446700
C	3.93540100	0.85215400	-0.96154000
C	3.68959700	-0.52754800	-1.03363700
C	4.76700000	-1.41186000	-1.10270400
C	6.07426300	-0.93119200	-1.13091500
H	7.32875900	0.81991900	-1.12439900
H	5.42500600	2.39487200	-0.95480000

H	4.58299500	-2.47945600	-1.17766400
H	6.89962500	-1.62882900	-1.20313500
N	2.33721900	-1.04243700	-1.01308700
H	2.32726700	-1.98938200	-1.38653600
N	2.85041400	1.78186100	-0.88581400
C	1.92184500	1.77205600	0.09414700
O	2.01411500	0.95618700	1.04701500
H	2.86151500	2.57203400	-1.51924000
Zn	1.30914100	-1.00551400	0.92867600
H	1.73432400	-0.48641400	-1.61894100
C	0.72273300	2.62775600	-0.02454100
C	0.07645900	3.03784100	1.14954700
C	0.07595700	2.78710500	-1.25848100
C	-1.23451200	3.48234600	1.09862500
H	0.59320000	2.96792100	2.09897200
C	-1.24861900	3.20719300	-1.29645800
H	0.56192800	2.49359900	-2.18219600
C	-1.93559500	3.48244000	-0.11160400
H	-1.74374100	3.75788800	2.01485700
H	-1.76750500	3.25208800	-2.24743800
C	-3.43494900	3.68225700	-0.11368300
H	-3.71751300	4.45517400	-0.83140400
H	-3.76544400	4.01502900	0.87429500
C	-3.97912700	1.23670400	0.01847900
H	-5.05977900	2.60551000	-0.97130500
N	-4.17117200	2.46833000	-0.50970100
O	-3.02188800	0.88023300	0.70093400
O	-4.99606600	0.41188600	-0.32908500
C	-5.09135200	-0.87258000	0.34186800
H	-6.13741800	-1.14794500	0.21794500
H	-4.88203000	-0.73556300	1.40281700
C	-4.18303900	-1.91970700	-0.25654200
C	-4.49293400	-2.59221300	-1.44164000
C	-2.97896000	-2.23927800	0.36590500
C	-3.59392100	-3.52438100	-1.95354600
H	-5.42620100	-2.39314600	-1.95745100
H	-2.71287400	-1.75865500	1.30052800
C	-2.39231000	-3.73464800	-1.28694300
H	-3.81324900	-4.07391700	-2.86021400
H	-1.65552300	-4.43177400	-1.67076000
N	-2.07925300	-3.09624600	-0.14498700
O	0.72661100	-0.65402500	2.98947500
H	0.79416200	0.26843200	3.26827400
H	-0.00364900	-1.05692500	3.47484900
O	0.40367100	-2.88550900	0.85305200
H	-0.54064000	-3.08897900	0.52234900
H	0.80895300	-3.68632800	1.20382900
O	-0.52853000	-0.31747700	-0.01291400
H	-1.06328800	-1.02321400	-0.40098700
H	-1.19172500	0.31998200	0.32691300
O	3.04523900	-1.90531500	1.88105600
H	3.09080800	-1.81220000	2.84153100
H	3.93146000	-1.72384700	1.53900800

Cartesian coordinates of optimized geometries.

[Entinostat - $Zn(H_2O)_4]^{2+}$ in water

C	6.25767700	0.45110100	-1.21689100
C	5.18795000	1.33713100	-1.14524700
C	3.88134800	0.86094400	-1.03407300
C	3.63794600	-0.52224200	-1.02441500
C	4.71855800	-1.40417800	-1.07483600
C	6.02096900	-0.92182200	-1.16812400
H	7.26826600	0.83153500	-1.29846100
H	5.35567500	2.40757800	-1.16021800
H	4.53415400	-2.47295100	-1.06501300
H	6.84652500	-1.62151400	-1.21539400
N	2.29442200	-1.03480300	-0.94695300
H	2.27787200	-2.00595600	-1.25108100
N	2.79958900	1.78884600	-0.96205300
C	1.91121300	1.80847800	0.05237800
O	2.05509900	1.04726500	1.03874800
H	2.75988800	2.52729600	-1.65327500
Zn	1.29202400	-0.94424600	1.00438300
H	1.68757700	-0.52803600	-1.59029600
C	0.68836200	2.63601600	-0.06515200
C	0.05022000	3.04197000	1.11275500
C	0.01754600	2.75415800	-1.28942200
C	-1.27498600	3.44833300	1.07792400
H	0.57524500	2.97308700	2.05720400
C	-1.31784900	3.14092900	-1.31176800
H	0.49360600	2.44507200	-2.21238100
C	-1.99334600	3.42134400	-0.12119800
H	-1.78113900	3.70543400	2.00136900
H	-1.85559400	3.14615600	-2.25330100
C	-3.49864400	3.58200800	-0.10518100
H	-3.81055100	4.36829000	-0.79444800
H	-3.82771300	3.87037200	0.89678100
C	-4.05175200	1.15780900	0.05171500
H	-4.99422500	2.47267100	-1.15199000
N	-4.20166100	2.36540200	-0.53651000
O	-3.20533100	0.86814000	0.89076100
O	-4.97257700	0.27841100	-0.41937200
C	-5.03641100	-1.02850900	0.21623600
H	-6.05733000	-1.35576900	0.02641200
H	-4.88913100	-0.91042600	1.28880700
C	-4.04385300	-2.00536200	-0.36131800
C	-4.22094100	-2.58062200	-1.62251900
C	-2.90091100	-2.35954000	0.35328700
C	-3.25960100	-3.45797600	-2.11336900
H	-5.09979100	-2.34508500	-2.21256400
H	-2.72674100	-1.94711300	1.34091700
C	-2.13521900	-3.72170200	-1.33698600
H	-3.36972100	-3.92418500	-3.08408000
H	-1.35021200	-4.37841400	-1.69550000
N	-1.95434900	-3.18085600	-0.12338600

O	0.64252400	-0.56418000	3.01167800
H	0.56663000	0.36142200	3.27689600
H	-0.14402300	-1.01873000	3.33987200
O	0.43689300	-2.83556300	1.10142100
H	-0.43328000	-3.07154600	0.64677300
H	0.99713200	-3.61928100	1.13143200
O	-0.57372800	-0.21956900	0.15936900
H	-0.95658200	-0.66038100	-0.60781600
H	-1.31350100	0.25531800	0.58350700
O	3.02034900	-1.75901200	2.03040200
H	2.95260800	-1.61275400	2.98349100
H	3.91213700	-1.48836800	1.77633000

Cartesian coordinates of optimized geometries.

[Entinostat - $Zn(H_2O)_4]^{2+}$ in methanol

C	6.25997600	0.43741400	-1.21528400
C	5.19317100	1.32681300	-1.14258000
C	3.88491500	0.85474900	-1.03319700
C	3.63704500	-0.52766400	-1.02644900
C	4.71476400	-1.41301900	-1.07776000
C	6.01881200	-0.93478900	-1.16937500
H	7.27184300	0.81466800	-1.29544700
H	5.36466000	2.39670000	-1.15508700
H	4.52694400	-2.48124000	-1.07007700
H	6.84213100	-1.63702100	-1.21760500
N	2.29169900	-1.03660100	-0.94980500
H	2.27290900	-2.00762300	-1.25421900
N	2.80597900	1.78605200	-0.96023600
C	1.91696100	1.80681200	0.05369300
O	2.05840300	1.04383100	1.03914900
H	2.76978700	2.52684400	-1.64922900
Zn	1.29235100	-0.94415100	1.00410200
H	1.68601900	-0.52785400	-1.59274700
C	0.69597400	2.63682300	-0.06371500
C	0.05627300	3.04054000	1.11417400
C	0.02752700	2.75975800	-1.28882400
C	-1.26822900	3.44889500	1.07813700
H	0.57994800	2.96904800	2.05915600
C	-1.30725300	3.14841900	-1.31248100
H	0.50505500	2.45355400	-2.21197500
C	-1.98452100	3.42601600	-0.12228800
H	-1.77566200	3.70471300	2.00120000
H	-1.84320200	3.15799500	-2.25499300
C	-3.48948400	3.58874600	-0.10836700
H	-3.79922200	4.37513800	-0.79849300
H	-3.81960800	3.87803200	0.89298700
C	-4.04519100	1.16467100	0.04752400
H	-4.99232900	2.48311300	-1.14749800

N	-4.19366500	2.37273300	-0.54038500
O	-3.19483300	0.87153100	0.88152600
O	-4.97183800	0.28875600	-0.41808200
C	-5.04121000	-1.01575000	0.22198500
H	-6.06415000	-1.33830700	0.03510200
H	-4.89126500	-0.89476900	1.29386100
C	-4.05498900	-1.99967800	-0.35452200
C	-4.24095800	-2.58236600	-1.61097000
C	-2.90925400	-2.35315800	0.35593800
C	-3.28510200	-3.46578100	-2.10180900
H	-5.12268900	-2.34815900	-2.19719800
H	-2.72906400	-1.93641500	1.34063900
C	-2.15669700	-3.72735800	-1.33070000
H	-3.40274100	-3.93846400	-3.06848000
H	-1.37592900	-4.38891700	-1.68955200
N	-1.96715100	-3.17930600	-0.12147300
O	0.64981700	-0.55912800	3.01217700
H	0.59849000	0.36756000	3.27959900
H	-0.14352100	-0.99634500	3.34726000
O	0.43022300	-2.83206900	1.08579300
H	-0.44442100	-3.07279600	0.64186200
H	0.97710700	-3.62225000	1.15770200
O	-0.57116300	-0.21705600	0.15478600
H	-0.96248200	-0.67904300	-0.59541000
H	-1.30861300	0.26409300	0.57659000
O	3.01875600	-1.76930300	2.02261400
H	2.95092500	-1.63623200	2.97763400
H	3.91041800	-1.49470500	1.77215900

Cartesian coordinates of optimized geometries.

Mocetinostat in gas phase

N	5.96787000	2.46499800	0.76428800
C	6.85048100	1.51570300	0.26405000
C	8.23194800	1.77633600	0.21016200
C	9.12361300	0.88923000	-0.37485700
C	8.66434700	-0.31159200	-0.91618600
C	7.30540000	-0.59573000	-0.85789500
C	6.39495200	0.29643600	-0.28489200
N	5.00822700	-0.03804700	-0.38068800
C	4.06248500	-0.02032900	0.60506900
O	4.27097400	0.43677200	1.72588800
C	2.71576400	-0.57796700	0.23515600
C	2.51183000	-1.52136200	-0.77687200
C	1.23374800	-2.00516400	-1.04616800
C	0.13065500	-1.56322000	-0.31292700
C	-1.24749100	-2.13137500	-0.58810400

N	-2.28137700	-1.11113900	-0.59699200
C	-3.53270600	-1.26206500	-0.07633300
N	-3.80203900	-2.37299600	0.63183900
C	-5.02842100	-2.44129900	1.14848800
C	-5.98921300	-1.45566500	0.97160100
C	-5.61628400	-0.34716900	0.19506300
N	-4.38959000	-0.25198100	-0.32259200
C	-6.54846500	0.77467600	-0.07818300
C	-7.93788500	0.62229000	-0.03973200
C	-8.74879600	1.71926900	-0.30547500
C	-8.14431300	2.93907000	-0.60053200
N	-6.81869700	3.10969300	-0.64192500
C	-6.05031500	2.04957100	-0.39011800
C	0.33807600	-0.62429800	0.70428400
C	1.61202800	-0.14608300	0.98051600
H	6.42236800	3.23401200	1.23635800
H	5.21255500	2.06648400	1.31717600
H	8.59603900	2.70831100	0.63126800
H	10.17977600	1.13258400	-0.40186200
H	9.35120100	-1.01823300	-1.36539100
H	6.92910200	-1.53073400	-1.26076900
H	4.72203000	-0.40950900	-1.27324000
H	3.34411300	-1.91529500	-1.35028500
H	1.09638200	-2.74007600	-1.83275700
H	-1.52108500	-2.85043000	0.18661700
H	-1.23239000	-2.68052200	-1.53815100
H	-2.16497300	-0.30399800	-1.19157900
H	-5.24905500	-3.32971100	1.73460900
H	-6.96377400	-1.54082200	1.43042500
H	-8.38287800	-0.34293900	0.17234900
H	-9.82871000	1.63178500	-0.29324600
H	-8.74760700	3.81634600	-0.81604000
H	-4.97801400	2.20296900	-0.42872300
H	-0.50787200	-0.26924000	1.28186000
H	1.77562600	0.56557600	1.78006500

Cartesian coordinates of optimized geometries.

Mocetinostat in water

N	6.00699400	2.27469800	-0.99152900
C	6.70487100	1.38722900	-0.17330700
C	8.03193100	1.65716900	0.20826600
C	8.78334300	0.74049700	0.93214900
C	8.22376200	-0.47980300	1.31576100
C	6.90726200	-0.75722500	0.96342400
C	6.14438700	0.15397800	0.22675400
N	4.83733500	-0.25887200	-0.17910500
C	3.66714800	0.41517600	-0.00550500

O	3.62623600	1.57482300	0.41945200
C	2.41629100	-0.31877200	-0.38599600
C	2.29770300	-1.71442700	-0.34101500
C	1.09783300	-2.33025800	-0.68133200
C	-0.00879900	-1.57591500	-1.08507700
C	-1.28483400	-2.29340000	-1.50403000
N	-2.49313100	-1.49334200	-1.47097100
C	-3.22786300	-1.24728100	-0.35308600
N	-2.80774500	-1.77648200	0.81181100
C	-3.56946400	-1.50902400	1.87502500
C	-4.72155700	-0.73821700	1.81574800
C	-5.07702000	-0.23823800	0.55447500
N	-4.33164900	-0.49134500	-0.52528200
C	-6.28245600	0.60566200	0.35719300
C	-7.39441000	0.53186400	1.20287000
C	-8.48875100	1.35333100	0.95767500
C	-8.43887600	2.22829800	-0.12431400
N	-7.38294000	2.31987300	-0.94466000
C	-6.33919500	1.52234700	-0.70278400
C	0.10922000	-0.18445600	-1.12230300
C	1.30258500	0.43697100	-0.76833000
H	6.46211400	3.17281300	-1.09105200
H	5.03126100	2.38194800	-0.72809900
H	8.47197100	2.60384400	-0.08826800
H	9.80565600	0.98175400	1.20077700
H	8.79820600	-1.19974700	1.88576800
H	6.45055600	-1.69655800	1.25628700
H	4.77324700	-1.20546100	-0.52493000
H	3.12069100	-2.33489800	-0.00458500
H	1.02054300	-3.41094800	-0.62160400
H	-1.42002000	-3.17512900	-0.87460600
H	-1.16642600	-2.65079400	-2.53086300
H	-2.83731900	-1.08082400	-2.32365300
H	-3.23547600	-1.92673100	2.82065900
H	-5.29250400	-0.52397100	2.70738200
H	-7.41771600	-0.16885400	2.02881500
H	-9.36854800	1.31345600	1.58783100
H	-9.27859600	2.88053400	-0.34373900
H	-5.49206200	1.61069900	-1.37349300
H	-0.73916000	0.41880600	-1.42330700
H	1.38298200	1.51672500	-0.79035700

Cartesian coordinates of optimized geometries.

Mocetinostat in methanol

N	6.01067000	2.27411600	-0.98886200
C	6.70754700	1.38563600	-0.17111100
C	8.03511600	1.65358300	0.21001500
C	8.78550800	0.73603400	0.93374100
C	8.22433100	-0.48334300	1.31775800
C	6.90735300	-0.75883400	0.96582400

C	6.14548400	0.15316400	0.22916400
N	4.83798900	-0.25847500	-0.17645200
C	3.66817400	0.41742400	-0.00559100
O	3.62794800	1.57776000	0.41674300
C	2.41695100	-0.31606100	-0.38628400
C	2.29836300	-1.71183300	-0.34544800
C	1.09843900	-2.32680200	-0.68690300
C	-0.00857800	-1.57137100	-1.08746300
C	-1.28454900	-2.28792900	-1.50835500
N	-2.49295300	-1.48816300	-1.47404400
C	-3.22977300	-1.24742800	-0.35620300
N	-2.81251800	-1.78376800	0.80631700
C	-3.57562200	-1.52102500	1.86956300
C	-4.72664000	-0.74838900	1.81239300
C	-5.07941500	-0.24131200	0.55318300
N	-4.33227600	-0.48935300	-0.52651300
C	-6.28364200	0.60479800	0.35818200
C	-7.39662200	0.52874100	1.20226700
C	-8.48966200	1.35261700	0.95944300
C	-8.43745700	2.23213500	-0.11876200
N	-7.38065100	2.32582100	-0.93759400
C	-6.33821600	1.52600500	-0.69802300
C	0.10913600	-0.17983600	-1.11991400
C	1.30273300	0.44062100	-0.76504800
H	6.46692000	3.17162900	-1.08831300
H	5.03508500	2.38232400	-0.72507500
H	8.47640600	2.59962300	-0.08673000
H	9.80822900	0.97593100	1.20204300
H	8.79783300	-1.20394400	1.88788000
H	6.44933800	-1.69735300	1.25933500
H	4.77305000	-1.20533600	-0.52119800
H	3.12149300	-2.33335600	-0.01128900
H	1.02121700	-3.40768100	-0.63035500
H	-1.42012500	-3.17063100	-0.88042400
H	-1.16567100	-2.64352200	-2.53581800
H	-2.83546900	-1.07093000	-2.32508200
H	-3.24371000	-1.94427500	2.81347800
H	-5.29874000	-0.53796100	2.70421300
H	-7.42168500	-0.17561600	2.02508500
H	-9.37023300	1.31112100	1.58845200
H	-9.27607500	2.88642400	-0.33636800
H	-5.49035700	1.61588300	-1.36759200
H	-0.73975700	0.42434200	-1.41763000
H	1.38309000	1.52043300	-0.78308900

Cartesian coordinates of optimized geometries.

[Mocetinostat - Zn(H_2O)$_2$]$^{2+}$ in gas phase

N	5.15428800	0.90866100	1.46687600
C	5.90913500	-0.04928800	0.65334200
C	7.28171100	0.13286700	0.49277200
C	8.01833500	-0.75398200	-0.28683400
C	7.37866100	-1.82215400	-0.91312800
C	6.00754100	-1.99436400	-0.76422100
C	5.25183000	-1.10774000	0.01025300
N	3.85792400	-1.39267000	0.18899600
C	2.79013900	-0.64207500	-0.18571500
O	2.95129500	0.57040300	-0.55808300
C	1.46146600	-1.23380000	-0.17035900
C	1.17335000	-2.45219000	0.48231400
C	-0.10422400	-2.97756400	0.44389400
C	-1.13407100	-2.32394800	-0.25288200
C	-2.51157400	-2.95771900	-0.31557000
N	-3.56288400	-2.07022600	-0.75401300
C	-4.25063900	-1.27104400	0.14030400
N	-3.70991800	-1.10018800	1.35293100
C	-4.42163000	-0.32508400	2.18323600
C	-5.61798400	0.27073200	1.82566100
C	-6.08821800	0.03425200	0.52059100
N	-5.38944100	-0.74139900	-0.31782200
C	-7.34077200	0.62872900	0.00735500
C	-8.37994100	1.03974400	0.85069600
C	-9.52483400	1.59214100	0.29200800
C	-9.59469800	1.72338200	-1.09433900
N	-8.61237700	1.34208200	-1.91799700
C	-7.52289100	0.80435100	-1.37389700
C	-0.85014400	-1.11360600	-0.89523200
C	0.42237400	-0.57072700	-0.85267800
H	5.79546600	1.43225200	2.06224300
H	4.51931000	0.41538800	2.09831400
H	7.78381200	0.94751800	1.00572100
H	9.08796400	-0.61853500	-0.38862100
H	7.94489500	-2.52137300	-1.51566000
H	5.50812600	-2.82297300	-1.25376400
H	3.65571200	-2.37570500	0.32206700
H	1.92204400	-2.97778400	1.06647300
H	-0.31629700	-3.90106100	0.97124200
H	-2.73996600	-3.37629300	0.67025900
H	-2.46091500	-3.79855400	-1.01602500
H	-4.10329400	-2.33168100	-1.56502600
H	-4.00410000	-0.17818500	3.17523200
H	-6.14869500	0.90542600	2.52070600
H	-8.31240900	0.90722500	1.92432000
H	-10.35382500	1.90717900	0.91373900
H	-10.47645000	2.14787700	-1.56457700
H	-6.73898300	0.49766800	-2.05722400
H	-1.64536800	-0.60767900	-1.42829700
H	0.63349200	0.35425700	-1.37336500
Zn	4.06977700	1.97659000	0.09037300
O	5.25540000	2.93189400	-1.25915200
H	5.97041900	2.50215200	-1.74980000

H	5.00184700	3.72987000	-1.74462800
O	2.82362600	3.53118100	0.54449600
H	2.99365800	4.32581200	1.06975400
H	1.86701900	3.46236700	0.40938000

Cartesian coordinates of optimized geometries.

[Mocetinostat - $Zn(H_2O)_2]^{2+}$ in water

N	5.05649100	0.84046600	1.44143200
C	5.86005500	-0.09696000	0.69720100
C	7.24144700	0.08875700	0.63549400
C	8.04161600	-0.78508400	-0.08999900
C	7.46252500	-1.85805900	-0.76705000
C	6.08655000	-2.03805400	-0.72273500
C	5.27071100	-1.15530000	-0.00617900
N	3.87288800	-1.44545600	0.03602300
C	2.82715300	-0.64537700	-0.25956000
O	2.95633600	0.57301100	-0.53319100
C	1.48019300	-1.26626200	-0.28993900
C	1.18526700	-2.48212800	0.34532600
C	-0.08809900	-3.02761000	0.25299800
C	-1.09543000	-2.38455200	-0.47521900
C	-2.45395600	-3.05440500	-0.61440700
N	-3.56330600	-2.16392800	-0.89081600
C	-4.25597000	-1.48346500	0.06298100
N	-3.82728000	-1.56733900	1.33640400
C	-4.54522000	-0.88646700	2.23294800
C	-5.66205800	-0.13253700	1.90335600
C	-6.02906600	-0.11366500	0.54974400
N	-5.32607900	-0.78504500	-0.36729800
C	-7.20230000	0.65713600	0.06716000
C	-8.29902300	0.94113800	0.88786500
C	-9.36484000	1.66667300	0.36823600
C	-9.30197300	2.09135800	-0.95631500
N	-8.25978700	1.83563400	-1.75937300
C	-7.24389800	1.13287800	-1.25146600
C	-0.80420700	-1.16418200	-1.09035000
C	0.46516100	-0.60648200	-0.99561000
H	5.63823300	1.37538600	2.08294100
H	4.35564600	0.36759700	2.01179500
H	7.68701000	0.91764900	1.17372400
H	9.11327500	-0.63152300	-0.11845400
H	8.07681300	-2.54798800	-1.33197200
H	5.62556800	-2.86361200	-1.25222100
H	3.66077400	-2.43325400	0.08535100
H	1.92398100	-2.99806900	0.94777600
H	-0.30292100	-3.96099700	0.76176500

H	-2.66260500	-3.62670600	0.29162800
H	-2.40284600	-3.77087000	-1.43934200
H	-3.93510200	-2.11272800	-1.82608700
H	-4.20413900	-0.94333800	3.26278700
H	-6.19879200	0.42373200	2.65795200
H	-8.33340100	0.58532100	1.91058900
H	-10.23310100	1.89404700	0.97417600
H	-10.11965900	2.65721400	-1.39201900
H	-6.40775700	0.93661500	-1.91305300
H	-1.57614800	-0.64645400	-1.64690600
H	0.68443300	0.33572400	-1.48088900
Zn	4.01231400	2.13206600	0.14749600
O	4.76759800	3.48727500	-1.18958200
H	5.32208700	3.17209000	-1.91582900
H	4.12497600	4.10596700	-1.56256100
O	2.55985300	3.57079600	0.67156000
H	2.75371900	4.11436200	1.44734000
H	1.65193500	3.25477800	0.77485300

Cartesian coordinates of optimized geometries.

[Mocetinostat - $Zn(H_2O)_2]^{2+}$ in methanol

N	5.00870700	0.85962700	1.43093200
C	5.82174100	-0.10406900	0.72760800
C	7.20535900	0.07021700	0.69105300
C	8.01365200	-0.82600700	0.00263000
C	7.44009500	-1.90901500	-0.66271300
C	6.06210400	-2.07774500	-0.64354500
C	5.23821700	-1.17323900	0.03576400
N	3.83777700	-1.45424700	0.05678000
C	2.80028000	-0.65870900	-0.27626700
O	2.93745200	0.55342500	-0.57646800
C	1.45209600	-1.27520300	-0.31555800
C	1.14337700	-2.47893800	0.33623400
C	-0.13106700	-3.02060000	0.23620000
C	-1.12591800	-2.38557700	-0.51550300
C	-2.48642700	-3.04929600	-0.66266100
N	-3.59208100	-2.14920800	-0.92313800
C	-4.27203800	-1.47310200	0.04328700
N	-3.82842800	-1.56648300	1.31076800
C	-4.53409200	-0.88940000	2.21994500
C	-5.65255300	-0.13040000	1.90823000
C	-6.03527600	-0.10140200	0.55902100
N	-5.34451300	-0.76865900	-0.37021200
C	-7.21151000	0.67590400	0.09469300
C	-8.29579200	0.96250200	0.93089200
C	-9.36508900	1.69447300	0.42778200
C	-9.31799300	2.12284800	-0.89626900
N	-8.28805800	1.86457100	-1.71403700
C	-7.26890100	1.15549400	-1.22200100
C	-0.82101800	-1.17715600	-1.14780100

C	0.44930500	-0.62364100	-1.04649400
H	5.58384700	1.40736400	2.06786600
H	4.30179600	0.40150700	2.00630500
H	7.64669000	0.90713700	1.22035800
H	9.08688200	-0.68161700	-0.00609400
H	8.06044000	-2.61610500	-1.19900700
H	5.60637400	-2.91158800	-1.16453500
H	3.61952800	-2.43952900	0.12668400
H	1.87105600	-2.98801700	0.95776500
H	-0.35701000	-3.94389300	0.75823100
H	-2.69497200	-3.63605800	0.23416800
H	-2.43939600	-3.75164000	-1.49992800
H	-3.98016100	-2.09818500	-1.85172300
H	-4.18132400	-0.95404400	3.24539200
H	-6.17917600	0.42191400	2.67281100
H	-8.31821400	0.60374700	1.95292800
H	-10.22414500	1.92392000	1.04595700
H	-10.13874400	2.69382800	-1.31934200
H	-6.44299900	0.95672000	-1.89556500
H	-1.58360300	-0.66568400	-1.72279700
H	0.67939300	0.30847500	-1.54594500
Zn	3.98110400	2.11963300	0.09376300
O	5.19358000	3.39797700	-0.99090800
H	5.75425900	3.04393800	-1.69408500
H	4.75762200	4.18689000	-1.34068300
O	2.68071400	3.68789700	0.45645000
H	2.68532400	4.08170900	1.33954000
H	1.75429200	3.57900600	0.20142700

Cartesian coordinates of optimized geometries.

[Mocetinostat - $Zn(H_2O)_4]^{2+}$ in gas phase

N	4.66392300	0.60208900	1.33996600
C	5.58076500	-0.25860100	0.61158300
C	6.93630000	0.06500500	0.56723500
C	7.83504700	-0.74956100	-0.11421900
C	7.38081500	-1.89764400	-0.76172700
C	6.02886900	-2.21729500	-0.73263200
C	5.11718600	-1.39996200	-0.05758600
N	3.74606400	-1.80032200	-0.00413200
C	2.68796500	-1.06116200	-0.41316900
O	2.87264900	0.10316100	-0.87357600
C	1.33819800	-1.62471500	-0.29378100
C	1.03215300	-2.70383100	0.55861400
C	-0.26583000	-3.18075900	0.64094500
C	-1.29504900	-2.61068600	-0.12257600
C	-2.69891500	-3.18308100	-0.03561000
N	-3.73430600	-2.31403600	-0.54717500
C	-4.34976500	-1.37136000	0.25232000
N	-3.74969200	-1.05238700	1.40713300
C	-4.40600900	-0.15091300	2.15171400
C	-5.59964500	0.43094800	1.76492400
C	-6.13019400	0.03967600	0.52101800

N	-5.49088100	-0.86578700	-0.22903500
C	-7.38331200	0.60559700	-0.02165100
C	-8.37461000	1.16171600	0.79586300
C	-9.52337800	1.67601800	0.20956700
C	-9.64442100	1.62552300	-1.17864600
N	-8.70805300	1.10368000	-1.97842800
C	-7.61513800	0.60320600	-1.40666900
C	-0.98996900	-1.54038600	-0.97071700
C	0.30382900	-1.04942900	-1.05552200
H	5.19165400	1.13443200	2.02928300
H	4.00307900	0.03731000	1.87523700
H	7.29845300	0.94095000	1.09671200
H	8.88807000	-0.49663600	-0.12373800
H	8.07532500	-2.54162800	-1.28638600
H	5.66736800	-3.10496100	-1.23964500
H	3.57789100	-2.78516300	0.15821800
H	1.78784200	-3.15053700	1.19624500
H	-0.49047700	-3.99679100	1.31908700
H	-2.89740900	-3.45115300	1.00697100
H	-2.71985100	-4.11218000	-0.61508800
H	-4.32021000	-2.65644600	-1.29402400
H	-3.94451500	0.11256400	3.09945100
H	-6.08388900	1.16786800	2.38946500
H	-8.26864600	1.16958200	1.87454100
H	-10.31699200	2.09953300	0.81272900
H	-10.53071000	2.01552900	-1.66971100
H	-6.86952500	0.18017900	-2.07051400
H	-1.78348700	-1.10276700	-1.56393300
H	0.53659100	-0.24274600	-1.73982300
Zn	3.42048900	1.80981800	0.05137200
O	3.87776600	3.72390800	0.95457300
H	4.42946100	4.04441900	1.67885400
H	3.44034800	4.48838000	0.55596100
O	2.21242100	3.14293100	-1.16222100
H	1.24680100	3.11814100	-1.15685900
H	2.48957700	3.23271800	-2.08429500
O	1.62884900	1.57331500	1.33324800
H	1.28195700	2.10906500	2.05774200
H	0.96732200	0.89599000	1.12884800
O	4.81398800	2.15105800	-1.58098400
H	5.00881400	1.36589200	-2.11077300
H	5.59635100	2.71645800	-1.60799500

Cartesian coordinates of optimized geometries.

[Mocetinostat - $Zn(H_2O)_4]^{2+}$ in water

N	4.79001500	0.49301000	1.25505600
C	5.57727600	-0.34699800	0.39492800
C	6.92824500	-0.05517900	0.19595000
C	7.71052300	-0.84909800	-0.63402600
C	7.14795100	-1.95060400	-1.27920500
C	5.80096600	-2.23761500	-1.09957000

C	5.00412800	-1.43630200	-0.27580800
N	3.63727300	-1.80705400	-0.09920100
C	2.56025600	-1.02581900	-0.33622700
O	2.68159300	0.17319700	-0.67797100
C	1.21695700	-1.63895900	-0.19533600
C	0.97399300	-2.76971800	0.59921600
C	-0.30952300	-3.29016900	0.69733100
C	-1.37733900	-2.70899800	0.00242400
C	-2.75742700	-3.34284700	0.10603200
N	-3.85917200	-2.52492700	-0.35617400
C	-4.47798200	-1.56772800	0.38738300
N	-4.02448300	-1.34130100	1.63474100
C	-4.67173000	-0.39606200	2.32072200
C	-5.74066900	0.32411400	1.80748000
C	-6.13902900	0.00940000	0.49994000
N	-5.50839900	-0.93294600	-0.20710000
C	-7.26294800	0.71240900	-0.16812600
C	-8.31077100	1.29959800	0.54904500
C	-9.33015400	1.94237100	-0.14392200
C	-9.27106500	1.98304200	-1.53447900
N	-8.27507500	1.43205900	-2.24217700
C	-7.30379300	0.81252400	-1.56648700
C	-1.13189700	-1.58122500	-0.78406300
C	0.14709800	-1.04426600	-0.87538000
H	5.39475800	0.99297500	1.90293600
H	4.14818900	-0.05426500	1.82795700
H	7.36714800	0.79154900	0.71186300
H	8.75890700	-0.61211700	-0.76805800
H	7.75121100	-2.57781000	-1.92366800
H	5.34741100	-3.08325900	-1.60345800
H	3.46643700	-2.79915200	-0.00002400
H	1.76692700	-3.22789000	1.17901500
H	-0.48485300	-4.15237500	1.33184400
H	-2.93541200	-3.63535400	1.14288800
H	-2.76474800	-4.26115300	-0.48766500
H	-4.22659000	-2.66869700	-1.28366400
H	-4.31033100	-0.20203000	3.32667000
H	-6.21700500	1.10032600	2.38846000
H	-8.34456000	1.24044100	1.63028900
H	-10.16042500	2.39817800	0.38104700
H	-10.05339700	2.47483600	-2.10435300
H	-6.50423600	0.37477600	-2.15329200
H	-1.94703700	-1.11615500	-1.32484700
H	0.32862600	-0.16767200	-1.48399600
Zn	3.50799200	1.86854900	0.20066900
O	4.18523800	3.67750000	1.11995200

H	5.10948500	3.86899900	1.32471200
H	3.81216200	4.47679800	0.72449900
O	2.12109700	3.20281700	-0.75483000
H	1.16846600	3.09897200	-0.63556400
H	2.27763100	3.28826400	-1.70479500
O	2.13323900	1.76329400	1.88701100
H	2.11028700	2.52465200	2.48110800
H	1.22782000	1.43451900	1.81826600
O	4.55441800	2.19226000	-1.66194000
H	4.65306400	1.41513900	-2.22760600
H	5.39611000	2.66538800	-1.69134200

Cartesian coordinates of optimized geometries.
[Mocetinostat - Zn(H_2O)$_4$]$^{2+}$ in methanol

N	4.78960300	0.49644900	1.25804700
C	5.58060800	-0.34703700	0.40393700
C	6.93251400	-0.05673600	0.20994700
C	7.71780000	-0.85375800	-0.61430600
C	7.15719500	-1.95664100	-1.25867700
C	5.80932800	-2.24242200	-1.08369400
C	5.00977400	-1.43828100	-0.26546400
N	3.64201600	-1.80765400	-0.09213800
C	2.56673200	-1.02703400	-0.33852900
O	2.69159700	0.16978400	-0.68818600
C	1.22241800	-1.63684500	-0.19753600
C	0.97503400	-2.75820800	0.60894600
C	-0.30958600	-3.27538300	0.70889400
C	-1.37415400	-2.70046700	0.00397600
C	-2.75578500	-3.33000200	0.11073000
N	-3.85461200	-2.51086400	-0.35608500
C	-4.47304700	-1.55151200	0.38535100
N	-4.00836200	-1.31120500	1.62585800
C	-4.65540700	-0.36445100	2.31005000
C	-5.73428800	0.34384200	1.80129400
C	-6.14333400	0.01544400	0.50028600
N	-5.51285300	-0.92844900	-0.20475800
C	-7.27861500	0.70472800	-0.16272600
C	-8.32202800	1.29364800	0.55946400
C	-9.35248400	1.92313000	-0.12925400
C	-9.30849000	1.94930600	-1.52075700
N	-8.31720600	1.39623600	-2.23325400
C	-7.33540700	0.78927200	-1.56158600
C	-1.12422100	-1.58260400	-0.79523900
C	0.15581500	-1.04871100	-0.88858200
H	5.39226000	0.99773500	1.90689800
H	4.14613400	-0.04951300	1.83057700
H	7.37053500	0.79045600	0.72591500
H	8.76702800	-0.61832700	-0.74417700
H	7.76281500	-2.58619000	-1.89857200
H	5.35747900	-3.08943200	-1.58686100
H	3.47010700	-2.79902200	0.01304400

H	1.76532300	-3.21074000	1.19669900
H	-0.48848800	-4.12962100	1.35307500
H	-2.93401400	-3.61704900	1.14917600
H	-2.76548300	-4.25106100	-0.47866700
H	-4.23771600	-2.67359200	-1.27399200
H	-4.28557800	-0.15942900	3.31076200
H	-6.21061100	1.12128600	2.38063800
H	-8.34404500	1.24580800	1.64158600
H	-10.17980100	2.37964000	0.39978000
H	-10.09962300	2.43061100	-2.08742600
H	-6.54017300	0.34894300	-2.15229800
H	-1.93716700	-1.12310100	-1.34402300
H	0.34081000	-0.18023300	-1.50773200
Zn	3.50893300	1.86476500	0.19075700
O	4.18373300	3.67255700	1.11234800
H	5.10124300	3.86757900	1.34200900
H	3.81333600	4.47396300	0.71873200
O	2.13892000	3.20685000	-0.78015100
H	1.18375400	3.11222600	-0.67450100
H	2.30968900	3.29540100	-1.72739400
O	2.11400300	1.75914500	1.85920800
H	2.08878200	2.51485600	2.46039600
H	1.20736100	1.43711900	1.77580100
O	4.57769300	2.19238500	-1.65870500
H	4.67917500	1.41916400	-2.22927500
H	5.41898100	2.66655500	-1.68182900

Cartesian coordinates of optimized geometries.

Tacedinaline in gas phase

C	-6.93448100	0.52910000	-0.01981500
C	-5.44462600	0.77037200	-0.19211500
O	-5.01142300	1.83770500	-0.58441000
N	-4.64710800	-0.31003800	0.13023200
C	-3.24438200	-0.42578800	0.08624700
C	-2.68524300	-1.64426000	0.50424800
C	-1.31485900	-1.83674200	0.48475400
C	-0.45421900	-0.81580400	0.06246700
C	-1.01999500	0.39055300	-0.36326700
C	-2.39669200	0.59689400	-0.35747900
C	1.01824800	-1.11208100	0.06915100
O	1.43589600	-2.26113400	0.09771700
N	1.83995600	-0.00948700	0.06676500
C	3.24549000	0.03906600	-0.02754900
C	4.03443200	-1.02245100	-0.47962100
C	5.40903500	-0.85254500	-0.63102000
C	6.00637900	0.36975000	-0.33583600
C	5.22717000	1.41993400	0.14505900
C	3.85254700	1.26655400	0.31805200
N	3.02107800	2.32711800	0.76937000
H	-7.42469000	0.69309900	-0.98158100
H	-7.32659000	1.27117300	0.67852700
H	-7.18747500	-0.46931400	0.34242200

H	-5.13040300	-1.13630900	0.44784100
H	-3.33458700	-2.44596200	0.84340400
H	-0.88450800	-2.78099900	0.79383100
H	-0.39761700	1.19304300	-0.74447600
H	-2.81678700	1.53045400	-0.69723500
H	1.40560700	0.90215200	0.10632600
H	3.56251000	-1.96539200	-0.71067300
H	6.00936100	-1.68159600	-0.98703900
H	7.07450200	0.50455400	-0.46047200
H	5.68900200	2.36756600	0.40459200
H	3.52585300	3.19841500	0.86969200
H	2.54972200	2.11808100	1.64415700

Cartesian coordinates of optimized geometries.

Tacedinaline in water

C	-6.97297600	0.36844800	-0.23390500
C	-5.49541300	0.69798800	-0.20239100
O	-5.09161300	1.85175300	-0.29636500
N	-4.66386900	-0.38570300	-0.05375800
C	-3.26111500	-0.43260400	0.02791400
C	-2.67003200	-1.69718800	0.18863600
C	-1.29467400	-1.82552700	0.28192100
C	-0.45844000	-0.70318800	0.20427400
C	-1.05523100	0.55293200	0.04771000
C	-2.43660700	0.69953900	-0.03687400
C	1.01988500	-0.92708000	0.32217600
O	1.47084400	-1.95626000	0.82323000
N	1.82256800	0.06983100	-0.16385900
C	3.24585400	0.06243900	-0.19384000
C	3.96442900	-1.05376500	-0.62421500
C	5.35491100	-1.01926100	-0.69411200
C	6.03033000	0.15135300	-0.34481000
C	5.32254000	1.26904800	0.08378100
C	3.92296600	1.24327800	0.17820100
N	3.20259600	2.37743000	0.56285000
H	-7.41024600	0.81608800	-1.12823200
H	-7.44969400	0.82924300	0.63457200
H	-7.18681500	-0.70044400	-0.22791000
H	-5.12184500	-1.28365000	0.01193000
H	-3.29748700	-2.58040200	0.24152300
H	-0.85039000	-2.80396800	0.41229500
H	-0.45819200	1.45675200	0.01553700
H	-2.87536000	1.67811200	-0.14645500
H	1.37370000	0.86006900	-0.60546100
H	3.42339200	-1.94698400	-0.90618500
H	5.90186900	-1.89310600	-1.02720800
H	7.11227200	0.19450900	-0.39987600

H	5.85092900	2.17444700	0.36356400
H	3.76164100	3.09504800	1.00451000
H	2.36474800	2.19096600	1.09904800

Cartesian coordinates of optimized geometries.
Tacedinaline in methanol

C	-6.97023500	0.37980600	-0.23385600
C	-5.49183200	0.70533300	-0.19466800
O	-5.08506200	1.85889900	-0.27277500
N	-4.66324900	-0.38264800	-0.05882200
C	-3.26067600	-0.43404500	0.02388300
C	-2.67255500	-1.70189300	0.16835000
C	-1.29756400	-1.83464800	0.26070700
C	-0.45864400	-0.71335700	0.19877200
C	-1.05273900	0.54604100	0.05895100
C	-2.43364100	0.69700300	-0.02491900
C	1.01912200	-0.94351200	0.31422200
O	1.46643800	-1.98416700	0.79369700
N	1.82465500	0.06217500	-0.14948200
C	3.24698800	0.05914900	-0.17975300
C	3.97346300	-1.06003700	-0.58902400
C	5.36355600	-1.01515900	-0.66290100
C	6.03103600	0.16702800	-0.33947500
C	5.31509200	1.28725000	0.06921800
C	3.91663200	1.25186600	0.16788300
N	3.18618400	2.38775700	0.53237800
H	-7.40280100	0.83420800	-1.12709800
H	-7.44916100	0.83665200	0.63542200
H	-7.18707900	-0.68854000	-0.23555300
H	-5.12351900	-1.28017400	-0.00539200
H	-3.30198300	-2.58439600	0.20924900
H	-0.85521200	-2.81554200	0.37852300
H	-0.45421600	1.44926900	0.03932400
H	-2.87027000	1.67787200	-0.12184200
H	1.37713000	0.86329000	-0.57237400
H	3.43944300	-1.96305000	-0.85131700
H	5.91640400	-1.89147700	-0.97943600
H	7.11243900	0.21745300	-0.39833000
H	5.83669700	2.20219500	0.33011600
H	3.74463200	3.12420700	0.94288000
H	2.36535300	2.20425500	1.09558300

Cartesian coordinates of optimized geometries.
[Tacedinaline - $Zn(H_2O)_2$]$^{2+}$ in gas phase

C	7.87756600	-0.28918700	0.04946000
C	6.46552300	0.20644100	0.23376000
O	6.16821900	1.27526100	0.70864000
N	5.47962300	-0.71719200	-0.19839200
C	4.11008400	-0.59784900	-0.14366700
C	3.32945300	-1.65478400	-0.67512000

C	1.95634200	-1.60461000	-0.64771900
C	1.28116000	-0.49008100	-0.09464000
C	2.06869900	0.55832000	0.43063700
C	3.44565000	0.51797300	0.41628900
C	-0.15770200	-0.38043700	-0.01880800
N	-0.94486200	-1.42180600	-0.41394700
C	-2.32706600	-1.64277800	-0.09609500
C	-2.65725800	-2.74619000	0.69701800
C	-3.98477400	-3.05869900	0.96786900
C	-5.00668100	-2.26682900	0.44764900
C	-4.69477200	-1.16821000	-0.34834600
C	-3.36407800	-0.86570600	-0.63054500
N	-3.05501200	0.30641500	-1.45451100
H	8.07839800	-1.11948400	0.73367500
H	8.56423300	0.52394700	0.27309500
H	8.05107700	-0.64125100	-0.97106900
H	5.83306500	-1.57585500	-0.59956300
H	3.82234300	-2.51505000	-1.11395700
H	1.42383500	-2.44040000	-1.08919400
H	1.57677100	1.40926000	0.88237900
H	4.02655500	1.32544800	0.83280600
H	-1.86111100	-3.35940000	1.10407700
H	-4.21903000	-3.91884900	1.58258400
H	-6.04351000	-2.50935800	0.64514500
H	-5.49075300	-0.56737200	-0.77745200
H	-3.88582900	0.59368000	-1.97087300
H	-2.34957500	0.06824700	-2.15512100
O	-0.69966100	0.69598700	0.41920400
H	-0.44385200	-2.27131800	-0.63828200
Zn	-2.26975400	1.64231300	-0.10841200
O	-3.59692600	2.10017200	1.36808100
H	-4.04566400	1.43682900	1.91135700
H	-3.61415800	2.93845700	1.85127700
O	-1.71454800	3.55332600	-0.58623400
H	-0.79825400	3.85410300	-0.49832500
H	-2.19972600	4.22482500	-1.08622000

Cartesian coordinates of optimized geometries.

[Tacedinaline - $Zn(H_2O)_2]^{2+}$ in water

C	7.85852700	-0.31023800	0.15709700
C	6.43927900	0.21374200	0.15245000
O	6.17548400	1.38649600	0.37998900
N	5.47754100	-0.74365800	-0.09823100
C	4.08363700	-0.62322500	-0.09846700
C	3.33694400	-1.79757400	-0.30556100
C	1.95474400	-1.76733100	-0.30117500

C	1.26507700	-0.56022600	-0.09525500
C	2.01747100	0.60607000	0.10355000
C	3.40382700	0.58941100	0.10128400
C	-0.20806000	-0.45695100	-0.02146700
N	-0.95289500	-1.51344300	-0.41407400
C	-2.33494900	-1.76361800	-0.13238700
C	-2.65292700	-2.93388200	0.56306800
C	-3.97650700	-3.26605100	0.82449500
C	-4.99929200	-2.42268800	0.39241600
C	-4.69321300	-1.26382500	-0.31148400
C	-3.36679800	-0.93658600	-0.59128100
N	-3.07108300	0.26708400	-1.32982900
H	8.15232700	-0.50983800	1.19190800
H	8.51952000	0.46010400	-0.23865500
H	7.98110600	-1.22844200	-0.41827300
H	5.82515200	-1.67742400	-0.26723300
H	3.84909600	-2.74121500	-0.45545200
H	1.43594900	-2.70871000	-0.43524700
H	1.50091900	1.54189800	0.26809400
H	3.96162500	1.49812800	0.25851300
H	-1.84868900	-3.57595400	0.90208500
H	-4.20577300	-4.17458500	1.36718900
H	-6.03482100	-2.66962400	0.59149200
H	-5.48576300	-0.61484300	-0.66630800
H	-3.90215900	0.60394500	-1.81153800
H	-2.36676400	0.09806200	-2.04814500
O	-0.72850600	0.60043800	0.41764500
H	-0.44329100	-2.32298300	-0.74088100
Zn	-2.30021200	1.74039600	-0.07049300
O	-3.59989700	2.70497500	1.24054900
H	-4.11675900	2.17039800	1.85795700
H	-3.20335300	3.42472200	1.74992400
O	-1.48802600	3.61575000	-0.42156100
H	-0.53104200	3.69070700	-0.53549800
H	-1.89487400	4.15044700	-1.11688300

Cartesian coordinates of optimized geometries.

[Tacedinaline - $Zn(H_2O)_2]^{2+}$ in methanol

C	7.79680900	-0.40346100	-0.08582200
C	6.39040700	0.04673700	0.24385600
O	6.16772100	0.99355800	0.98588300
N	5.39406300	-0.70991100	-0.33777100
C	4.00462800	-0.60421500	-0.20320400
C	3.21899600	-1.54594200	-0.89252800
C	1.83894800	-1.51505400	-0.80573600
C	1.19586800	-0.53408200	-0.03285000
C	1.98297000	0.41297600	0.63611300

C	3.36795200	0.38295300	0.56708200
C	-0.27190100	-0.44410000	0.10638900
N	-1.00354600	-1.54974700	-0.15773600
C	-2.40530500	-1.73605700	0.04670000
C	-2.80914100	-2.81589900	0.83841100
C	-4.15712900	-3.08530100	1.03754800
C	-5.12109800	-2.26969100	0.44612300
C	-4.73021600	-1.20174600	-0.35290800
C	-3.37813400	-0.93995600	-0.57116800
N	-2.99879300	0.17140100	-1.41630700
H	8.19823900	-0.94450800	0.77571500
H	8.41829800	0.47756300	-0.24649100
H	7.85299800	-1.05272800	-0.95988300
H	5.70909800	-1.46354100	-0.93257100
H	3.69827300	-2.30119900	-1.50494200
H	1.27606800	-2.24279500	-1.37847400
H	1.49777600	1.17529000	1.23194400
H	3.95820800	1.11043200	1.10008300
H	-2.05193000	-3.43642700	1.30283800
H	-4.45175900	-3.92359600	1.65634700
H	-6.17509800	-2.46784500	0.59638700
H	-5.47637900	-0.57509000	-0.82865000
H	-3.78845500	0.46276700	-1.98899600
H	-2.25860900	-0.09749000	-2.06515700
O	-0.80401200	0.63238700	0.47938700
H	-0.48107200	-2.40666400	-0.28372900
Zn	-2.27279700	1.78446900	-0.31751700
O	-3.22161900	2.79928500	1.23520300
H	-3.77924100	2.31809900	1.86039900
H	-2.64543300	3.38079800	1.74908700
O	-1.04662400	3.46331300	-0.39492800
H	-0.10080500	3.28832800	-0.49489500
H	-1.26397400	4.19100600	-0.99318500

Cartesian coordinates of optimized geometries.

[Tacedinaline - $Zn(H_2O)_4]^{2+}$ in gas phase

C	7.99606900	0.19174100	0.06705600
C	6.56262000	-0.19367800	-0.20211900
O	6.22564900	-1.18726100	-0.80237800
N	5.61867900	0.73214500	0.29502500
C	4.24385700	0.70878500	0.16486700
C	3.49568000	1.70590900	0.83258900
C	2.12125900	1.74635600	0.73778800
C	1.42208100	0.78893100	-0.02848700
C	2.17452800	-0.20285100	-0.68868600
C	3.55212200	-0.25374900	-0.60383200
C	-0.02928200	0.78020800	-0.18446500
N	-0.74045400	1.87690500	0.18753300
C	-2.14711000	2.07143700	0.02520800
C	-2.58729200	3.19488200	-0.68281500

C	-3.94351300	3.46808500	-0.81148800
C	-4.88004600	2.61383300	-0.23102700
C	-4.45244700	1.49794200	0.48100500
C	-3.09201800	1.22695000	0.62393600
N	-2.67265300	0.05655400	1.37440200
H	8.31477200	0.93999000	-0.66572700
H	8.62238800	-0.69021700	-0.04951900
H	8.13664700	0.61220300	1.06562200
H	6.00005500	1.50970400	0.81661600
H	4.00903600	2.44453000	1.43826500
H	1.60044800	2.51339200	1.30086900
H	1.65957400	-0.92229400	-1.31288500
H	4.11124000	-1.01253100	-1.12818600
H	-1.85434500	3.85316600	-1.13594000
H	-4.26677400	4.34364600	-1.36037700
H	-5.93914800	2.82328900	-0.31667700
H	-5.18267700	0.85110000	0.95764300
H	-3.41778000	-0.21111700	2.01475500
H	-1.87028100	0.28507900	1.96263800
O	-1.36941200	-3.22224200	-1.12594900
H	-1.56693600	-3.14489300	-2.06938200
H	-0.50608300	-3.64874500	-1.05052000
O	-3.33134800	-3.06621500	0.80452500
H	-4.04674400	-3.12814000	1.44954700
H	-3.24443400	-3.92900300	0.37715200
O	-3.21142700	-1.17449800	-1.66161400
H	-2.99600600	-0.36954100	-2.15214300
H	-4.15840100	-1.32948500	-1.77022600
O	-0.44009500	-2.19719400	1.54342300
H	0.47907700	-1.90556400	1.47122400
H	-0.47793800	-2.87041100	2.23470100
O	-0.63129800	-0.23042400	-0.65777300
H	-0.20787600	2.71908100	0.36135600
Zn	-1.97308000	-1.52794900	0.09211400

Cartesian coordinates of optimized geometries.

[Tacedinaline - $Zn(H_2O)_4]^{2+}$ in water

C	8.04660400	0.11787400	0.19297700
C	6.62331500	-0.18266600	-0.22416700
O	6.35664100	-1.08348200	-1.00920400
N	5.66906300	0.62926900	0.34888800
C	4.27740200	0.61978400	0.18613600
C	3.54162200	1.56957000	0.91697400
C	2.16383900	1.63078000	0.81062900
C	1.47314800	0.74071900	-0.02769500
C	2.21029300	-0.21308200	-0.74141100

C	3.59357600	-0.27727500	-0.65063400
C	0.00194700	0.74930600	-0.18250200
N	-0.66536200	1.87189900	0.17174600
C	-2.06490600	2.10847600	0.02640400
C	-2.47147000	3.24886000	-0.67368400
C	-3.81968700	3.55419200	-0.80936700
C	-4.77827400	2.71264600	-0.24454700
C	-4.38238100	1.58420700	0.46337900
C	-3.02845200	1.28013100	0.61870500
N	-2.64358800	0.10328800	1.34688300
H	8.63861700	0.30853900	-0.70433100
H	8.45937800	-0.76826900	0.68027500
H	8.13796700	0.96876100	0.86799300
H	6.01862900	1.33436500	0.98240300
H	4.05665600	2.25609500	1.57924300
H	1.63795600	2.35718500	1.41850500
H	1.68697600	-0.90679200	-1.38673800
H	4.14571800	-1.01022900	-1.21611100
H	-1.71688800	3.88813000	-1.11698600
H	-4.11910700	4.43825400	-1.35840900
H	-5.83296900	2.93734600	-0.34652800
H	-5.12514100	0.93638300	0.91569000
H	-3.38574900	-0.17523100	1.98460800
H	-1.82131400	0.27446000	1.92473700
O	-1.41911600	-3.20438000	-1.10710900
H	-1.59487800	-3.09546000	-2.05116100
H	-0.52582500	-3.56210700	-1.02667000
O	-3.51660000	-2.94722900	0.78159300
H	-4.43054200	-2.72462900	1.00067800
H	-3.54028300	-3.77169600	0.27751900
O	-3.19502800	-1.12738400	-1.71232400
H	-2.96642600	-0.32185600	-2.19422700
H	-4.15834900	-1.19669500	-1.72662200
O	-0.80258500	-2.26518400	1.68422300
H	0.15860200	-2.32305400	1.61005500
H	-1.09423400	-3.04756600	2.16992000
O	-0.59863500	-0.25248800	-0.63859900
H	-0.10680300	2.69446200	0.35629900
Zn	-2.06532200	-1.53805100	0.07771400

Cartesian coordinates of optimized geometries.

[Tacedinaline - $Zn(H_2O)_4]^{2+}$ in methanol

C	8.04846600	0.13469400	0.17457300
C	6.62193700	-0.19308800	-0.20976300
O	6.35127800	-1.11522400	-0.96734900
N	5.66894600	0.63316600	0.34701600

C	4.27796100	0.62378400	0.18332800
C	3.54303400	1.58684600	0.89781200
C	2.16554200	1.64809700	0.79026500
C	1.47333500	0.74548100	-0.03362600
C	2.20994200	-0.22019100	-0.73219900
C	3.59288800	-0.28540900	-0.63946000
C	0.00247900	0.75189900	-0.18779300
N	-0.66747500	1.87365400	0.16461900
C	-2.06812800	2.10658400	0.02354700
C	-2.48003700	3.24556600	-0.67565900
C	-3.82940300	3.54861800	-0.80483700
C	-4.78382600	2.70652100	-0.23398200
C	-4.38261900	1.57928300	0.47292900
C	-3.02752100	1.27731800	0.62128300
N	-2.63654900	0.10143700	1.34834900
H	8.56744600	0.51039300	-0.71095100
H	8.54582400	-0.78530800	0.48509000
H	8.13170000	0.87537100	0.97013300
H	6.01995700	1.35325700	0.96252900
H	4.05866700	2.28377100	1.54864900
H	1.64147500	2.38549800	1.38639000
H	1.68619600	-0.92239200	-1.36786900
H	4.14426700	-1.02826000	-1.19264600
H	-1.72884700	3.88600500	-1.12302300
H	-4.13296000	4.43182900	-1.35291200
H	-5.83927700	2.93014000	-0.33007600
H	-5.12229800	0.93171000	0.93067200
H	-3.37335100	-0.17684900	1.99231300
H	-1.81068100	0.27511100	1.92038100
O	-1.42381500	-3.20429100	-1.11117500
H	-1.60020200	-3.09579300	-2.05516600
H	-0.53402600	-3.57070200	-1.03109300
O	-3.51880200	-2.94025100	0.78420700
H	-4.42859700	-2.71550000	1.01778100
H	-3.55146600	-3.76658800	0.28375700
O	-3.19989900	-1.12565900	-1.70718300
H	-2.97360800	-0.32077900	-2.19129200
H	-4.16319500	-1.19586600	-1.71779700
O	-0.79369100	-2.26977700	1.67719800
H	0.16813800	-2.31842400	1.60461200
H	-1.07897600	-3.05509400	2.16193100
O	-0.59714100	-0.25235800	-0.64078700
H	-0.11106100	2.69825200	0.34622400
Zn	-2.05991000	-1.53810300	0.07615600

Cartesian coordinates of optimized geometries.

Zabadinostat in gas phase.

C	7.07738300	-1.77027500	-0.31065000
C	6.89196000	-0.28678300	-0.37098900
N	7.85937500	0.53809000	0.02978300
N	7.37670600	1.78521800	-0.16194600
C	6.12238000	1.76631700	-0.67823100

C	5.76005400	0.44085500	-0.83105300
C	4.47290700	-0.10044800	-1.37832700
N	3.68472100	-0.85708500	-0.39618900
C	2.58143600	-1.57636700	-1.03478300
C	1.87989500	-2.49412600	-0.03413900
C	1.32534300	-1.72240600	1.19007700
C	2.45500800	-0.83628100	1.75260300
C	3.18876400	-0.00963700	0.68940300
C	0.00112600	-1.01793600	0.89394900
C	-0.20331100	0.35978600	1.02507100
C	-1.44419500	0.93707200	0.77053500
C	-2.53771900	0.15709600	0.38880100
C	-2.34639600	-1.22551600	0.26270800
C	-1.10346800	-1.79400600	0.51038300
C	-3.83623600	0.87451500	0.13305000
O	-3.87785800	2.09206800	0.03131600
N	-4.94771200	0.07164100	0.02566900
C	-6.29478800	0.45158900	-0.14997800
C	-6.77622000	1.75012800	0.04035800
C	-8.14103700	2.00968200	-0.07072100
C	-9.03397100	0.98416500	-0.36720400
C	-8.55392100	-0.30540300	-0.58725200
C	-7.19136800	-0.58334500	-0.49711800
N	-6.67035600	-1.89364400	-0.68232000
C	8.20370600	2.93416300	0.15132000
H	8.04837900	-2.00519400	0.12733100
H	7.03539800	-2.21512700	-1.31071200
H	6.29034400	-2.23953800	0.28451100
H	5.58502000	2.67646500	-0.89779600
H	4.68793600	-0.78498200	-2.20622700
H	3.88237200	0.73112700	-1.80820500
H	2.99015600	-2.17644200	-1.85325300
H	1.84999800	-0.87548500	-1.48264200
H	2.60661500	-3.23152300	0.32206600
H	1.08242500	-3.04422100	-0.54034100
H	1.08996300	-2.47044600	1.95715600
H	3.18936800	-1.49606000	2.22533000
H	2.07890300	-0.17540700	2.53916600
H	4.04119100	0.49328900	1.15109200
H	2.52983000	0.78190900	0.28507500
H	0.60914000	1.00474000	1.33202800
H	-1.58546500	2.00664800	0.86435300
H	-3.15358900	-1.88308300	-0.04210900
H	-0.99268200	-2.86875700	0.41031400
H	-4.83385800	-0.91562500	0.20880400
H	-6.07768900	2.53734100	0.27896400

H	-8.50196200	3.01985100	0.08361900
H	-10.09627100	1.18435100	-0.44505700
H	-9.23967300	-1.10579200	-0.84773200
H	-7.40061500	-2.58335700	-0.80641500
H	-6.02786200	-1.95325300	-1.46658900
H	9.08231100	2.96009100	-0.49724900
H	8.53171300	2.88418400	1.19099700
H	7.61833800	3.84118900	0.00113500

Cartesian coordinates of optimized geometries.
Zabadinostat in water

C	7.21751700	-1.72409200	-0.50112000
C	6.93838200	-0.25307100	-0.50412700
N	7.88535100	0.62296600	-0.15365700
N	7.31280200	1.84412900	-0.26645000
C	6.02785300	1.75713600	-0.68358700
C	5.73371700	0.41437900	-0.85093400
C	4.44343100	-0.18948500	-1.32190900
N	3.69207200	-0.89341300	-0.26930200
C	2.60103800	-1.68724900	-0.84519300
C	1.90896500	-2.52735000	0.22728300
C	1.33619400	-1.66418900	1.37971300
C	2.44928100	-0.71687200	1.87011800
C	3.16978600	0.03148500	0.74243100
C	0.00459500	-1.00517200	1.01862000
C	-0.22462900	0.37533400	1.06217800
C	-1.47040700	0.91365700	0.74879900
C	-2.54319000	0.08967000	0.39868000
C	-2.32871500	-1.29530800	0.36265000
C	-1.07982400	-1.82413700	0.66464200
C	-3.85198700	0.74414900	0.06251400
O	-3.90455900	1.91495500	-0.31055000
N	-4.96736700	-0.03706500	0.20376900
C	-6.31198000	0.37284000	-0.02810500
C	-6.81403900	1.56312000	0.49938400
C	-8.14495900	1.92214700	0.30193800
C	-8.98443700	1.07022300	-0.41815500
C	-8.49375400	-0.11868100	-0.94649800
C	-7.14986500	-0.48408500	-0.77278700
N	-6.66237400	-1.69795100	-1.26221000
C	8.08051800	3.04204100	0.02995200
H	8.28638800	-1.90574800	-0.37663900
H	6.89536000	-2.18927900	-1.43729200
H	6.68619600	-2.23066200	0.30967700
H	5.42239000	2.63777500	-0.83401900
H	4.65310600	-0.92358300	-2.10656300

H	3.82335800	0.59809100	-1.78749500
H	3.02107500	-2.34677400	-1.60984500
H	1.86220900	-1.03656200	-1.35164800
H	2.64197800	-3.22198000	0.65041100
H	1.12539100	-3.13271500	-0.23439800
H	1.10570700	-2.34902100	2.20443400
H	3.19002800	-1.32453700	2.39966700
H	2.06271800	0.00207900	2.59799200
H	4.00552500	0.59525400	1.16256300
H	2.49477300	0.76757000	0.26876400
H	0.56914700	1.05361700	1.34498100
H	-1.62058700	1.98573300	0.77982100
H	-3.11903700	-1.97918600	0.07506200
H	-0.94947100	-2.90009900	0.62725000
H	-4.84147200	-0.97025300	0.57085600
H	-6.15218000	2.20284300	1.06799100
H	-8.52272100	2.85011100	0.71406400
H	-10.02454900	1.33336800	-0.57463400
H	-9.14733200	-0.77415000	-1.51271100
H	-7.25049100	-2.13318600	-1.96001000
H	-5.69528400	-1.68311500	-1.55961400
H	8.93987300	3.11517300	-0.63903000
H	8.42992600	3.01911100	1.06351800
H	7.44011900	3.91089300	-0.11272900

Cartesian coordinates of optimized geometries.

Zabadinostat in methanol

C	7.22758700	-1.74422700	-0.48010900
C	6.96807600	-0.27042100	-0.43236200
N	7.91505900	0.57706800	-0.01799700
N	7.36328700	1.81006900	-0.10094900
C	6.09110200	1.75845600	-0.56118700
C	5.78429900	0.42786000	-0.79044000
C	4.50108600	-0.13715000	-1.32487300
N	3.71177900	-0.87802500	-0.32711000
C	2.62505600	-1.62700400	-0.96722000
C	1.89838600	-2.51000800	0.04613900
C	1.30905500	-1.69809800	1.22729900
C	2.41805900	-0.78509100	1.78719400
C	3.17660100	0.00529400	0.71419000
C	-0.01053700	-1.01302000	0.87222800
C	-0.22495000	0.36815100	0.95139400
C	-1.46219500	0.92849400	0.64290800
C	-2.54149200	0.12564300	0.26464800
C	-2.34116200	-1.25981300	0.18965000
C	-1.09993700	-1.80998600	0.48495600

C	-3.84173600	0.80424700	-0.05558500
O	-3.87548400	1.97550800	-0.42963800
N	-4.96732800	0.04168000	0.10912500
C	-6.31749500	0.43496100	-0.07442100
C	-6.76322900	1.74064000	0.14559200
C	-8.11577100	2.05498600	0.02628900
C	-9.03332100	1.05823900	-0.30264800
C	-8.59559100	-0.24333400	-0.53136100
C	-7.23907300	-0.57405700	-0.43453200
N	-6.79192200	-1.89413700	-0.61199600
C	8.13677500	2.98347300	0.26904700
H	8.28644700	-1.94670300	-0.31066600
H	6.94743200	-2.16344900	-1.45100500
H	6.64895800	-2.27726100	0.27960300
H	5.50252500	2.65301000	-0.69574800
H	4.72423300	-0.83758800	-2.13619100
H	3.90430100	0.67917800	-1.77121200
H	3.05476600	-2.25335400	-1.75407200
H	1.90624700	-0.94213700	-1.45720400
H	2.61356300	-3.23393600	0.45012000
H	1.11773600	-3.08115200	-0.46205200
H	1.05674000	-2.42024300	2.01276300
H	3.13889400	-1.42344800	2.30800500
H	2.01872200	-0.09512500	2.53603800
H	4.00902800	0.53581100	1.18151900
H	2.52456600	0.77347200	0.25950700
H	0.57356700	1.02938100	1.25996400
H	-1.60124500	2.00095100	0.70196200
H	-3.13669600	-1.92582500	-0.12424900
H	-0.97930700	-2.88573100	0.41699400
H	-4.84214900	-0.89358800	0.47229900
H	-6.04553700	2.50134200	0.41485400
H	-8.44694100	3.07216200	0.19894200
H	-10.08811100	1.29176000	-0.39227800
H	-9.30571500	-1.01730600	-0.80328000
H	-7.51183400	-2.51958100	-0.95051400
H	-5.96403600	-1.98201800	-1.18975300
H	9.02340000	3.06535500	-0.36220200
H	8.44407600	2.91809700	1.31417100
H	7.51656000	3.86788000	0.13250600

Cartesian coordinates of optimized geometries.

[Zabadinostat - $Zn(H_2O)_2]^{2+}$ in gas phase

C	-8.28694300	-1.43474400	0.71185500
C	-8.04093800	-0.02471500	0.27534000
N	-8.95289000	0.63286200	-0.43825000

N	-8.44054700	1.86369400	-0.64699700
C	-7.21879700	2.00396600	-0.07950800
C	-6.91018500	0.79993500	0.53092200
C	-5.68093400	0.45919600	1.31545400
N	-4.87240900	-0.61238500	0.70698500
C	-3.84635500	-1.11998400	1.60650300
C	-3.21986600	-2.39492200	1.04006900
C	-2.60433800	-2.17344600	-0.37155200
C	-3.63244100	-1.44177100	-1.25795000
C	-4.30229000	-0.23947500	-0.57945600
C	-1.21613000	-1.56308800	-0.29881300
C	-0.87481900	-0.31604900	-0.85207200
C	0.41486200	0.18166600	-0.77267800
C	1.43489400	-0.56031100	-0.14364700
C	1.10910600	-1.82504500	0.38966500
C	-0.18449400	-2.30182900	0.31566000
C	2.79864900	-0.07745100	-0.01181600
N	3.06605700	1.23507600	-0.24249900
C	4.25550100	1.96960300	0.07520900
C	4.14500500	3.04509100	0.96310000
C	5.24192600	3.84837900	1.25185000
C	6.47353400	3.58635700	0.65439200
C	6.59842700	2.52431200	-0.23638700
C	5.49401100	1.72860800	-0.53642300
N	5.65595900	0.60569000	-1.46460000
C	-9.22459700	2.85714800	-1.36092100
H	-9.25798300	-1.76948100	0.34607200
H	-8.28744100	-1.51658400	1.80385300
H	-7.51368300	-2.10933900	0.33554000
H	-6.67117300	2.93267400	-0.13534500
H	-5.96414600	0.10640400	2.31264400
H	-5.07567800	1.37260800	1.46700100
H	-4.30683300	-1.34677000	2.57131800
H	-3.05854100	-0.36030000	1.79880400
H	-4.00365000	-3.15090400	0.94527200
H	-2.47501700	-2.78695000	1.73829800
H	-2.44309400	-3.17036600	-0.80200700
H	-4.41695100	-2.16228800	-1.50346700
H	-3.18751100	-1.13801900	-2.21099400
H	-5.10323700	0.13230300	-1.22074400
H	-3.58295300	0.59751800	-0.45454300
H	-1.62694800	0.27144400	-1.35923400
H	0.61698700	1.13524700	-1.25056300
H	1.87452200	-2.40869700	0.88435300
H	-0.41168000	-3.27262900	0.74097400
H	3.18717500	3.24585800	1.42991500

H	5.13370600	4.67693800	1.94058800
H	7.33066100	4.21378000	0.86531400
H	7.54960900	2.33768500	-0.72555200
H	6.50844500	0.73130100	-2.00970300
H	-10.11886700	3.11437600	-0.79031400
H	-9.52389700	2.46167100	-2.33217200
H	-8.61722200	3.75054100	-1.50368300
H	2.24531400	1.81704800	-0.35436600
H	4.88725300	0.58852900	-2.13838800
O	3.73738600	-0.87869100	0.32631600
Zn	5.54996400	-1.04619300	-0.25124600
O	5.79112700	-2.97088600	-0.89605600
H	5.06302700	-3.60868900	-0.86474900
H	6.50091300	-3.35617900	-1.42909000
O	6.99530900	-1.03722300	1.18205400
H	7.35231700	-1.82918700	1.60866300
H	7.17716500	-0.28249100	1.75992400

Cartesian coordinates of optimized geometries.

[Zabadinostat - Zn(H_2O)$_2$]$^{2+}$ in water

C	-8.43572600	-1.16732000	1.07466600
C	-8.03821100	0.13257600	0.44679300
N	-8.91199100	0.83573200	-0.28056900
N	-8.24288400	1.93899900	-0.68896500
C	-6.96867600	1.94852000	-0.23221600
C	-6.78318200	0.79440500	0.51018400
C	-5.54515100	0.36434500	1.24042900
N	-4.84810100	-0.77555500	0.62105300
C	-3.82450600	-1.31530300	1.52241000
C	-3.19377100	-2.58050600	0.94246800
C	-2.55053500	-2.33730700	-0.44662000
C	-3.58844200	-1.62778400	-1.33831100
C	-4.25410900	-0.41812300	-0.67140200
C	-1.18056200	-1.66945900	-0.34729800
C	-0.85189400	-0.46454600	-0.98098100
C	0.41768700	0.09348800	-0.86647200
C	1.41055600	-0.54766000	-0.11611900
C	1.10704100	-1.77612900	0.48944500
C	-0.16421600	-2.31653100	0.37688200
C	2.77022600	0.00778300	0.07067300
N	2.94274600	1.33090700	-0.13309900
C	4.10594100	2.11542900	0.13909500
C	3.95617700	3.22288900	0.97974200
C	5.03258500	4.05651900	1.25426500
C	6.27776100	3.78694600	0.68750200
C	6.43272900	2.69700300	-0.16073400
C	5.35034800	1.86713300	-0.45389900
N	5.54132200	0.73616300	-1.32992500

C	-8.91142200	2.94187700	-1.50103500
H	-9.51376300	-1.31395100	0.98936700
H	-8.16739400	-1.19225500	2.13493500
H	-7.93455900	-2.01337000	0.59584800
H	-6.29444400	2.76040100	-0.45815500
H	-5.81310000	0.05745700	2.25652900
H	-4.86627800	1.23038000	1.34354500
H	-4.29779000	-1.55091100	2.47966100
H	-3.03982400	-0.56174700	1.72825800
H	-3.97521400	-3.33849900	0.82862600
H	-2.46254300	-2.97954600	1.64917500
H	-2.35929400	-3.32350500	-0.88611500
H	-4.37026700	-2.35621600	-1.57526100
H	-3.14932300	-1.32545200	-2.29318300
H	-5.04339400	-0.03847600	-1.32370400
H	-3.52740600	0.40446200	-0.53936600
H	-1.58371800	0.05593500	-1.58288300
H	0.62230700	1.01485500	-1.39969700
H	1.87193500	-2.29232700	1.05505100
H	-0.37050900	-3.26602000	0.85774000
H	2.98651100	3.41507900	1.42344800
H	4.90046700	4.90609600	1.91251200
H	7.12604500	4.42693800	0.89660200
H	7.39598200	2.49270800	-0.61458500
H	6.40305600	0.84132400	-1.86181200
H	-9.76076000	3.36196600	-0.95927700
H	-9.26334600	2.49778900	-2.43365400
H	-8.20197200	3.73612300	-1.72731900
H	2.09744100	1.86992800	-0.26928900
H	4.78848100	0.66863100	-2.01517700
O	3.71010900	-0.74167700	0.43848900
Zn	5.56619400	-1.07375100	-0.28094400
O	5.69787100	-3.06053100	-0.88810100
H	4.86808400	-3.53230500	-1.04244900
H	6.26720600	-3.24191000	-1.64835200
O	7.14369400	-1.38773800	1.03565500
H	7.17937700	-2.26001400	1.45113300
H	7.29726600	-0.73467900	1.73155900

Cartesian coordinates of optimized geometries.

[Zabadinostat - $Zn(H_2O)_2]^{2+}$ in methanol

C	-8.43070800	-1.17730500	1.06539300
C	-8.04157600	0.12518200	0.43780600
N	-8.91855500	0.82069000	-0.29276200
N	-8.25722200	1.92899100	-0.69976500
C	-6.98438900	1.94906300	-0.23930100
C	-6.79200600	0.79710100	0.50457000
C	-5.55310400	0.37795700	1.23959900
N	-4.85007100	-0.76276200	0.62903900
C	-3.82558300	-1.29309600	1.53437400
C	-3.19322300	-2.56186000	0.96407200

C	-2.55116900	-2.32890500	-0.42747400
C	-3.58881200	-1.62536700	-1.32420800
C	-4.25714400	-0.41268400	-0.66546800
C	-1.18035200	-1.66254600	-0.33365300
C	-0.85203500	-0.45804400	-0.96841100
C	0.41893400	0.09770900	-0.85867900
C	1.41369500	-0.54581800	-0.11267700
C	1.11003500	-1.77356800	0.49464500
C	-0.16262300	-2.31124100	0.38721900
C	2.77506500	0.00582900	0.06784200
N	2.95089500	1.32908400	-0.13201500
C	4.11597200	2.11058200	0.14214100
C	3.96727000	3.21552500	0.98632600
C	5.04398800	4.04783200	1.26335600
C	6.28884300	3.77988000	0.69545000
C	6.44306900	2.69237500	-0.15604400
C	5.36053300	1.86357000	-0.45114500
N	5.55240600	0.73509000	-1.33206200
C	-8.93113700	2.92499300	-1.51570900
H	-9.50708700	-1.33290900	0.97557500
H	-8.16667200	-1.19884400	2.12683300
H	-7.92010200	-2.01928700	0.58951600
H	-6.31602700	2.76624000	-0.46370000
H	-5.82097100	0.07676700	2.25744900
H	-4.87855300	1.24807700	1.33775800
H	-4.29816800	-1.52225200	2.49351000
H	-3.04200400	-0.53661600	1.73432100
H	-3.97384400	-3.32151900	0.85661700
H	-2.46082900	-2.95446900	1.67324700
H	-2.36091200	-3.31848400	-0.85972000
H	-4.36961600	-2.35593300	-1.55755400
H	-3.14879900	-1.32832000	-2.28038600
H	-5.04746900	-0.03967200	-1.32029300
H	-3.53245400	0.41281500	-0.53917400
H	-1.58544000	0.06345200	-1.56748800
H	0.62334500	1.01892100	-1.39234500
H	1.87572900	-2.29090600	1.05810700
H	-0.36938300	-3.25998300	0.86937000
H	2.99789100	3.40692500	1.43093200
H	4.91213900	4.89542800	1.92414600
H	7.13725700	4.41920500	0.90582500
H	7.40616400	2.48982300	-0.61114400
H	6.41481900	0.84435600	-1.86224400
H	-9.78980000	3.33365000	-0.98003500
H	-9.27039300	2.47841300	-2.45186400
H	-8.22958300	3.72825500	-1.73483100

H	2.10627700	1.87077200	-0.26228900
H	4.80131100	0.67441400	-2.02010700
O	3.71560000	-0.74782100	0.42704800
Zn	5.56787700	-1.07557500	-0.29357100
O	5.67789500	-3.05919600	-0.89571000
H	4.84898300	-3.53402600	-1.04486100
H	6.25982600	-3.26913000	-1.63874800
O	7.14041400	-1.38796400	1.02172700
H	7.18995000	-2.26210200	1.43193100
H	7.30340600	-0.73657900	1.71706900

Cartesian coordinates of optimized geometries.
[Zabadinostat - Zn(H_2O)$_4$]$^{2+}$ in gas phase.

C	-8.75826500	-1.61243300	0.74431900
C	-8.56826000	-0.20886100	0.26159400
N	-9.49958600	0.38403000	-0.48314700
N	-9.03710000	1.62752900	-0.73038600
C	-7.82863600	1.83918600	-0.15617100
C	-7.47617100	0.67142400	0.49892100
C	-6.24176700	0.41036200	1.30599100
N	-5.38224300	-0.64591600	0.74323000
C	-4.34431800	-1.07282100	1.67204400
C	-3.64856900	-2.33291300	1.15598700
C	-3.02490900	-2.13077000	-0.25394400
C	-4.07402000	-1.47763000	-1.17689400
C	-4.81172100	-0.28795100	-0.54819400
C	-1.66675300	-1.45064100	-0.18562500
C	-1.38135500	-0.20277800	-0.76174000
C	-0.11611100	0.36174700	-0.67948100
C	0.92834200	-0.31387500	-0.02512500
C	0.66154100	-1.57883300	0.53320700
C	-0.60764000	-2.12262800	0.45624600
C	2.27835800	0.23934100	0.10211400
N	2.44480200	1.57898200	-0.03508800
C	3.63739200	2.33211400	0.19886900
C	3.58195500	3.38844600	1.11400600
C	4.69053700	4.19517600	1.33722700
C	5.87610900	3.95186700	0.64608400
C	5.94026700	2.91011000	-0.27284300
C	4.82467500	2.10926700	-0.50952700
N	4.91887200	1.02832800	-1.48461500
C	-9.85550000	2.56263400	-1.48306800
H	-9.71047000	-2.00099300	0.38215400
H	-8.76575100	-1.65688000	1.83846600
H	-7.95285100	-2.26512900	0.39814300
H	-7.31966500	2.78757800	-0.23986800

H	-6.52107800	0.08095500	2.31228000
H	-5.67763500	1.35376200	1.43126600
H	-4.80715900	-1.29097200	2.63784100
H	-3.59867500	-0.26879500	1.84958400
H	-4.39381200	-3.12910000	1.07976800
H	-2.89448400	-2.66378200	1.87583100
H	-2.81350600	-3.13216600	-0.65000800
H	-4.81970200	-2.24238100	-1.40979200
H	-3.62922700	-1.18240600	-2.13277900
H	-5.62085500	0.02120000	-1.21216500
H	-4.13741900	0.58776100	-0.44276700
H	-2.15488300	0.33588100	-1.29065400
H	0.05190800	1.31321100	-1.17403000
H	1.44785300	-2.10734400	1.05862100
H	-0.79263200	-3.09191500	0.90552700
H	2.65956900	3.56979000	1.65446400
H	4.62848200	5.00941500	2.04822100
H	6.74345500	4.57962100	0.80807000
H	6.85499700	2.73798100	-0.83130800
H	5.69090500	1.22108200	-2.12060100
H	-10.75902500	2.81139800	-0.92314700
H	-10.13965200	2.11731300	-2.43710100
H	-9.28011500	3.47044600	-1.66283800
H	1.59204600	2.12405400	-0.04172700
H	4.07649300	1.00530500	-2.06175300
O	3.27018100	-0.51210200	0.34109800
Zn	5.08720100	-0.80742100	-0.47919000
O	7.03129600	-1.39578000	-1.18284000
H	7.72958500	-1.63954900	-0.54006000
H	7.38702500	-1.49532000	-2.07377300
O	8.51750800	-1.94300100	1.11587400
H	8.77457000	-2.84747100	1.34552100
H	9.26579800	-1.38852000	1.37925800
O	5.90815200	-1.05693900	1.37335100
H	6.81105500	-1.37321400	1.57656800
H	5.39050500	-0.95672100	2.18110400
O	4.25402600	-2.47899000	-1.41057400
H	4.67434900	-3.26893900	-1.77288700
H	3.34034800	-2.69592500	-1.17894800

Cartesian coordinates of optimized geometries.
[Zabadinostat - $Zn(H_2O)_4]^{2+}$ in water.

C	-8.87470900	-1.55749300	0.77288000
C	-8.54956400	-0.14794100	0.38599900
N	-9.44848100	0.60923200	-0.25107500
N	-8.84573300	1.80482000	-0.44769000

C	-7.58902700	1.81952000	0.05509600
C	-7.34563500	0.57280500	0.60627600
C	-6.10248000	0.11360900	1.30973500
N	-5.33814900	-0.90801500	0.57569900
C	-4.29722300	-1.49321900	1.42723100
C	-3.58967100	-2.64743400	0.71840300
C	-2.94889300	-2.21342200	-0.62361700
C	-4.02142400	-1.47227300	-1.44603100
C	-4.75030200	-0.37712200	-0.65872700
C	-1.62171100	-1.47820700	-0.43823100
C	-1.34344400	-0.22617200	-1.00076700
C	-0.10291000	0.38395700	-0.84066200
C	0.91054900	-0.24570700	-0.10870600
C	0.65299700	-1.51066600	0.43961300
C	-0.58795900	-2.10701400	0.27727100
C	2.25308900	0.34973500	0.09583200
N	2.38317600	1.68278000	-0.08508900
C	3.55127100	2.46848600	0.14799000
C	3.44162700	3.56761400	1.00553100
C	4.53299100	4.39317000	1.24325500
C	5.75211100	4.12143900	0.62239100
C	5.86574400	3.03939800	-0.24238900
C	4.76781200	2.21709400	-0.50038200
N	4.91569600	1.09309200	-1.38849700
C	-9.56265400	2.88813000	-1.09926900
H	-9.94244700	-1.74617800	0.64859000
H	-8.60886300	-1.75226700	1.81585400
H	-8.32502700	-2.27802400	0.16050300
H	-6.96621800	2.69869500	-0.00775900
H	-6.37389800	-0.32767400	2.27433600
H	-5.47042400	0.99205100	1.53448900
H	-4.76798700	-1.86261200	2.34259800
H	-3.55779700	-0.72743900	1.73208900
H	-4.32498400	-3.43027700	0.50709800
H	-2.84588800	-3.08352000	1.38881900
H	-2.69439100	-3.13129600	-1.16745000
H	-4.76270600	-2.21232700	-1.76346100
H	-3.59875300	-1.04917200	-2.36134200
H	-5.55110100	0.03714900	-1.27493300
H	-4.06360900	0.45743700	-0.42479200
H	-2.09107400	0.28961100	-1.58715900
H	0.06420000	1.33807900	-1.32710800
H	1.43082000	-2.01538300	0.99842900
H	-0.75258200	-3.08766000	0.70855500
H	2.49214900	3.75875700	1.49176200
H	4.43403800	5.23649400	1.91536200
H	6.61246300	4.75403800	0.80351100
H	6.80849800	2.83368800	-0.73665900
H	5.72737000	1.22271100	-1.98862800
H	-10.43351700	3.17658700	-0.50752800
H	-9.88937400	2.57761000	-2.09287700
H	-8.89350600	3.74190900	-1.19231400
H	1.52620500	2.20914400	-0.19154000
H	4.10920600	1.00320500	-2.00590600

O	3.22319700	-0.36739100	0.43685700
Zn	5.11332400	-0.77153700	-0.34186800
O	7.09562300	-1.18469400	-0.96194800
H	7.61474900	-1.75599400	-0.35722200
H	7.30614300	-1.43534100	-1.86948400
O	8.04737900	-2.55512700	1.22036200
H	8.06191100	-3.52103100	1.24022200
H	8.86111600	-2.26783400	1.65514900
O	5.73032400	-1.13652400	1.59958700
H	6.50373600	-1.72970900	1.70912300
H	5.04714100	-1.39979000	2.22820000
O	4.31244800	-2.40372300	-1.42738400
H	4.87216300	-3.17500400	-1.58651600
H	3.46169600	-2.73593600	-1.11154900

Cartesian coordinates of optimized geometries.

[Zabadinostat - $Zn(H_2O)_4]^{2+}$ in methanol.

C	-8.86888800	-1.55768800	0.78792300
C	-8.55139500	-0.14975800	0.38920900
N	-9.45468500	0.59719200	-0.25334100
N	-8.85866000	1.79429400	-0.46021000
C	-7.60155500	1.82003000	0.04144900
C	-7.35115200	0.57929500	0.60272400
C	-6.10526600	0.13207300	1.30900200
N	-5.33877500	-0.89428800	0.58427200
C	-4.29629700	-1.47042900	1.43941100
C	-3.59112500	-2.63223500	0.74059300
C	-2.95342100	-2.21251800	-0.60763100
C	-4.02597800	-1.47652800	-1.43468100
C	-4.75381200	-0.37456800	-0.65580000
C	-1.62374200	-1.47985500	-0.43221000
C	-1.34552000	-0.22936500	-0.99824600
C	-0.10344300	0.37901800	-0.84382500
C	0.91189000	-0.25125700	-0.11480800
C	0.65465700	-1.51545900	0.43563100
C	-0.58791000	-2.10974800	0.27930100
C	2.25472900	0.34332300	0.08765200
N	2.38664400	1.67597600	-0.09482200
C	3.55362400	2.46189800	0.14527900
C	3.43910800	3.55885300	1.00481500
C	4.52861900	4.38483200	1.24952300
C	5.75074500	4.11579000	0.63371700
C	5.86936200	3.03583200	-0.23304700
C	4.77350700	2.21319300	-0.49775500
N	4.92614500	1.09119700	-1.38876000
C	-9.58218100	2.86766600	-1.12073300
H	-9.93495600	-1.75399700	0.66141600
H	-8.60576100	-1.74114900	1.83367200
H	-8.31214300	-2.28007900	0.18423700
H	-6.98360400	2.70210000	-0.02898200
H	-6.37359500	-0.30025300	2.27850600
H	-5.47608700	1.01516700	1.52386700

H	-4.76495900	-1.83048200	2.35954300
H	-3.55583900	-0.70179700	1.73489600
H	-4.32770300	-3.41640700	0.53920700
H	-2.84567000	-3.06195700	1.41333600
H	-2.70271500	-3.13628500	-1.14318800
H	-4.76816100	-2.21830300	-1.74566700
H	-3.60343400	-1.06106300	-2.35360700
H	-5.55608000	0.03338400	-1.27427300
H	-4.06716200	0.46269800	-0.43087500
H	-2.09486500	0.28658800	-1.58228200
H	0.06290000	1.33256800	-1.33174800
H	1.43358300	-2.02038200	0.99266500
H	-0.75299200	-3.08928400	0.71297700
H	2.48731300	3.74801900	1.48721900
H	4.42572600	5.22655200	1.92299500
H	6.60956300	4.74892800	0.81998800
H	6.81457600	2.83269700	-0.72368300
H	5.74318300	1.22109600	-1.98162100
H	-10.45547100	3.15471500	-0.53192700
H	-9.90619000	2.54719100	-2.11207800
H	-8.91881600	3.72529300	-1.21994400
H	1.52998500	2.20273200	-0.20216700
H	4.12458700	1.00601700	-2.01321700
O	3.22440600	-0.37444700	0.42989100
Zn	5.11278700	-0.77421000	-0.34614800
O	7.09477600	-1.18809900	-0.96859000
H	7.62697000	-1.73585700	-0.35334800
H	7.30772100	-1.45412900	-1.87109400
O	8.07816900	-2.51478000	1.22731300
H	8.12204500	-3.47962700	1.25503300
H	8.88258300	-2.19975200	1.66040600
O	5.73273900	-1.14168200	1.59015100
H	6.51425000	-1.72160400	1.70999300
H	5.05794600	-1.38827600	2.23413100
O	4.31393300	-2.40164600	-1.43534600
H	4.87193600	-3.16887200	-1.61754000
H	3.46353900	-2.73965300	-1.12469800

Cartesian coordinates of optimized geometries.

Chidamide in gas phase.

C	-8.10065300	2.26184800	-0.85589700
C	-7.19446800	1.21659900	-0.94835000
C	-6.10392100	1.17483000	-0.06665700
C	-6.00729900	2.21929200	0.86741100
N	-6.87167900	3.22871500	0.96770600
C	-7.89996200	3.24400600	0.11615600
C	-5.08279300	0.12388700	-0.06278100
C	-5.00368800	-0.92632600	-0.89309600
C	-3.91198600	-1.93969600	-0.91422900
O	-3.59840800	-2.48936000	-1.96433200

N	-3.29650100	-2.21636900	0.27549800
C	-2.17523500	-3.14564000	0.38521000
C	-0.83001200	-2.45571600	0.50899900
C	-0.08262700	-2.54225800	1.68624600
C	1.14612500	-1.89950800	1.80172900
C	1.66343100	-1.16745400	0.72991200
C	0.91250200	-1.07160600	-0.45005000
C	-0.31869700	-1.70805300	-0.55830200
C	2.99547400	-0.50375200	0.92329400
O	3.40452200	-0.20258400	2.04174800
N	3.71076600	-0.26586700	-0.21876800
C	5.02059600	0.28751100	-0.32762800
C	5.39159000	1.49843200	0.30394800
C	6.67604100	2.00717100	0.04434300
C	7.52751700	1.34555700	-0.81692700
C	7.18413400	0.15905800	-1.44530200
C	5.92191300	-0.35812000	-1.17668500
N	4.49999000	2.21057800	1.09206700
H	-8.95223400	2.32167800	-1.52321300
H	-7.33226400	0.44412100	-1.69587400
H	-5.17795400	2.23158900	1.57143500
H	-8.59266800	4.07479600	0.21311800
H	-3.63019200	-1.76717800	1.11323600
H	-2.20142700	-3.76541500	-0.51273300
H	-2.34147800	-3.79513900	1.24831600
H	-0.46415200	-3.11821000	2.52359100
H	1.71935000	-1.95372500	2.71891800
H	1.26425400	-0.47136000	-1.28220200
H	-0.89305400	-1.62265600	-1.47425200
H	3.36020300	-0.69638000	-1.06107000
H	7.00333100	2.92839800	0.51180100
H	5.62100200	-1.29144100	-1.64084300
H	3.94727200	1.62782300	1.71594600
H	4.91145200	3.00018100	1.56911700
H	-5.71171000	-1.06443700	-1.70267700
H	-4.30929600	0.25805000	0.69095500
H	7.88042100	-0.33870900	-2.10633100
F	8.75381200	1.87931100	-1.04668200

Cartesian coordinates of optimized geometries.

Chidamide in water.

C	-8.15993700	2.37101600	-0.46401500
C	-7.30499900	1.29058500	-0.62344900
C	-6.06403300	1.29565900	0.03220900
C	-5.76845000	2.41795300	0.82264200
N	-6.58320400	3.46389400	0.98422000
C	-7.75968600	3.43393200	0.34694700
C	-5.07561200	0.21696100	-0.05478600
C	-5.17392600	-0.90124000	-0.78991700
C	-4.12614600	-1.95063100	-0.91061000
O	-4.04031700	-2.61916700	-1.94650100
N	-3.29240100	-2.12361800	0.14865900

C	-2.21203200	-3.09868900	0.17216400
C	-0.83270300	-2.48358400	0.32341300
C	0.13841900	-3.13243800	1.09339000
C	1.42123100	-2.60935700	1.21090700
C	1.76867000	-1.42489700	0.55142800
C	0.79423700	-0.76589700	-0.20997800
C	-0.49017400	-1.28992400	-0.31894800
C	3.16529800	-0.91146100	0.72060000
O	3.84683600	-1.20446900	1.70825600
N	3.63205300	-0.10519800	-0.27525500
C	4.93508900	0.46407300	-0.37845500
C	5.50837800	1.23417200	0.66169600
C	6.76011800	1.83095600	0.42763100
C	7.37878900	1.67651300	-0.79617500
C	6.83485200	0.93397500	-1.83154100
C	5.60601900	0.32549300	-1.59576500
N	4.83094700	1.47088500	1.85148100
H	-9.12411400	2.39616900	-0.95664000
H	-7.60077700	0.45637500	-1.24821000
H	-4.81866400	2.46362900	1.34940100
H	-8.40934700	4.29199200	0.48895700
H	-3.48945800	-1.62427700	1.00295700
H	-2.27047100	-3.65160100	-0.76779400
H	-2.38388300	-3.81350400	0.98116100
H	-0.11207400	-4.05325700	1.60938400
H	2.16471500	-3.11338100	1.81556100
H	1.01403800	0.17620800	-0.69894000
H	-1.23375200	-0.75749400	-0.90016700
H	3.07733200	-0.06072800	-1.11803500
H	7.23607700	2.42179900	1.20114900
H	5.14753000	-0.26876000	-2.37814500
H	4.35748600	0.65245500	2.22268700
H	5.38947800	1.93038800	2.55843500
H	-6.02678000	-1.08207600	-1.43452200
H	-4.17654000	0.38720600	0.53255500
H	7.35178500	0.83058700	-2.77603700
F	8.58700900	2.28073300	-0.98643000

Cartesian coordinates of optimized geometries.
Chidamide in methanol.

C	-8.15897500	2.37273100	-0.46408400
C	-7.30536300	1.29120800	-0.62293800
C	-6.06290400	1.29681300	0.02984100
C	-5.76451600	2.42096900	0.81658800
N	-6.57796900	3.46786200	0.97772900
C	-7.75592100	3.43725200	0.34344300
C	-5.07566100	0.21702200	-0.05668600
C	-5.17764200	-0.90439200	-0.78636200
C	-4.13102100	-1.95498100	-0.90724900
O	-4.05078900	-2.62857900	-1.93987900
N	-3.29127000	-2.12235700	0.14868500
C	-2.21150100	-3.09824400	0.17146000

C	-0.83243500	-2.48308200	0.32368100
C	0.13534600	-3.12570200	1.10292300
C	1.41767000	-2.60160100	1.22189100
C	1.76784200	-1.42253500	0.55444500
C	0.79669500	-0.76964200	-0.21637600
C	-0.48713500	-1.29459000	-0.32679500
C	3.16377700	-0.90769900	0.72560300
O	3.84050300	-1.19195500	1.71881600
N	3.63480200	-0.11038900	-0.27571000
C	4.93755200	0.45953200	-0.37876200
C	5.50667300	1.23800000	0.65739400
C	6.75832800	1.83464900	0.42271700
C	7.38115100	1.67190900	-0.79786700
C	6.84135300	0.92092400	-1.82931200
C	5.61257500	0.31267500	-1.59281700
N	4.82553400	1.48247500	1.84338200
H	-9.12422900	2.39757100	-0.95464900
H	-7.60317100	0.45582800	-1.24518300
H	-4.81344400	2.46731200	1.34101400
H	-8.40441500	4.29628800	0.48491600
H	-3.48475900	-1.62002700	1.00201600
H	-2.27042500	-3.64966600	-0.76929200
H	-2.38350500	-3.81419200	0.97952300
H	-0.11734000	-4.04248200	1.62504500
H	2.15870500	-3.10031500	1.83390200
H	1.01868700	0.16865000	-0.71167400
H	-1.22816000	-0.76722100	-0.91590000
H	3.08375900	-0.07433800	-1.12125300
H	7.23109600	2.43195000	1.19323000
H	5.15738100	-0.28810800	-2.37218800
H	4.35155300	0.66618500	2.21868800
H	5.38176500	1.94704800	2.54878900
H	-6.03324000	-1.08765100	-1.42661500
H	-4.17389300	0.38937300	0.52595200
H	7.36154300	0.81104900	-2.77126800
F	8.58904200	2.27628700	-0.98884300

Cartesian coordinates of optimized geometries.

[Chidamide - Zn(H_2O)$_2$]$^{2+}$ in gas phase.

C	9.23419600	1.24756000	-1.61196000
C	8.15442100	0.48309500	-1.20211800
C	7.14667400	1.08235100	-0.42795900
C	7.31069000	2.44288600	-0.11417800
N	8.34095300	3.18838600	-0.50541900
C	9.28433800	2.59496300	-1.24160000
C	5.96043800	0.39669900	0.06472300
C	5.58792500	-0.87771500	-0.15083000
C	4.34708500	-1.40041000	0.44944100
O	3.56398300	-0.74134800	1.12942500
N	4.07171200	-2.72834400	0.17978800
C	3.00775000	-3.39666400	0.90858800
C	1.64703300	-2.77651300	0.66890200

C	0.75026600	-2.62101000	1.73386700
C	-0.48516300	-2.03201400	1.54068400
C	-0.87511700	-1.60239800	0.25639900
C	0.01044600	-1.79962600	-0.82494100
C	1.25025100	-2.37236800	-0.61510800
C	-2.16329800	-0.94834800	0.10841000
O	-2.77615500	-0.48663900	1.13369600
N	-2.71761800	-0.81925500	-1.12317000
C	-4.04496200	-0.37870500	-1.43470900
C	-4.48957700	0.92795700	-1.19087700
C	-5.77363500	1.32319700	-1.55384900
C	-6.59940000	0.40758400	-2.19381900
C	-6.18227100	-0.88660300	-2.47569300
C	-4.90509600	-1.26791900	-2.08951500
N	-3.62309900	1.89069000	-0.50673200
H	10.03093800	0.81733000	-2.20633200
H	8.09848600	-0.56452500	-1.47506200
H	6.55837200	2.94626900	0.48927300
H	10.11787800	3.21944800	-1.54790300
H	4.79419100	-3.30127000	-0.22999400
H	3.20909600	-3.39662800	1.98480400
H	2.98444400	-4.44073200	0.58058400
H	1.04150800	-2.93966600	2.72795200
H	-1.16673800	-1.91334900	2.37302600
H	-0.23098100	-1.45663500	-1.82587900
H	1.94236300	-2.47647300	-1.44162900
H	-2.26099600	-1.34356800	-1.85933000
H	-6.13382700	2.33192700	-1.38398800
H	-4.56529600	-2.27709100	-2.29231000
H	-2.68782400	1.87200500	-0.91985900
H	-3.97688700	2.83550800	-0.65537200
H	-6.84925900	-1.57260900	-2.98222100
F	-7.82204800	0.79805800	-2.54676300
Zn	-3.49818200	1.25213300	1.45032900
O	-2.25449600	2.18819200	2.78133700
H	-1.48209300	1.72971800	3.14486600
H	-2.17531400	3.12565100	3.00789900
O	-5.23286100	1.31987400	2.49976500
H	-5.29952100	1.56286300	3.43436900
H	-6.02924900	0.82053700	2.26841800
H	5.29471700	0.99273900	0.68483600
H	6.18453400	-1.55492900	-0.75332900

Cartesian coordinates of optimized geometries.

[Chidamide - $Zn(H_2O)_2]^{2+}$ in water.

C	-9.44131700	2.32768200	-0.25916200
C	-8.34412000	1.57152600	0.12511800
C	-7.83089800	0.60876600	-0.75859600
C	-8.47965200	0.47436500	-1.99719300
N	-9.53668800	1.19614500	-2.37843400
C	-10.00472700	2.10737400	-1.51694300

C	-6.68087500	-0.24828000	-0.47299700
C	-5.93252100	-0.25928800	0.63997200
C	-4.78909600	-1.19771000	0.77927900
O	-4.47134100	-2.01590800	-0.08793800
N	-4.10837500	-1.08836100	1.95434400
C	-2.98965400	-1.94566800	2.29732600
C	-1.62337800	-1.45176300	1.84579300
C	-0.52302100	-2.31061900	1.96861600
C	0.74563600	-1.90080100	1.58664600
C	0.94839700	-0.60665100	1.08460700
C	-0.14963400	0.25248500	0.95455500
C	-1.42133300	-0.17126800	1.32889100
C	2.32543000	-0.21220300	0.69868000
O	3.16550400	-1.08684600	0.38145200
N	2.62349600	1.10545700	0.72406800
C	3.90081200	1.70957500	0.52697300
C	4.64485800	1.54236800	-0.64828000
C	5.87643400	2.18006900	-0.79020500
C	6.32338700	3.00132700	0.22799500
C	5.59868800	3.21540500	1.38954900
C	4.38422000	2.55804900	1.52789200
N	4.17811400	0.67791600	-1.70228500
H	-9.86001800	3.07797900	0.40014200
H	-7.89472800	1.72825800	1.09843000
H	-8.11589600	-0.25974100	-2.71149600
H	-10.86413100	2.68506100	-1.84287000
H	-4.41296000	-0.40375000	2.62996000
H	-3.17008500	-2.93228400	1.86602200
H	-2.98117000	-2.06579600	3.38384000
H	-0.66255200	-3.31077500	2.36498100
H	1.58910400	-2.57243000	1.68310700
H	-0.03859200	1.24219000	0.52658700
H	-2.26160100	0.50043100	1.20589300
H	1.92726200	1.70902100	1.14192600
H	6.47243900	2.05292200	-1.68516200
H	3.79957900	2.69443700	2.42938300
H	3.18138900	0.81065000	-1.87459900
H	4.65292300	0.89002600	-2.57773700
H	5.97892700	3.86851600	2.16357300
F	7.51524200	3.62476600	0.07318400
Zn	4.43159500	-1.35287000	-1.23191900
O	3.29982500	-3.05982900	-1.69144200
H	2.39843200	-3.04271200	-1.34305300
H	3.24268800	-3.35600700	-2.60981400
O	6.09524800	-2.57202000	-1.31061600
H	5.99519100	-3.43209400	-0.88036200
H	6.94604500	-2.20952900	-1.02941400
H	-6.41011000	-0.95139400	-1.25662200
H	-6.13269500	0.41049600	1.46962600

Cartesian coordinates of optimized geometries.

[Chidamide - Zn(H_2O)$_4$]$^{2+}$ in gas phase.

C	-7.35638400	2.51601400	1.30896400
C	-6.53687300	1.41504200	1.12606300
C	-5.37605600	1.55097600	0.34462900
C	-5.12734100	2.81947500	-0.20782600
N	-5.90269300	3.88459700	-0.03547000
C	-6.99991000	3.72984300	0.71164900
C	-4.42660800	0.48434600	0.07149700
C	-4.45380600	-0.78978900	0.50426600
C	-3.40802600	-1.74852400	0.11621500
O	-2.39525300	-1.44616400	-0.54617100
N	-3.60797500	-3.02461800	0.54793300
C	-2.75918100	-4.17577200	0.21845900
C	-1.29044600	-3.85301400	0.38592200
C	-0.41133800	-3.99751300	-0.69218400
C	0.83615200	-3.40023900	-0.65873600
C	1.23962000	-2.68035300	0.47615100
C	0.43283600	-2.70713400	1.62670500
C	-0.82097400	-3.29666200	1.57872100
C	2.30880200	-1.68269500	0.33551900
O	2.53906400	-1.14559600	-0.78414400
N	2.94778600	-1.23212600	1.43923000
C	3.73678700	-0.04386200	1.48211700
C	3.18698500	1.19133700	1.10238200
C	3.96829300	2.34550300	1.16439800
C	5.27238800	2.25373800	1.63805000
C	5.82813600	1.04949900	2.04811100
C	5.04815300	-0.09600900	1.96153000
N	1.83018700	1.27885700	0.60352600
H	-8.26025500	2.44764200	1.90153000
H	-6.79744200	0.46589900	1.58006100
H	-4.24437800	2.96775100	-0.82728700
H	-7.62229600	4.60990900	0.83866000
H	-4.47970300	-3.22406200	1.01597100
H	-2.95010400	-4.50690500	-0.80628200
H	-3.05756300	-4.98890500	0.88519800
H	-0.74375300	-4.49915700	-1.59393600
H	1.48319400	-3.43817600	-1.52600000
H	0.72807100	-2.18589400	2.53055900
H	-1.47400900	-3.23324600	2.44136800
H	2.91083800	-1.80285500	2.27465100
H	3.56793800	3.32007900	0.90647800
H	5.46344600	-1.05175900	2.25950100
H	1.20440200	0.72121500	1.18492400
H	1.49794700	2.23928100	0.66883200
H	6.84424500	1.01902800	2.41970800
F	5.99907400	3.37117500	1.70434800
Zn	1.60866300	0.54573500	-1.46096100
O	-0.41115800	-0.07961000	-1.61225700
H	-0.52136700	-0.54502400	-2.45103000
H	-1.11760800	-0.48455000	-1.03192300
O	3.49487600	1.53946900	-2.03029000
H	4.00110700	1.16348700	-2.76157500

H	4.13513700	1.74551200	-1.33357100
H	-3.59397400	0.76109300	-0.57120200
H	-5.25759300	-1.15481800	1.13447400
O	1.78916300	-0.38078600	-3.47340700
H	2.20515400	-1.25170800	-3.51529800
H	1.68872400	-0.05835500	-4.37795700
O	0.82136000	2.38511000	-2.23334200
H	-0.11529500	2.52281000	-2.42195500
H	1.33077100	3.09748000	-2.63943600

Cartesian coordinates of optimized geometries.

[Chidamide - $Zn(H_2O)_4]^{2+}$ in water.

C	-7.35650200	-2.54952000	-1.49893200
C	-6.55960100	-1.44717500	-1.22942400
C	-5.49616400	-1.57546400	-0.32151300
C	-5.30986000	-2.83835000	0.26438400
N	-6.07029100	-3.90688800	0.01284600
C	-7.07649900	-3.75688400	-0.85701700
C	-4.58401400	-0.49268400	0.04298900
C	-4.59748100	0.76697000	-0.41859900
C	-3.59423000	1.75876200	0.03732400
O	-2.69984400	1.49410200	0.85586000
N	-3.72213000	2.98955900	-0.51918000
C	-2.82191100	4.11280200	-0.24807500
C	-1.36972400	3.73165900	-0.45287400
C	-0.44268900	3.91532600	0.57659600
C	0.80421900	3.31388500	0.51184100
C	1.15401900	2.54425900	-0.60558800
C	0.28308100	2.48597800	-1.70272200
C	-0.96881500	3.08152800	-1.62303300
C	2.27732500	1.59508000	-0.47362400
O	2.58149300	1.13761900	0.65865600
N	2.88896500	1.12599900	-1.57811600
C	3.78383700	0.01661700	-1.56745900
C	3.37230200	-1.23274900	-1.07038700
C	4.27528300	-2.29765000	-1.06689700
C	5.54597100	-2.10218400	-1.58174600
C	5.96598000	-0.89221400	-2.10820900
C	5.06775200	0.16815300	-2.09130000
N	2.05347000	-1.42398600	-0.54149900
H	-8.18400400	-2.48287000	-2.19442000
H	-6.76253300	-0.50201500	-1.71863800
H	-4.49831300	-2.98170500	0.97307100
H	-7.68436700	-4.63535400	-1.05011300
H	-4.47899400	3.14364400	-1.16820500
H	-2.96584500	4.46983400	0.77483000
H	-3.11690200	4.91894000	-0.92257100
H	-0.73279800	4.45151400	1.47316900
H	1.48403100	3.37561400	1.35191700
H	0.53009600	1.89721300	-2.57787200
H	-1.66979500	2.95727300	-2.44034400

H	2.75973100	1.62131300	-2.45138000
H	3.98623900	-3.27626600	-0.70273900
H	5.36660100	1.13520900	-2.47663400
H	1.35082000	-1.01815300	-1.15867800
H	1.83475200	-2.41525500	-0.47110600
H	6.96543000	-0.78227700	-2.50723700
F	6.40329500	-3.15054300	-1.58037000
Zn	1.62868800	-0.52364200	1.46274500
O	-0.37726200	0.24368600	1.85694500
H	-0.27826000	0.85145100	2.60104400
H	-1.11574000	0.59131600	1.31468200
O	3.53388100	-1.45130000	2.16068600
H	3.96851500	-1.00972000	2.90124300
H	4.23648000	-1.66635800	1.53293200
H	-3.81287200	-0.74874300	0.76500600
H	-5.33518800	1.10142600	-1.13970600
O	1.82986800	0.54086200	3.40191000
H	2.41160800	1.31134700	3.43069400
H	1.84424400	0.14030500	4.28052800
O	0.85809100	-2.29849400	2.27311700
H	-0.00696600	-2.23759500	2.69896800
H	1.44014900	-2.80741300	2.85240000

Cartesian coordinates of optimized geometries.

[Chidamide - Zn(H_2O)$_4$]$^{2+}$ in methanol.

C	-7.30543000	-2.59853800	-1.43486800
C	-6.50859000	-1.48782100	-1.20209200
C	-5.44070500	-1.58833800	-0.29594900
C	-5.24970200	-2.83352100	0.32541700
N	-6.00983900	-3.90973100	0.10944700
C	-7.02076500	-3.78620900	-0.75899500
C	-4.52901700	-0.49476800	0.03473100
C	-4.55074200	0.75605800	-0.45045400
C	-3.54756800	1.75761100	-0.01637100
O	-2.63633700	1.50131200	0.78633900
N	-3.69395600	2.98698400	-0.57152000
C	-2.80831100	4.12343400	-0.30359600
C	-1.35086500	3.75829800	-0.49785800
C	-0.43161400	3.95211700	0.53689600
C	0.81918800	3.35755600	0.48325600
C	1.18035400	2.58356700	-0.62767000
C	0.31781800	2.52016500	-1.73188800
C	-0.93679900	3.11027100	-1.66442200
C	2.29671100	1.62826700	-0.48090000
O	2.60650800	1.19316600	0.65812500
N	2.88737400	1.12407000	-1.58288500
C	3.72647000	-0.02759700	-1.57486900
C	3.26275600	-1.24876300	-1.05395700
C	4.10268500	-2.36370400	-1.06758500
C	5.36620200	-2.24387800	-1.62202200
C	5.83709000	-1.06243600	-2.17046900

C	5.00098300	0.04714900	-2.13731400
N	1.95133400	-1.36004900	-0.48008000
H	-8.13655700	-2.55357600	-2.12774900
H	-6.71465800	-0.55765000	-1.71809300
H	-4.43446200	-2.95501500	1.03400200
H	-7.62864200	-4.67058900	-0.92280400
H	-4.46755100	3.13745500	-1.20153400
H	-2.96251700	4.48689600	0.71557900
H	-3.10893300	4.92046100	-0.98639700
H	-0.73141000	4.48933000	1.42962200
H	1.49286100	3.42584100	1.32774100
H	0.57210100	1.92957100	-2.60365700
H	-1.63062800	2.98058200	-2.48695600
H	2.75848600	1.60627300	-2.46342200
H	3.77049000	-3.32021300	-0.68172300
H	5.34162100	0.99199100	-2.54247700
H	1.25373400	-0.92087100	-1.08002600
H	1.68116400	-2.33735200	-0.39507800
H	6.82864200	-1.01201300	-2.60002700
F	6.16291500	-3.33813100	-1.63675100
Zn	1.68832700	-0.45995800	1.51815300
O	-0.35741300	0.21155100	1.78250400
H	-0.34192800	0.78254500	2.56177700
H	-1.07715900	0.56091100	1.21535300
O	3.60023500	-1.32874600	2.18096700
H	4.04248200	-0.88070800	2.91333600
H	4.29195000	-1.52430600	1.53495200
H	-3.75048400	-0.73290900	0.75500100
H	-5.29724700	1.07635900	-1.16891200
O	1.78541100	0.59326500	3.43808100
H	2.31387900	1.40014200	3.49122200
H	1.81322500	0.17656200	4.30904300
O	0.90313600	-2.25784200	2.30793400
H	0.01695800	-2.20974700	2.68964800
H	1.45092200	-2.79213200	2.89729300

Cartesian coordinates of optimized geometries.

o-ABA in gas phase.

C	4.72350700	0.56473300	-0.17996600
C	4.40127900	-0.79087200	-0.23971800
C	3.07811100	-1.20037100	-0.11365700
C	2.05544800	-0.26082500	0.06132000
C	2.38861200	1.09804800	0.12750100
C	3.71473200	1.50753700	0.00886000
C	0.65296600	-0.79803800	0.19495700
O	0.44974900	-1.97307800	0.46157400
N	-0.35179600	0.11798900	-0.00660300
C	-1.74734400	-0.09110100	-0.00462300
C	-2.35324600	-1.34984100	-0.05614200

C	-3.74085500	-1.45106200	-0.13852000
C	-4.53250500	-0.30706100	-0.17384300
C	-3.93328800	0.94839600	-0.09225700
C	-2.54888300	1.07195700	0.00700400
N	-1.90105000	2.33744200	0.05875500
H	5.18238000	-1.52906100	-0.38212700
H	2.81119700	-2.24924700	-0.14454600
H	1.62916600	1.85352500	0.29656700
H	3.95952000	2.56183800	0.06994300
H	-0.08829800	1.04090500	-0.32433800
H	-1.73122800	-2.23147300	-0.03844500
H	-4.19807800	-2.43264800	-0.18234200
H	-5.61095900	-0.38579200	-0.24679600
H	-4.54459300	1.84558600	-0.08945100
H	-2.54716500	3.10497400	-0.07499800
H	-1.39629600	2.48421100	0.92776400
H	5.75507000	0.88479100	-0.27438000

Cartesian coordinates of optimized geometries.

o-ABA in water.

C	4.75637400	0.41010600	-0.29145100
C	4.37256300	-0.89874200	0.00387200
C	3.02956900	-1.20270000	0.20627700
C	2.05090600	-0.20643700	0.10009300
C	2.44418300	1.10615300	-0.19254100
C	3.79048900	1.41167200	-0.38342100
C	0.62137000	-0.60915600	0.33935200
O	0.34208800	-1.58873700	1.02695600
N	-0.32935100	0.17179100	-0.25832500
C	-1.73738100	-0.05078700	-0.20340500
C	-2.28622400	-1.29456400	-0.51626700
C	-3.66486400	-1.48818100	-0.49746200
C	-4.50074500	-0.41685600	-0.17550100
C	-3.96344000	0.82706400	0.13498000
C	-2.57431100	1.03208600	0.13730700
N	-2.03171400	2.28961100	0.40304100
H	5.11931400	-1.68090900	0.07777400
H	2.72391000	-2.21390000	0.44393400
H	1.71802800	1.90877700	-0.24566500
H	4.08339100	2.43288700	-0.59761800
H	-0.01231200	0.91881700	-0.86098300
H	-1.62139400	-2.10811000	-0.77631200
H	-4.08010400	-2.45899300	-0.73963400
H	-5.57667300	-0.55019100	-0.16136500
H	-4.61684900	1.65445900	0.39143000
H	-2.65968000	2.92914600	0.87066000
H	-1.12041800	2.28086000	0.84205500
H	5.80260400	0.64928000	-0.44392800

Cartesian coordinates of optimized geometries.

o-ABA in methanol.

C	4.74839200	0.45713600	-0.27626500
C	4.38328300	-0.87457900	-0.07466100
C	3.04624900	-1.20974200	0.11702600
C	2.05428400	-0.22117000	0.09418300
C	2.42926600	1.11436300	-0.10392800
C	3.76962600	1.45030600	-0.28463000
C	0.63273500	-0.66137400	0.31632600
O	0.37532800	-1.70066800	0.91945300
N	-0.33419400	0.15706700	-0.20066900
C	-1.73986000	-0.06236600	-0.15568800
C	-2.29894200	-1.31481000	-0.41373500
C	-3.68106100	-1.48645500	-0.41975000
C	-4.51078000	-0.39002400	-0.18073800
C	-3.96216000	0.86140400	0.07861100
C	-2.57229500	1.04612700	0.10853700
N	-2.01277700	2.30997200	0.32317300
H	5.14004100	-1.65055300	-0.06559900
H	2.75492200	-2.23942000	0.28215000
H	1.69402200	1.91047500	-0.09063700
H	4.04787500	2.48822500	-0.42550400
H	-0.02925900	0.96367800	-0.72805100
H	-1.64226400	-2.15066200	-0.61269700
H	-4.10324100	-2.46410100	-0.61896200
H	-5.58841400	-0.50860100	-0.18883400
H	-4.60875400	1.71034700	0.27508100
H	-2.66412900	2.99371700	0.68500100
H	-1.15708000	2.30992100	0.86384000
H	5.79003500	0.72014200	-0.42067300

Cartesian coordinates of optimized geometries.

[o-ABA- Zn(H_2O)$_2$]$^{2+}$ in gas phase.

C	2.73617100	-3.15080500	-0.82397700
C	1.40196100	-2.79287800	-0.67340700
C	1.03967900	-1.66831900	0.07616700
C	2.04961000	-0.91668000	0.69392900
C	3.38885800	-1.26821400	0.53154100
C	3.73392100	-2.38517000	-0.22326300
H	2.99432900	-4.02602100	-1.40700200
H	0.62501500	-3.38641300	-1.14230900
H	4.16382700	-0.68943300	1.02485200
H	4.77564000	-2.66316100	-0.32524800
N	1.72062000	0.27491100	1.48255500
H	2.52229800	0.53371800	2.05750900
N	-0.36143100	-1.41228000	0.25170900
C	-1.07483300	-0.31830700	-0.10012300
O	-0.49571800	0.75081400	-0.48241300
H	-0.91582300	-2.24837800	0.39286600
Zn	1.09732000	1.65028400	0.09247500
H	0.96054700	0.07296800	2.13577400

C	-2.53761900	-0.38347600	-0.05115300
C	-3.27102900	0.53843100	-0.82011500
C	-3.22194100	-1.34282500	0.72094000
C	-4.65801700	0.48511300	-0.83243400
H	-2.74760200	1.26550200	-1.42707400
C	-4.60880700	-1.37821700	0.71457300
H	-2.69164000	-2.03454400	1.36729100
C	-5.32772200	-0.46999500	-0.06623400
H	-5.21946900	1.18216900	-1.44229600
H	-5.13207600	-2.10619100	1.32205900
O	2.50991700	2.05544300	-1.31006200
H	3.01198900	1.38266400	-1.79185100
H	2.57104700	2.88334200	-1.80801700
O	0.54732300	3.56652500	0.52880800
H	-0.36422200	3.87317100	0.41349900
H	1.02929200	4.24644100	1.02096900
H	-6.41067000	-0.50697400	-0.07443500

Cartesian coordinates of optimized geometries.

[o-ABA-Zn(H_2O)$_2$]$^{2+}$in water.

C	2.90481900	-3.19347600	-0.83830600
C	1.55429500	-2.89398200	-0.71565100
C	1.13462400	-1.77184400	0.00621100
C	2.09029500	-0.96217300	0.63253200
C	3.44634600	-1.25623100	0.49016900
C	3.85451700	-2.36622600	-0.23937100
H	3.21302900	-4.06371700	-1.40411600
H	0.80874400	-3.52415300	-1.18622900
H	4.18050900	-0.61773500	0.96859500
H	4.91073100	-2.58740300	-0.33079900
N	1.69420200	0.19947800	1.39208900
H	2.45112100	0.49829300	2.00381900
N	-0.27139300	-1.55384700	0.13375700
C	-0.97202400	-0.44396000	-0.17850600
O	-0.42506900	0.62149900	-0.54998600
H	-0.81710300	-2.39581100	0.26471900
Zn	1.11583100	1.75983200	0.13989000
H	0.89893400	-0.00545800	1.99708700
C	-2.45327800	-0.52260900	-0.09264700
C	-3.20256100	0.35657400	-0.88672700
C	-3.11289900	-1.42785100	0.75135300
C	-4.59200700	0.31235800	-0.85581800
H	-2.68888900	1.05736700	-1.53204300
C	-4.50374000	-1.45828800	0.78735900
H	-2.55806900	-2.08489700	1.41110500
C	-5.24446000	-0.59437400	-0.01945300
H	-5.16564700	0.98445600	-1.48276000
H	-5.00748200	-2.15060800	1.45099400
O	2.27318700	3.01602100	-1.00224700
H	2.98575000	2.63956200	-1.53610200

H	1.78187200	3.62623200	-1.56899000
O	-0.15666200	3.37578600	0.46550600
H	-1.09687000	3.16033800	0.52971300
H	0.04102900	3.99966700	1.17682400
H	-6.32744700	-0.62445500	0.00750100

Cartesian coordinates of optimized geometries.

[o-ABA-Zn(H_2O)$_2$]$^{2+}$ in methanol.

C	2.75895600	-3.27969500	-0.82824500
C	1.42025200	-2.92409700	-0.72926300
C	1.03323500	-1.79711200	0.00356100
C	2.00945800	-1.03990700	0.66306600
C	3.35405800	-1.39188100	0.54746800
C	3.72985500	-2.50667300	-0.19198800
H	3.04188800	-4.15192400	-1.40402300
H	0.65943700	-3.51370000	-1.22715700
H	4.10410400	-0.79403100	1.05282400
H	4.77710700	-2.77304500	-0.26336900
N	1.64606000	0.13814000	1.41618500
H	2.39948200	0.40366900	2.04724000
N	-0.36545100	-1.52706800	0.11131400
C	-1.03067000	-0.38558300	-0.15875900
O	-0.45720000	0.68136400	-0.48766900
H	-0.94268000	-2.35257900	0.20828400
Zn	1.17088300	1.70609100	0.10795200
H	0.82547500	-0.02808500	1.99844900
C	-2.51343700	-0.42934000	-0.08146700
C	-3.23902900	0.48875500	-0.85317200
C	-3.19713400	-1.34442400	0.73257800
C	-4.62921900	0.47303700	-0.83072800
H	-2.70708500	1.19649900	-1.47555300
C	-4.58833500	-1.34653300	0.76013200
H	-2.66034900	-2.03192400	1.37614800
C	-5.30560100	-0.44390300	-0.02508100
H	-5.18503900	1.17493300	-1.44075200
H	-5.11052100	-2.04729800	1.40017000
O	2.43514700	2.72092400	-1.18177500
H	2.88052100	2.24447800	-1.89492500
H	2.05439100	3.52359900	-1.56321800
O	0.40736600	3.59201900	0.57263800
H	-0.54978100	3.67892500	0.67933000
H	0.81455700	4.07205200	1.30662400
H	-6.38910600	-0.45213800	-0.00527100

Cartesian coordinates of optimized geometries.

[o-ABA-Zn(H_2O)$_4$]$^{2+}$ in gas phase.

C	5.48941300	-0.24889300	-0.00474000
C	4.75604100	0.53994200	0.88270700
C	3.37055800	0.44766700	0.91298200
C	2.70347200	-0.45255900	0.06666700

C	3.45011400	-1.24288600	-0.82558300
C	4.83485100	-1.13406800	-0.86222600
C	1.23347200	-0.54503500	0.15530200
O	0.54138800	0.42070800	0.57792600
N	0.65782800	-1.71337600	-0.22323600
C	-0.71344500	-2.09758700	-0.09247300
C	-1.75672000	-1.38801400	-0.70153500
C	-3.07153500	-1.84163100	-0.57511100
C	-3.35058300	-3.01026800	0.12935200
C	-2.31188200	-3.73325700	0.71459600
C	-1.00428700	-3.27706300	0.60147200
N	-1.49157300	-0.15928700	-1.42530700
H	5.26767700	1.21866300	1.55420900
H	2.80101800	1.04256500	1.61514500
H	2.96334400	-1.91320900	-1.52615700
H	5.40376200	-1.73443400	-1.56147000
H	1.30019500	-2.48133700	-0.37430800
H	-3.87609200	-1.30225600	-1.06583900
H	-0.19396600	-3.83298600	1.05997400
H	-0.66718700	-0.27425600	-2.01542800
H	-2.26918100	0.04307600	-2.05171400
H	-2.51792000	-4.64884500	1.25457800
Zn	-1.10666800	1.44177300	-0.04000600
O	-0.04734100	2.74507100	-1.50513300
H	0.66306800	3.29853800	-1.15509600
H	0.09648100	2.68580500	-2.45827700
O	-2.53622500	0.73878200	1.44766200
H	-2.42810100	0.95874900	2.38205900
H	-2.84426200	-0.17890300	1.40915200
O	-0.41694700	2.89311500	1.39535200
H	0.33815500	2.66100000	1.95110500
H	-0.78164100	3.72946100	1.71084200
O	-2.76935200	2.68818500	-0.57768800
H	-2.74295200	3.46727300	-1.14742600
H	-3.62301100	2.66845200	-0.12631800
H	-4.37217500	-3.36293900	0.20049900
H	6.57005400	-0.17393800	-0.02898900

Cartesian coordinates of optimized geometries.

[o-ABA-Zn(H_2O)$_4$]$^{2+}$in water.

C	5.44688700	-0.30339000	-0.03010800
C	4.71882500	0.46543500	0.87888800
C	3.33400500	0.35231200	0.93507200
C	2.66581300	-0.54618100	0.09208800
C	3.40142200	-1.31291800	-0.82331000
C	4.78620600	-1.18736400	-0.88323500
C	1.18534500	-0.63423300	0.19888500
O	0.52140700	0.32237300	0.65678100
N	0.60080000	-1.78571800	-0.20032000
C	-0.78210200	-2.12141900	-0.08856000
C	-1.78804500	-1.35696800	-0.69614600

C	-3.12146500	-1.75486900	-0.57050700
C	-3.45254500	-2.91169500	0.12800200
C	-2.44966100	-3.68836400	0.70667400
C	-1.12282300	-3.29114000	0.59631500
N	-1.46580300	-0.14738300	-1.40310100
H	5.22986800	1.15307800	1.54213500
H	2.76318600	0.94603800	1.63684200
H	2.90615600	-1.98171700	-1.51750800
H	5.34690800	-1.77334700	-1.60142600
H	1.21962100	-2.56314700	-0.39110500
H	-3.89813300	-1.16382000	-1.04329700
H	-0.33476700	-3.87930100	1.05153600
H	-0.64956400	-0.28600200	-1.99785300
H	-2.23132700	0.12285200	-2.01726000
H	-2.69771100	-4.59344800	1.24676600
Zn	-1.04863100	1.48691800	-0.05634600
O	0.10392900	2.70566500	-1.42893200
H	0.69730400	3.30523500	-0.95722700
H	0.59748200	2.36416500	-2.18535000
O	-2.47357400	0.90717900	1.47405100
H	-2.18468300	0.97372100	2.39350900
H	-2.93688800	0.06166600	1.39807900
O	-0.33245800	2.97461300	1.31817600
H	0.28354100	2.68655400	2.00426700
H	-0.89510600	3.65546800	1.70889500
O	-2.66970900	2.67764800	-0.74325100
H	-2.47397800	3.48575500	-1.23455000
H	-3.39047200	2.87440900	-0.13129100
H	-4.49108400	-3.20850400	0.20738700
H	6.52565600	-0.21055100	-0.07715900

Cartesian coordinates of optimized geometries.

[o-ABA-Zn(H_2O)$_4$]$^{2+}$ in methanol.

C	5.44860000	-0.30107500	-0.03008500
C	4.71964000	0.47373300	0.87301300
C	3.33490300	0.36027800	0.92923100
C	2.66743100	-0.54433200	0.09210900
C	3.40422300	-1.31709000	-0.81749400
C	4.78888100	-1.19132900	-0.87740100
C	1.18728000	-0.63286200	0.19916000
O	0.52249800	0.32406700	0.65601200
N	0.60262000	-1.78471700	-0.19884200
C	-0.78063100	-2.11954200	-0.08752900
C	-1.78503900	-1.35661500	-0.69938900
C	-3.11879000	-1.75381900	-0.57551800
C	-3.45164400	-2.90821200	0.12628600
C	-2.45028300	-3.68296000	0.71004900
C	-1.12310000	-3.28666200	0.60083100
N	-1.46099500	-0.14758100	-1.40700400
H	5.23003800	1.16602300	1.53187400
H	2.76360400	0.95832100	1.62690300

H	2.91027600	-1.99112300	-1.50762200
H	5.35036700	-1.78222600	-1.59089400
H	1.22134000	-2.56276800	-0.38754300
H	-3.89436100	-1.16491500	-1.05279400
H	-0.33625300	-3.87366300	1.05959800
H	-0.64166600	-0.28579400	-1.99763200
H	-2.22398400	0.12113700	-2.02499200
H	-2.69977300	-4.58603300	1.25278200
Zn	-1.04875700	1.48514000	-0.05515600
O	0.10305200	2.70514400	-1.42755900
H	0.69158900	3.31192500	-0.95904000
H	0.59759600	2.36774900	-2.18511600
O	-2.47803700	0.89917500	1.46984500
H	-2.19058800	0.94952800	2.39077900
H	-2.94875800	0.05891700	1.38065500
O	-0.34047600	2.97353700	1.32206100
H	0.27695900	2.68948800	2.00853700
H	-0.90342700	3.65560900	1.71018200
O	-2.67335500	2.67028800	-0.74143600
H	-2.48776600	3.47412400	-1.24343200
H	-3.39915600	2.86396900	-0.13455600
H	-4.49033700	-3.20487400	0.20394900
H	6.52734500	-0.20825700	-0.07698900
